# Integrated assessment modelling as a positive science: private passenger road transport policies to meet a climate target well below 2 ^∘^C

**DOI:** 10.1007/s10584-018-2262-7

**Published:** 2018-09-03

**Authors:** J.-F. Mercure, A. Lam, S. Billington, H. Pollitt

**Affiliations:** 1 Department of Environmental Science, Radboud University, PO Box 9010, 6500 GL Nijmegen, The Netherlands; 2 Cambridge Centre for Environment, Energy and Natural Resource Governance (C-EENRG), University of Cambridge, The David Attenborough Building, Pembroke Street, Cambridge, CB2 3QZ UK; 3 Cambridge Econometrics Ltd, Covent Garden, Cambridge, CB1 2HT UK; 4 Department of Economics, Faculty of Social Sciences, Humanities and Social Science Building, University of Macau, E21, Avenida da Universidade, Taipa, Macau, China

## Abstract

Transport generates a large and growing component of global greenhouse gas emissions contributing to climate change. Effective transport emissions reduction policies are needed in order to reach a climate target well below 2 ^∘^C. Representations of technology evolution in current integrated assessment models (IAM) make use of systems optimisations that may not always provide sufficient insight on consumer response to realistic policy packages for extensive use in policy-making. Here, we introduce FTT: transport, an evolutionary technology diffusion simulation model for road transport technology, as an IAM sub-component, which features sufficiently realistic features of consumers and of existing technological trajectories that enables to simulate the impact of detailed climate policies in private passenger road transport. Integrated to the simulation-based macroeconometric IAM E3ME-FTT, a plausible scenario of transport decarbonisation is given, defined by a detailed transport policy package, that reaches sufficient emissions reductions to achieve the 2 ^∘^C target of the Paris Agreement.

**Electronic supplementary material:**

The online version of this article (10.1007/s10584-018-2262-7) contains supplementary material, which is available to authorized users.

## Introduction

Road transport emits 17% of global greenhouse gas (GHG) emissions, a flow of carbon that has grown historically by 2–3% every year over the past 20 years (IEA 2015a). Transport also uses a major proportion of oil produced worldwide: 48% of global oil extraction powers one form or another of motorised road transport (IEA 2015b).^1^ While developed economies (e.g. USA, Japan) typically have low transport activity growth, middle-income nations (e.g. Brazil, China, India) have fast growth rates (EI 2015). Policy for transforming the environmental impact of transport is a key area to model in detailed integrated assessment models (IAMs).

Traditionally, IAMs with high detail in energy end-use technologies have been based on system cost-optimisation or maximisation of the utility of the representative agent.^2^ The optimisation methodology used in IAMs is useful from a normative perspective as it helps map out feasible space and determine what are desirable configurations from a societal point of view (e.g. see IEA-ETSAP 2016a). For instance, optimisation can be useful in a context of agenda setting. The carbon price is typically used as a *control* parameter that internalises the climate externality, which moves the solution in technology space towards decarbonisation.

However, optimisation interpreted in a strictly positive scientific sense implies assuming consumers with infinite information about the whole system and no preferences tied to the social context. In that work philosophy, such a representation may be deficient, as it seems unlikely, from a behavioural science point of view, that choices of consumers could be incentivised and coordinated by the chosen policy signal (the externality price) in exactly the way that results from an optimisation calculation (Mercure et al. 2016). Optimisations interpreted as positive descriptions may not be reliable for use for impact assessments of policy scenarios, particularly if the modelled behaviour of agents is not sufficiently well informed.

There are two major issues with current optimisation-based IAMs (Wilson et al. 2015; McCollum et al. 2016; Pettifor et al. 2017b; Mercure et al. 2016):

1. Many IAMs are employed using typically one single policy lever for decarbonisation: the carbon price (through assumed emissions trading), which is applied to all emitting sectors including road transport. Real-world climate policy, however, features a much richer diversity of sector-specific incentives, particularly in transport, where carbon pricing is generally not used.
2. The collective response of agents to policy incentives (and their degree of access to/interest in reliable relevant information) is assumed to be coordinated in such a way that a system cost minimum or utility maximum is realised. In the real world, however, agents are far from being coordinated in a total system cost perspective, but instead, act according to specific behavioural features that do not usually feature in IAMs. For instance, no real decision-maker anywhere faces the global energy system cost and related presumed trade-offs.

The question we ask then is, what kind of methodology could solve these problems, that could be used at the scale of IAMs? Would using a different model structure enable to model more detailed and multiple policy instruments, including their interactions? Can we make model projections more consistent with recent technology diffusion data? To address these questions, we introduce a new type of evolutionary model that simulates the diffusion of transport technology, FTT: transport, as a sub-module of the IAM named E3ME-FTT-GENIE (see Mercure et al. 2018a, b, for details of the IAM itself). FTT models the diffusion of innovations calibrated on recent diffusion data and observed cost-distributions as a representation of consumer heterogeneity. It offers a highly detailed set of possible policy packages. Its strong path-dependence and high policy resolution allows to assess policy interactions explicitly, with a modelling horizon of 2050.

In Section 2, we summarise the theoretical background and empirical basis of the model. In Section 3, we show plausible endogenous projections of low-carbon vehicle diffusion as a result of specific transport policies for fast decarbonisation consistent with a target well below 2 ^∘^C. We conclude with a methodological recommendation for policy-relevance. We provide a detailed model description and its parameterisation in the Supplementary Information (SI).

## Background, model and method

### Behavioural information

Work is now developing to improve behavioural representations in IAMs (Wilson et al. 2015; McCollum et al. 2016; Pettifor et al. 2017a, b). However, in order to effectively inform policy-making, it is also crucial to clearly delineate normative (i.e. “tell me what are the components and I will tell you the best way to organise the system”) from positive (i.e. “tell me the context and I will predict what people will choose”) modelling philosophies.

**Fig. 1:**
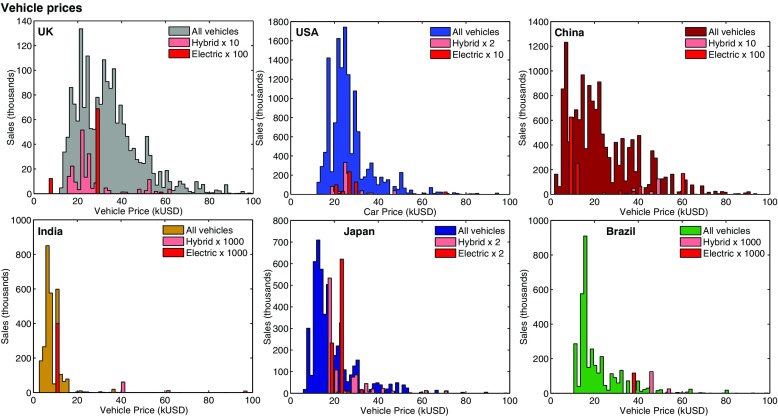
Price data for vehicles in six major economies, reproduced from (Mercure and Lam 2015)

Of interest here, passenger road transport is not normally covered by a carbon price, but many other policy types are used (regulatory, push and pull policies, see e.g. ICCT 2011). The ‘cost’ of vehicles as mitigation options in the traditional modelling sense is not very well defined since the (lognormal) frequency distribution of vehicle prices spans a range often much larger than its average (see Fig. 1 and the data in Mercure and Lam 2015, and more data in SI Section 5.1). The heterogeneity of vehicle consumers is large.

To better understand this requires using tools and knowledge from behavioural economics as well as marketing research, which has been largely overlooked by IAM modellers. Wilson et al. (2015); McCollum et al. (2016); Pettifor et al. (2017a, b) make a compelling argument for the inclusion of significantly more behaviourally relevant information and functionality in existing IAMs, including a particular emphasis on heterogeneity, social influence, and the number of policy instruments represented. This led to the development of a new behaviourally rich model (Pettifor et al. 2017a, b). This plea applies to markets for private vehicles, where the heterogeneity of consumers is high (Mercure and Lam 2015) and social influence dynamics, typically not modelled, may well have as much explanatory power as prices (e.g. McShane et al. 2012; Wilson et al. 2015; Pettifor et al. 2017a). However, existing technological trajectories are also important to consider, due to their momentum (see e.g. Geels 2005).

Whether agents are believed to minimise costs or not may not be the issue to resolve: the result of agents individually optimising their costs and benefits *does not* necessarily lead to a cost optimum at the system scale, i.e. to an optimising representative agent/social planner (Kirman 1992). Mercure et al. (2016); Mercure (2018) show that, in a quantitative (as well as qualitative) social theory, as soon as agents interact with one another and *value* the behaviour of other agents when making consumption decisions, fads, fashions, bandwagon effects arise that violate the premise of systems optimisation. These effects break the connection between cost/utility-optimisation at the individual agent level, and optimisation at system level, partly because the representative agent cannot exist.^3^ Multi-agent influence instead *leads to diffusion dynamics* (as in Rogers 2010; see also the work on information contagion by Arthur and Lane 1993; Lane 1997).

Wilson et al. (2015) and Pettifor et al. (2017a) review an extensive body of knowledge on social influence in vehicle choice. It generates a system in which the state of diffusion is not a simple function of input prices, but instead depends on the order of past events (path-dependence, see Arthur 1989; Arthur et al. 1987). In systems optimisation, such increasing returns generates multiple solutions and model instabilities (Gritsevskyi and Nakićenovi 2000): which ones are the ‘correct’ optimal outcomes? Broadly speaking, social influence ‘attracts’ agents towards the adoption of popular innovations and away from unpopular ones, despite absolute costs and benefits (Arthur 1989; Arthur and Lane 1993; Brock and Darlauf 2001a, b).^4^ The value ascribed by consumers to the choices of others can be as influential to their choices as the sum of the characteristics of the goods themselves.

This effect is not only generated by social influence: if one allows that the availability of technology to agents may be restricted by existing market shares (availability follows the size of the industry), which grow with technology diffusion (the more technologies diffuse, the more agents can access them), then market shares partly determine the pace of diffusion, a recursive problem of the same form. In this interpretation, each agent has a different set of knowledge of technology options, stemming from access to a different set of options. This makes the use of a representative agent impossible. Technology producers expand production capacity following demand growth, and demand grows following technology availability. In fact, in a model, it is not straightforward to empirically attribute the effects of social influence, technology diffusion and industry growth dynamics. Whichever the root source, these dynamical effects are mathematically crucial to represent correctly, as they determine whether a model assumes that agents behave in a perfectly coordinated way or not (i.e. whether a representative agent exists or not).

It is therefore potentially insufficient to better parameterise basic optimisation models with additional consumer behavioural information, if the methodology remains tied to optimisation at the system level, which precludes bandwagon effects by construction. It is noteworthy that the same recursive effects arise in animal population ecology (Kot 2001), and in fact one finds that evolutionary modelling methods can achieve realistic consumer representations with behavioural heterogeneity and social influence (Young 2001), without the use of representative agents. We describe one here, deployed at IAM scale.

### Technology diffusion as bandwagon effects

The FTT model uses a modified version of discrete choice theory in the form of an evolutionary theory. It uses observed distributions of costs to represent agent heterogeneity (a form of revealed preferences). It is based a on dynamical systems approach as opposed to systems optimisation, minimising perceived costs at a bounded-rational agent level as opposed to minimising whole system costs in standard models. After providing some theoretical background, we describe the methodology in this section.

Discrete choice theory (DCT, Anderson et al. 1992; Domencich and McFadden 1975; Ben-Akiva and Lerman 1985) is the main workhorse to regress choice by non-interacting heterogeneous agents. Agents in a DCT model are assumed to have knowledge of, and have access to all options available in the market (perfect information). The resulting multinomial logit (MNL) can also be mathematically derived from a problem of utility maximisation under a budget constraint when utility follows a constant elasticity of substitution (CES) model (Anderson et al. 1992), where the elasticity is related to the heterogeneity of agents. MNLs, CES and optimisation models^5^ thus share a common theoretical foundation, in which agents do not interact with one another, and base choices on infinitely detailed information. These models do not endogenously generate diffusion profiles consistent with what is observed (S-shaped curves, e.g. see Nakicenovic 1986; Grübler et al. 1999), unless externally constrained to (e.g. by just the right carbon price^6^).

In diffusion problems, it is specifically the case that agents *do not* have (or wish to have) access to or knowledge of all existing options in the market, since some options are largely unknown/untried innovations with small market shares and small production capacities, and thus limited access. Production capacity for new technologies are not expanded instantaneously to respond to changes in consumer demand; they co-evolve over time. Widely used products have a higher capacity for diffusion, as they are more visible and they have a larger producing industry (Bass 1969; Fisher and Pry 1971; Mansfield 1961; Sharif and Kabir 1976; Marchetti and Nakicenovic 1978). These properties are core elements of innovation diffusion theory (Rogers 2010).^7^ Bandwagon effects are a key component of transitions theory (e.g. Geels 2002, 2005; Rotmans 2001; Turnheim et al. 2015), and agent-based models (ABM, see Kohler 2009; Holtz 2011; Holtz et al. 2015).

Including interactions between agents (agents learning from each other, i.e. social influence) in a discrete choice model leads to diffusion dynamics of products in markets (Mercure 2018, see also (Arthur and Lane 1993); and SI Section 3.3). Here, we use the so-called ‘replicator dynamics’, a mathematical system used in evolutionary theory to describe the selection process in evolutionary problems (evolutionary game theory, Hofbauer and Sigmund 1998), derived in detail in SI Section 3.3, summarised here (see Mercure 2018; 2015; Safarzynska and van den Bergh 2010 and Young 2001, 2009 for broader discussions). Its dynamical behaviour is consistent with empirical diffusion observations (e.g. Mansfield 1961; Fisher and Pry 1971; Nakicenovic 1986).

### A bounded-rational discrete choice model with heterogenous agents

Consumers in vehicle markets are highly heterogenous, and this heterogeneity varies by country, shown in Fig. 1 (see SI Section 5.1 and Mercure and Lam (2015)). This heterogeneity can be observed, amongst many other ways,^8^ through differentiated prices, which typically increase exponentially with linearly increasing engine sizes (vehicle power, *ibid*). Taking account of this heterogeneity is crucial in models to quantify the impact of pricing policies on rates of adoption (e.g. subsidies). Indeed, if the distribution of prices spans an order of magnitude, then purchase and/or fuel tax schemes will generate widely different levels of incentives in different market segments, and the diffusion of new technologies often starts in more affluent segments of the population. This can be modelled by using distributed variables.

In this work, heterogeneity is ‘observed’ from the market (Fig. 1) because markets, consumers and regulation co-evolve: entrepreneurs strive to better match the differentiated tastes of consumers, while consumer tastes are influenced by how the market evolves. Observed distributions of prices reflect consumer taste heterogeneity, related to a myriad of socio-economic contextual variables (income, geography, culture, etc), which change over time.

It is not necessary here to track every individual agent or agent type in order to represent heterogeneity: DCT statistics can be used. ABMs do so, but using DCT is computationally faster. However, in our bounded-rational model, agents do not know every vehicle model type in the market (i.e. we reject perfect information)^9^ but, rather, consumers choose within various subsets of the market. This means that every agent has a different set of knowledge, which violates the premise of the standard MNL. Instead, modelling this is done using chains of binary logits, with pair-wise comparisons of options, each *weighted according to the number of agents* carrying out these comparisons. These weights are the market shares of each vehicle type, reflecting the probabilities of consumer learning events, for example through visual influence (as in McShane et al. 2012).

In such chains of binary logits, agent preferences between pairs are treated as distributions of the perceived costs and benefits of technologies (the generalised cost), and compared at every time step of the model. When faced with a choice between vehicle categories *i* and *j*, a fraction of agents making the choice will prefer technology *i*, denoted $F_{\text {ij}}$, while the rest will prefer *j*, denoted $F_{\text {ji}}$, where $F_{\text {ij}} + F_{\text {ji}} = 1$. Denoting that option *i* is perceived by that subset of agents to have a generalised cost $C_{i}$ that follows a frequency distribution $f_{i}(C-C_{i})$, and cumulative distribution $F_{i}(C-C_{i})$, with mean $C_{i}$ and standard deviation $\sigma _{i}$ (and similarly for option *j*), the fraction of agents making the choice preferring *i* over *j* is as follows:

1 $$ F_{\text{ij}}({\Delta} C_{\text{ij}}) = {\int}_{-\infty}^{\infty} F_{j}(C) f_{i}(C-{\Delta} C_{\text{ij}}) dC, \quad {\Delta} C_{\text{ij}} = C_{i}-C_{j}, $$

which, if $f_{i}$ is a double exponential Gumbel distribution (as in standard DCT), yields the classic binary logit (see Domencich and McFadden 1975). The standard deviation is treated using the standard error propagation method:

2 $$ F_{\text{ij}} = \frac{1}{1 + \exp\left( {\Delta} C_{\text{ij}} / \sigma_{\text{ij}}\right)}, \quad \sigma_{\text{ij}} = \sqrt{\sigma_{i}^{2} + \sigma_{j}^{2}}. $$

This is a logistic function of the ratio of the mean cost difference to the width of the price distribution (SI Section 3.2). Any noticeable changes in aggregate preferences requires any perceived cost difference to be larger than the combined standard deviations. This is how, in choice models, rates of diffusion relate to heterogeneity, and is one way to model heterogenous agents that cost-minimise individually, within their context, under social influence, without using any systems optimisation algorithm. Price distributions, such as in Fig. 1 are used for parameterising $f_{j}(C)$. FTT is thus parameterised by cross-sectional datasets (SI Section 5.1).

### The replicator dynamics equation of evolutionary theory

We take $S_{i}$ as the market share of option *i* (the number of units of type *i* in the fleet, with respect to the total). We evaluate exchanges of market shares between technology categories as time goes by, the magnitude of which is determined by preferences $F_{\text {ij}}$, while the rate originates from the fleet turnover. At each time step dt, the amount of shares flowing away from category *i* into category *j* is proportional to the number of vehicles of type *i* requiring replacement, itself proportional to the market share $S_{i}$. The number of agents replacing vehicles of type *i* exploring the possibility of purchasing a vehicle of type *j* is a subset of all agents who have access or have reliable knowledge of option *j*, which is proportional to the market share of option *j* (see Mercure 2015). Being probabilistic, shares flow simultaneously in opposite directions but with typically unequal magnitude (if preferences are exactly 50%/50%, then the net flow is zero). The expression that results for the net flow is the replicator dynamics equation (also called Lotka-Volterra, SI Section 3.3):

3 $$ {\Delta} S_{j \rightarrow i} = S_{i} S_{j} \frac{F_{\text{ij}}}{\tau_{i}} {\Delta} t,\quad \Rightarrow \qquad \frac{dS_{i}}{\text{dt}} = \sum\limits_{j} S_{i} S_{j} \left( \frac{F_{\text{ij}}}{\tau_{i}} - \frac{F_{\text{ji}}}{\tau_{j}} \right).  $$

This is a dynamical equation that is path-dependent and hysteretic (Mercure 2018).^10^ Costs and policy incentivise agents to make choices that orient the trajectory of diffusion, and the trajectory has momentum.^11^ Costs are influenced by learning curves, typically stronger for new technologies, reinforcing diffusion and path-dependence. The mathematics describe a system in perpetual flow without equilibrium, and indeed, problems of technology diffusion do not have steady states.^12^ Innovations come and go, as the popularity of novelty products rises and later declines. This equation is derived in detail in two distinct ways in the SI Section 3.3.

### Cost distributions database and micro-model of vehicle consumer choice

Price distributions for private vehicles are typically log-normally distributed (see Fig. 1, Mercure and Lam 2015 and SI section 5.1). Cost-comparisons in the FTT binary logit are thus made between cost distributions in logarithmic space, using an appropriate transformation (SI Section 3.2). Consumer decisions are not made solely based on vehicle prices; future operation and maintenance costs are taken into account, with a discount rate, as well as non-pecuniary benefits. It can never be fully clear what intuitive or quantitative evaluations are carried out by vehicle consumers when taking decisions (and evaluation methods may differ across the population). For modelling tractability, we require a suitably general, statistical and flexible micro-model that can encompass all sorts of heterogenous behaviour. We use comparisons of the net-present values in log scaling, which we denote as the Levelised Cost Of Transportation (LCOT). It expresses a discounted cost of generating a unit of transport service:^13^

4 $$ \log \left[ \sum\limits_{t = 0}^{\tau} \frac{I_{i} + \text{VT}_{i} + \text{CT}(\alpha_i) + Fu_{i}(t)\text{FT}(\alpha_i,t) + \text{MR}_{i} + \text{RT}_{i}(t)}{(1+r)^{t}} \left/ \sum\limits_{t = 0}^{\tau}\right. 1/ (1+r)^{t} \right] +\gamma_{i},  $$

where time *t* refers to moments in a hypothetical future at which agents expect costs to take place during vehicle type *i*’s lifetime $\tau $ (i.e. not real time), *r* is the consumer discount rate, $I_{i}$ is the vehicle price, $\text {VT}_{i}$ is a vehicle specific one-off registration tax, $\text {CT}(\alpha _{i})$ is a registration tax based on the fuel economy *α*_*i*_, $Fu_{i}$ is the expected fuel costs, FT is the fuel tax, $\text {MR}_{i}$ is repair costs and $\text {RT}_{i}$ is a yearly road tax. The $\text {LCOT}_{i}$ is the mean of the combined distributions of these cost components.^14^ It is paired with its standard deviation ΔLCOT_*i*_, calculated using the root of the sum of the squares of all variations. Phase-out regulations are approximated by setting $F_{\text {ij}} = 0$, i.e. overriding consumer choices, preventing further sales of a particular vehicle category (see SI Section 3.5 for details on our policy representations).

The costs explicitly represented in the above equation are not sufficient to realistically model technology diffusion, since many other pecuniary and non-pecuniary costs are valued by agents, as we find empirically, for which we have no explicit data, to explain observed technological trajectories. An adjustment to this equation is necessary in order for FTT: transport to match diffusion trajectories observed in recent years (see our global historical diffusion database, SI section 5.2). Since FTT is a path-dependent simulation, its formulation would be inconsistent if it suggested a change of diffusion trajectory at the start of the simulation. Indeed, to be self-consistent, historical data *must* determine the diffusion trajectory in the first few years or decade of the simulation.

An additional parameter is determined empirically, $\gamma _{i}$, which represents all unknown constant pecuniary and non-pecuniary cost components, and policies in place, that are not explicitly represented or included in Eq. 4, needed to match the modelled diffusion trajectory to the observed trajectory, in order to ensure consistency with diffusion theory. $\gamma _{i}$ has the unique value set that makes the diffusion rate (dS_*i*_/dt) continuous across the transition from historical data to simulated data for $S_{i}$ at the start year of the simulation. $\gamma _{i}$ is determined with a methodology described in SI section 5.5. As with econometric parameters, $\gamma _{i}$ is assumed not to change over the simulation period. This is not necessarily fully satisfactory; however, there exists no reliable scientific basis upon which to predict distant future changes in $\gamma _{i}$, which we consider best of current knowledge.

### The FTT: transport database

Data gathering for the FTT:transport vehicle price database is described in detail in (Mercure and Lam 2015) and SI Section 5. Light duty vehicle types were classified as petrol and diesel, compressed natural gas (CNG), hybrid, electric vehicles (EV) and motorcycles. Each category was sub-divided into three consumer classes: economic (*Econ*, below 1400cc), mid-range (*Mid*, between 1400 and 2000cc) and large engine vehicles (*Lux*, above 2000cc),^15^ each of which has its own vehicle price distribution as an explicit representation of agent heterogeneity (see SI Section 2 for detailed UK data). We stress that it is not the engine size classification that we ascribe to heterogeneity, but rather, the fact that prices are distributed, whereas the engine size classification mainly serves presentational purposes. Motorcycles were divided as either above or below 125cc. Hypothetical future higher efficiency vehicle categories are added using scenario defined fuel efficiencies based on current targets.^16^

2012 data for new registrations per vehicle model type were obtained from either national statistics or from (Marklines 2014) and matched, model by model, to recent prices obtained online (Mercure and Lam 2015). Vehicle price data were matched to sales numbers for 18 representative regions, used as proxies for 53 out of E3ME’s 59 regions based on economic and regional similarities, following data availability. Data for other countries were used by proxy based on market similarities (SI section 5). Historical total yearly distances driven nationally and total numbers of vehicles registered in national fleets were obtained from (EI 2015; Eurostat 2015). Historical shares per vehicle category for 53 E3ME regions were obtained by merging several datasets (EI 2015; Marklines 2014; Eurostat 2015), and cover 2004 to 2012, while total fleet sizes and yearly sales cover 1990 to 2012 (detailed procedure given in SI Section 5.2, the historical data itself provided separately in the Suppl. Excel data file).

### Projecting vehicle sales, fuel use and emissions with E3ME

FTT:transport is built as a sub-module of E3ME (see Cambridge Econometrics 2014), itself able to calculate global emissions and coupled to the climate model GENIE1 (Holden et al. 2013), making it a fully detailed IAM (see Mercure et al. 2018a, b for a full model description). E3ME is a non-equilibrium macroeconometric simulation model based on a demand-led Post-Keynesian structure (Pollitt and Mercure 2017), theoretically coherent with the evolutionary simulation basis of FTT. The degree to which vehicles are used is assumed not to depend strongly on their types of engines, and is calculated by regressing total vehicle use (in veh-km/y) with respect to fuel prices and income, and projecting these to 2050, using fuel prices and income endogenously determined by E3ME. The number of vehicles purchased does not strongly depend on vehicle type composition of the fleet, and thus vehicle sales are regressed and projected against income and average vehicle prices, the first endogenously determined by E3ME.^17^ Elasticities from the literature were used to constrain regression parameters and avoid spurious results. Fleet sizes are calculated using projected sales and a survival function derived from DVLA (2012a) data (SI Section 4.3).^18^

Resulting demand profiles vary substantially across regions. As a general rule, fast growing economies with fast growing fleets (e.g. China, India, Brazil) have a higher response to price changes than slow-growing developed economies where fleets do not grow (e.g. UK, USA), which applies to both the demand for vehicles and the demand for travel (SI Section 5.4).

FTT is fully integrated to E3ME with several dynamical feedbacks to the global economic simulation. In E3ME, income, prices, fuel use, investment, employment and more quantities are calculated endogenously globally, in 59 regions, 70/44 sectors (EU/non-EU countries), 23 fuel users and 12 fuels. E3ME calculates global fuel use and combustion emissions, where fuel use for electricity generation is simulated using the sister model FTT:power. Thus, the combination of FTT:power, FTT:transport, FTT:heat and E3ME provides a relatively high definition dynamical coverage of global fossil fuel use and emissions. Disposable income is calculated based on wages, GDP, price levels and employment. Fuel prices are derived from endogenous dynamical fossil fuel depletion and cost calculations (see our model in Mercure and Salas 2013). Fuel use from road freight transport is accounted for, but there technological change is not modelled in as much detail; biofuel mandates form the main freight decarbonisation mechanism (see SI Section 3.4).

### Summary of improvements over incumbent models

We summarise here the novel improvements that FTT:transport provides over standard methods:

1. *FTT endogenously projects current diffusion trends with a path-dependent diffusion profile (S-shaped).*
2. *Diffusion is driven by choices of endogenously modelled heterogenous consumers under bounded rationality and social influence, not a representative consumer.*
3. *The diffusion trajectory is tied to recent historical data but does not strongly depend on technological assumptions.*
4. *A bounded-rational choice framework enables to model many forms of policy instruments and composite packages (currently eight different policy levers are implemented), and strong policy interaction is observed.*
5. *Diffusion trends cannot be made discontinuous by a sudden change or break in the policy regime, due to endogenous diffusion inertia.*

## Policy strategy and model results

### Policies for decarbonising private personal transport

Policies for transport decarbonisation currently take four forms: (1) improving the efficiency of conventional ICE vehicles, (2) promoting technological change towards lower emissions vehicles with alternate engine types, including kick-starting new markets, (3) substituting the fuel for lower carbon content alternatives (biofuel blends), and (4) policies to curb the amount of driving. In order to reach the 2 ^∘^C target with over 66% probability, global CO_2_ emissions must be reduced to well below 5.5 GtC in 2050 (Meinshausen et al. 2009; Zickfeld et al. 2009; Rogelj et al. 2013). Since road transport emissions make roughly 17% of emissions, transport emissions must likely be reduced to well below 1 GtC in 2050, starting from 1.5 GtC in 2016. This necessitates at least a partially electric composition of vehicle fleets, since calculated biofuel potentials are not guaranteed sufficiently large to replace the whole current use of $\simeq $ 170 EJ of liquid fossil fuels (Hoogwijk et al. 2009; Mercure and Salas 2012). Efficiency policies for conventional ICE vehicles are not likely sufficient to meet the 2 ^∘^C target. Using a combination of technology push, pull and regulatory policies appears *a priori* to be a reasonable strategy.

Efficiency standards are traditionally imposed using regulatory policy. In the model, this corresponds to controlling the nature of substitutions in new vehicle sales, leaving existing vehicles in the fleet to operate until the end of their statistical lifetime. This can be used in the model to force phase-in of a number of existing environmental innovations to existing conventional technologies, for instance targeting the fuel economy and phasing out older technologies (SI Section 3.5).

Purchase taxes or rebates are often used as a demand-pull policy to level the corporate playing field, and create space in the market for new, more expensive low-carbon technologies. Registration taxes can also re-allocate purchases along the price-engine size axis (Mercure and Lam 2015). If taxes applied to the vehicle price are made proportional to vehicle rated emissions, a ‘carbon tax’ results on future expected lifetime emissions of the vehicle. Meanwhile, a tax on fuels matches more closely an actual carbon tax, but may be less effective per dollar paid at influencing the type of vehicles purchased, depending on consumer time preference.

Promoting diffusion in markets where particular types of low-carbon vehicles do not exist, using price policies, does not typically work if manufacturers and infrastructure is not present to allow it. In this case, large institutions (e.g. government) can kick-start markets, where for example, public or private institutions purchase or impose the purchase of a fleet of a particular type (e.g. natural gas buses, electric municipality vehicles or taxis), jump-starting later diffusion, which would not happen otherwise. Such strategies are common in many countries (SI Section 3.5).

In FTT, policy formulations currently take eight possible forms: regulations, standards, registration/fuel/road taxes, subsidies, biofuel mandates, and public procurement (kick-start). As an example, we used several of these types of policies to create one possible coherent framework that achieves worldwide decarbonisation, with the following strategy (detailed numbers given in the Suppl. Excel data file):

1. Setting the fuel efficiency standard of new liquid fuel vehicles to amongst the best currently available, in each vehicle engine size class, with near term compliance deadlines.
2. Phasing out by regulation the sale of low efficiency liquid fuel vehicles starting in 2018.
3. Introducing electric vehicles in all markets in which they do not exist (in our historical data), in all consumer classes, with procurement policies by 2020.
4. Aggressively taxing the registration of new liquid fuel vehicles proportionally to rated emissions, in order to re-orient consumer choices (here we used 100$/(gCO2/km) in constant 2012USD), starting in 2020.
5. Increasing taxes on fossil liquid fuels to acquire better control of the total amount of driving (here we used a value increasing from $0.10 to $0.50 per litre of fuel between 2018 and 2050 in constant 2012USD).
6. Increasing biofuel blend mandates gradually until they reach up to 70% all regions in 2050.

One advantage of using a non-optimisation diffusion model is that policy interactions can be assessed explicitly, and synergies between instruments can be observed. Here, each of these layers of policy plays a specific role, and none of them can achieve decarbonisation task on their own; they influence the effectiveness of each other. Thus, they only work when applied simultaneously in a coordinated manner. For example, taxing registrations of vehicles based on emissions will drive consumers to the best available, and a key opportunity would be missed if only marginally higher efficiency vehicles were available for purchase. In this case, kick-start policies for EVs take a crucial role to enable the full effectiveness of taxes at reducing emissions, especially in developing countries. Furthermore, the biofuel mandate can only be increased to large values if the liquid fuel consumption of the fleet declines, otherwise the demand for biofuels could imply future issues of excessive land-use changes for biofuel production (e.g. see Searchinger et al. 2008; Fargione et al. 2008).

### Exploring the impact of policy strategy by layers

**Fig. 2:**
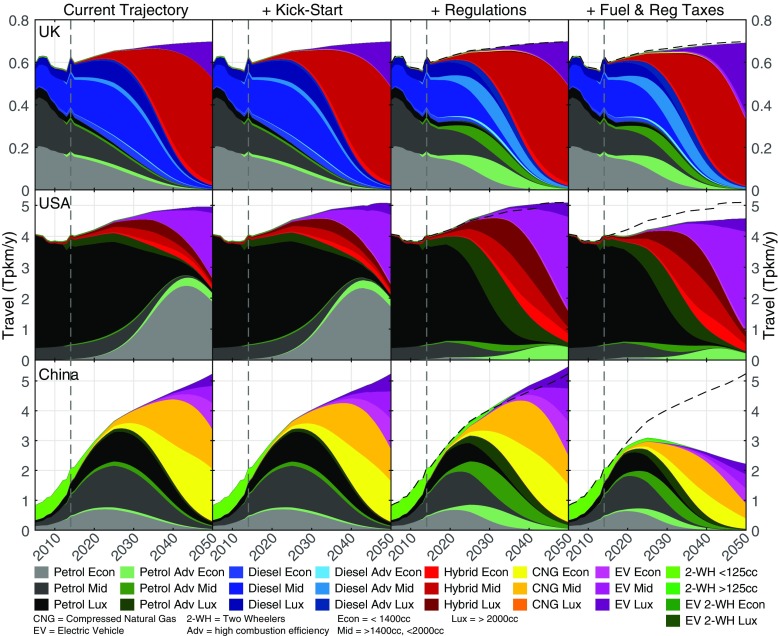
FTT transport generation (in Tera person-kilometres per year, Tpkm/y) by five technology types in three engine size classes in the UK, China and the USA. The simulation starts in 2012. Prior to this, historical data is shown. Differences in totals arise with tax policy, where consumers drive less. The black dashed lines reproduce the baseline total for comparison. Data to the left of the dashed lines are historical. Differences between panels with respect to the ‘Current Trajectory’ are given in SI Section 6.1

We explore in this section how the six steps above can deliver sufficient cuts, focussing on the UK, the USA and China (Fig. 2). The UK fleet has a significant number of diesel vehicles, a growing fleet of hybrids and a nascent diffusion of electric vehicles. China, dominated by petrol (gasoline) engines, sees its large fleet of motorcycles decline and an emerging diffusion of CNG vehicles. The USA is dominated by large conventional petrol (gasoline) engines, with growing hybrid and electric fleets. These trends, observed in our historical data, continue in the baseline scenario of FTT, in which a slowdown of consumption of liquid fuels already takes place due to existing diffusion dynamics of alternative engine vehicles having already acquired momentum. These baseline diffusion profiles lead to a globally peaking liquid fuel consumption in the 2030s, leading to stranded fossil fuel assets worldwide (see Mercure et al. 2018b, but not due to biofuels; described in the next section).

Substantial efficiency changes are currently taking place in vehicle fleets around the world, due to efficiency changes and the gradual adoption of hybrid, CNG and EV drivetrains. In FTT:transport, this is projected to reduce current emissions by 56%, 65% and 72% in 2050 in the UK, China and the USA respectively, in the baseline. It is to be noted that these changes are mostly the result of technological trajectories observed in recent historical data, which the model projects into the future, as no new policies are explicitly included in the FTT:transport baseline. Many policies currently being adopted or adopted recently will alter these trajectories. For instance, the rise of CNG in China is likely to become replaced by EVs with the support of new policies (Ou et al. 2017), but this is not included in our baseline.^19^ To accelerate that, policy for decarbonisation described above first involves regulations to phase out from the market less efficient engine types and force in emission standards across engine size classes (steps 1–2). Without other policies, this contributes additional reductions of 0%, 7% 13% over the baseline trends in 2050 for the UK, USA and China respectively, modest additional impacts effectively due to the modest efficiency targets achievable with ICE engines.

Tax policies are applied (policy steps 4–5) to both (1) rated emissions and (2) fuel consumption. Fuel taxes do comparatively little to incentivise changes of technology, mainly due to our average consumer discount rate of 15%.^20^ However, they contribute to curbing driving.

Taxes on registration of vehicles proportional to emissions per kilometre have a higher impact on guiding consumer choices towards low-carbon vehicles, in particular as they become more available through their diffusion: in FTT, the more they are adopted, the more the tax becomes effective at incentivising their adoption. EVs take considerable time to diffuse, and what is observed is that an intermediate layer of diffusion of intermediate emissions vehicles arises. In the UK and the USA, they are hybrids, while in China and India, they are CNG. The tax also incentivises changes of engine category; however, this is limited, as consumers can typically save more tax money by changing engine type rather than engine size, while their preference for vehicle class remains (due to the $\gamma _{i}$ parameters). With registration tax policies, the strategy must involve providing choice to consumers, as otherwise it only achieves raising tax income without sufficient change in emissions, particularly where EVs are not widely available. Note that similar results could be achieved using tax/feebate combinations.

It is useful, and possibly necessary in many regions, for the authorities to kick-start the EV market, by sectoral regulation or public procurement, where the industry and infrastructure is absent.^21^ We note that a kick-start policy nucleates simultaneously (i) a market, (ii) a network of supporting industry, and (iii) a social diffusion process, which subsequently co-evolve with the diffusion process itself. In many regions, sales of economic EVs are non-existent in the data, but this will not remain so indefinitely. In the model, mass diffusion of EVs takes-off after 2040, at which point the fuel consumption of the whole fleet declines substantially.

Remaining fossil fuel use is reduced further by the use of biofuel mandates at 70% (100%) by volume. Altogether, these combined policies lead to 88% (98%), 96% (99%) and 91% (91%) emissions reductions based on 2016 levels for the UK, the USA and China.

### Global road transport decarbonisation, fuel use and emissions

**Fig. 3:**
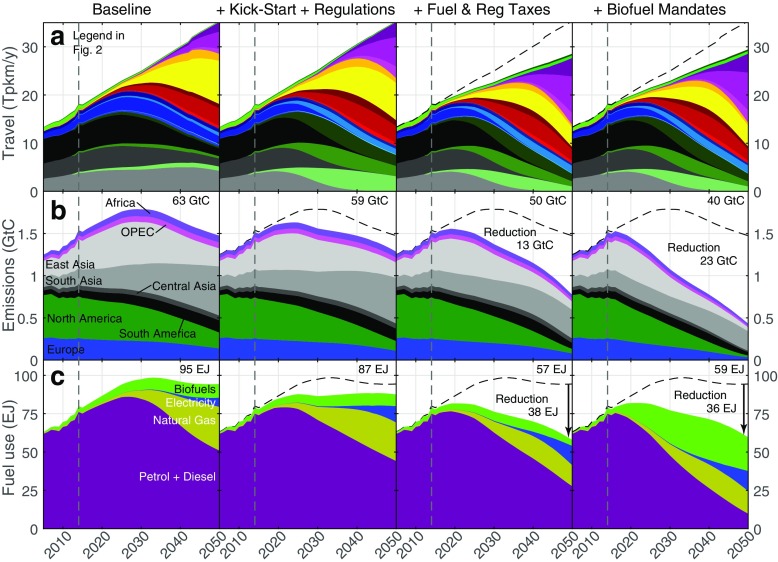
Global distance driven per vehicle category (in Tpkm/y, top row), global private passenger vehicle emissions in eight regions (in GtC/y) and global road transport fuel use per E3ME fuel type including freight. The black dashed lines reproduce the baseline total for comparison. Central Asia includes Russia and the former Soviet bloc excluding those in the EU; South Asia includes India and Indonesia; East Asia includes China and Japan. Data to the left of the dashed lines are historical

The composition of the global fleet is given in Fig. 3, top row. Given fleet turnover rates and existing trends, it is unlikely that emissions can be reduced with the diffusion of EVs alone sufficiently by 2050 to reach a climate target well below 2 ^∘^C or a 1.5 ^∘^C target.

Instead, emissions are reduced with successive waves of diffusion of innovations, of ever lower carbon intensity. Policy step 6 involves the use of relatively high biofuel percentage blends (70%, 20% is in the baseline) in liquid fuels, a policy that has been controversial in Europe and elsewhere (e.g. Searchinger et al. 2008; Fargione et al. 2008). Indeed, a high biofuel mandate does not appear realistic in the baseline. However, in a 2 ^∘^C scenario, by 2030, total liquid fuel use declines significantly due to the diffusion of more efficient combustion technologies, including hybrids, as well as CNG and EVs displacing conventional engines. Even when including freight transport, biofuel blend percentages can in fact be increased to 70% in 2050, while maintaining global liquid biofuel use for transport below 27 EJ.^22^ This enables to decarbonise road transport to below 0.43 GtC/y by 2050 (72% of 2016 global transport emissions, Fig. 3 middle row, 83% with 100% biofuel mandates).^23^ This is consistent with at least the 2 ^∘^C target, possibly even the 1.5 ^∘^C target, depending on emissions from other sectors.

Figure 3, bottom row, shows that the use of middle distillates peaks in the baseline, reflecting existing technology diffusion trends. With regulations, taxes and biofuel blends, the use of liquid fossil fuels for road transport declines to below 10 EJ/y (70% biofuel blends) or 1 EJ (100% biofuel blends) in 2050 (86% below the 2016 value with 70% biofuels, 99% with 100% biofuel blends), leading to drastic reduction of demand for crude oil (Mercure et al. 2018b). Biofuel use due to the biofuel mandate remains below 27 EJ with baseline blends, and below 38 EJ with 100% blends, peaking in around 2040 due to fleet efficiency improvements and displacement of the combustion engine by other technologies. The use of natural gas remains comparatively low (< 18 EJ), due to a relatively low global share of CNG. The use of electricity, in a scenario where electric cars make up 33% of the fleet in 2050, remains comparatively small at 14 EJ, (with respect the E3ME 2050 total electricity demand of 140 EJ), due to the very high conversion efficiency of EVs. The result is that transport electrification significantly reduces global energy use, and does not imply an excessive or unmanageable new load for the power sector.^24^ Reaching the 2 ^∘^C target remains, however, contingent on power generation and land-use decarbonising.^25^

### Reflections on the model

Standard cost-optimisation models are normative and search parameter space for a system state that achieves a set of system and political objectives. Meanwhile, a positive model offers a representation that attempts to guess what future states of an existing system may look like, given its present state and evolution trajectory, and decisions taken to alter that trajectory. In the world of IAMs, almost all models are of the normative optimisation type, and often have relatively low policy resolution. However, the development of new climate policy requires, in most national policy processes, impact assessment of detailed policy frameworks. This unavoidably demands the use of positive models that model complex policy packages and can give policy-makers indications of current trajectories and potential outcomes of the various policy options considered.

Here, we have shown that this can be achieved, but with a different type of modelling framework, in comparison to standard methods. We used a model without representative agent, based on dynamical systems without equilibrium, to explore the evolution of the global road passenger vehicle fleet, based on trajectories observed in our historical database. We found that indeed, results are different from those using standard methods. For example, EVs diffuse faster than in optimisation models, even when these include substantial amounts of behavioural information (Pettifor et al. 2017b) in both the current trajectory, and in a decarbonisation scenario (more comparisons to other models given in SI Section 6.2). Furthermore, oil demand for transport peaks in the current trajectory, substantial efficiency changes are already taking place due to the popularity of new technologies, such as hybrids. Thus, we can expect that using this type of method can provide a critical lens with which to look at all types of models used for advising policy-making, in particular IAMs.

Perhaps the key advantage of this model is that outcomes are more dependent on observed technological trajectories, and less reliant on technological assumptions, such as costs, in comparison to optimisation models. We demonstrate this in SI Section 6.1 with an extensive sensitivity analysis. We observe that changing technological parameters generate outcome variations generally of lower magnitude than the parameter variations introduced. In particular, varying the $\gamma _{i}$ values for non-pecuniary costs has relatively low impacts on results (outcome changes $<<$ parameter variations). Adding or removing new or hypothetical technological options also has a relatively low impact on outcomes. This is a reflection of strong model path-dependence as opposed to parameter dependence. This is further explained in SI Section 3 on model theory.

The converse of this property regards the model’s validity range in time. With a dynamical systems model, one can quantify the time span over which one should expect projections to be valid, with a cone of uncertainty that increases with time from the present day. Given that this model takes part of its parameterisation from recent technological trajectories, the further we model in time, the less reliable projections become. The validity range is determined by the degree of systemic inertia, which in this case is of about 30 years. We discuss the validity range in time of the model in SI Section 6.3, where we explain why a maximum modelling horizon of 2050 is appropriate.

The model also enables a relatively easy method to implement and analyse a large range of policy instruments fairly closely to their actual legal definitions, from regulatory instruments to some types of push and pull strategies. Here, we currently have eight types of policies and used several of them to construct one possible global decarbonisation scenario. It is clear that many other such scenarios can be designed, and assessed alongside one another, each with pros and cons. We note, however, that it is most likely not possible to find an ‘optimal’ policy package when one does not have a representative agent, but has a huge policy space.^26^ Meanwhile, we also find that strong policy interaction arises in the model, through the fact that it is non-linear and based on a diffusion/bandwagon effect theoretical basis. While this complicates policy analysis, we believe that it is closer to reality. A comparison to other model results is given in SI Section 6.2.

We note, however, that we do not achieve the degree of detail of most other IAMs in other important domains, such as infrastructure (Waisman et al. 2013), travel time budgets (Daly et al. 2014), rural/urban splits and range anxiety (McCollum et al. 2016; Pettifor et al. 2017b), other non-pecuniary costs and behavioural features (Pettifor et al. 2017a, b), while we represent modal shift and freight only partially (other studies reviewed in SI Section 2). Furthermore, to model more accurately technological trajectories, we would need to review and include explicitly, for all 59 regions, all transport policies that have been implemented between the start date of the simulation and the present day, a substantial challenge. These are areas that are under development or that can be improved in future work, in a more mature version of the model. We note, however, that including some of these could conflict with our own methods (possible implicit double-counting). For instance, we consider the provision of infrastructure (e.g. for EVs) part of the diffusion process, where for example, kick-start programs imply infrastructure developments. Similarly, rural/urban splits are implicitly accounted for in our distributed cost data; however, they may generate constraints that we do not represent. The difference in model results that some of these would imply are not fully clear to us, for instance where modal shifts reduce the number of road vehicles, or freight electrification, which could reduce emissions substantially.

## Conclusion

Emissions reductions consistent with the 2 ^∘^C target of the Paris Agreement have been extensively demonstrated to be technically feasible (IPCC 2014). However, policy frameworks to reach these goals are not yet clearly established across the world, even where emissions targets are the most stringent. Existing IAMs, rich in technology options, have been used to explore the technically feasible parameter space for decarbonisation. However, the representation of specific policy instruments or realistic portfolios, and their representation of behaviour in agent decision-making in their current use has not been extensive, leaving a gap for advising policy-making.

Here, we presented a model that overcomes many of these issues, with a global transport simulation model that projects the diffusion of innovations based on historical data and choices of heterogenous agents making individual choices, which is part of a global IAM. Instead of optimising a whole system, this model projects its evolution based partly on observed trajectories of technology diffusion, partly on a representation of consumer choices that includes agent heterogeneity, social influence and non-pecuniary aspects. This model type enables a finer representation of specific transport policy instruments that are pecuniary, regulatory or of the technology push type.

We used this model to assess the impacts of a chosen portfolio of transport policies that leads to emissions reductions consistent with a policy target of 2 ^∘^C, and possibly even 1.5 ^∘^C. We find that in such a non-optimisation representation of agent decision-making, policies interact and enable each other. This opens a door to finer model-based analysis of composite transport policy packages, while remaining focused on climate change and global emissions.

We conclude by suggesting that decreasing returns are now emerging with cumulative efforts at mapping the feasible decarbonisation parameter space by modelling optimisations of the transport sector, while demand is increasing for finer detailed impact assessment of possible policy packages. This potentially requires to alter modelling methodologies that are used for analysing climate policy. It also demands to clearly delineate normative analysis, in which one identifies policy objectives, to positive analysis in which the goal is impact assessment of proposed policies, both of which play a different role in the policy cycle. However, this exercise also highlights the limited validity range that decreases in time of any non-prescriptive modelling strategy. In an effective science-policy bridge, IAMs must attempt to assess the impacts of possible composite policy packages that are currently considered by policy-makers. We argue that this is only possible through the use of positive behavioural science and models, and showed that this is possible with a relatively simple non-optimisation modelling framework.

## Electronic supplementary material

Below is the link to the electronic supplementary material.

(PDF 1.66 MB)

(XLSX 12.7 MB)

## Footnotes

1 158 EJ of oil was produced in 2013 and transformed into many products of which 42 EJ was gasoline and 51 EJ was diesel, of which 34 EJ and 42 EJ were used in road transportation.

2 Cost-optimisation models include PRIMES (E3MLab 2015), MESSAGE (IIASA 2013), REMIND (PIK 2011), AIM-end-use (NIES 2012), TIMES (IEA-ETSAP 2016b), TIAM (UKERC 2011), GET (Grahn et al. 2013); utility maximisation (general equilibrium) models include GEM-E3 (E3MLab 2013), IMACLIM (CIRED 2006), GEMINI (EPFL 2008); bottom-up technology models based on discrete choice theory include IMAGE/TIMER (Bouwman et al. 2006), IMACLIM (CIRED 2006), CIMS (Rivers and Jaccard 2006). Simpler IAMs such as DICE (Nordhaus 2013) and FUND (Anthoff and Tol 2014) are also optimisations.

3 In other words, if agents behave following their peers to any degree, the aggregate macro-behaviour of a group of cost/utility-optimising agents does not result in an optimum at the system level (and conversely), since that optimal point ceases to exist.

4 We acknowledge that heterogeneity in principle includes varying characteristics across adopter groups, which also means that receptivity to social influence is itself heterogenous (see e.g. Donnelly and Ivancevich 1974). This is, however too detailed for the present study.

5 Computable general equilibrium (CGE) and cost-optimisation (partial equilibrium).

6 Many partial equilibrium (cost-optimisation) models can be described as ‘moving equilibrium’ models (Young 2009), in which diffusion is driven by appropriately chosen external parameters producing the right profiles.

7 These are also common concepts in marketing research carried out by firms placing products, going back to Smith (1956); Bass (1969).

8 See for instance Aini et al. (2013); Baltas and Saridakis (2013).

9 E.g. in Mercure and Lam (2015), we reviewed the characteristics of over 8000 different individual vehicle models in registration data for the UK, from DVLA (2012a), and we are fully convinced from that experience that consumers in the UK do not carry out such an exhaustive search when choosing a vehicle.

10 Meaning that shares are not single valued functions of perceived costs, they also depend on configurational history: many sets of $S_{i}$ can occur with each set of $C_{i}$, depending on what $S_{i}$ and $C_{i}$ have been in the past.

11 By momentum we mean that the system has some degree of inertia that prevents it from changing direction very rapidly.

12 This can be expressed in a myriad of ways, e.g. from the network structure of technology evolution (Grübler 1998), from an evolutionary perspective (Young 2001, 2009; Hofbauer and Sigmund 1998), from the scaling dynamics of innovation (Arthur and Polak 2006), or from the presence of multi-agent interactions (Mercure 2018).

13 In dollar per person-kilometre ($/pkm).

14 Every term is distributed; however the distribution of car prices dominates variations.

15 We show in Mercure and Lam (2015) that engine sizes strongly relate to prices, hence this classification.

16 Due to lack of detailed or reliable shares data worldwide, plug-in hybrids are not represented explicitly but are instead lumped together with EVs. Due to lack of shares and cost data, fuel cell vehicles are not currently included but may be included in the future as a dominant design forms and reliable costs can be obtained.

17 These regressions did not include variables such as the extent of road network, congestion, urban vs rural population ratios, omitted due to the difficulty of obtaining such data consistently for 59 E3ME regions worldwide. Note that these variables change only slowly over time and therefore would not significantly improve the reliability of our parameters.

18 FTT calculates vintage effects due to the age of vehicles and the fact that fuel efficiencies were lower in the past, based on evidence from our UK dataset (DVLA 2012b).

19 A detailed review of all existing policies in 59 regions, representing a substantial challenge, is in progress but has not yet been completed nor integrated to our baseline, and it is clear that very recent policies not explicitly included here could affect our projected technological trajectories. This doesn’t affect the validity of our methodology.

20 Consumer discount rates in vehicle purchases are controversial (Busse et al. 2013; OECD 2010) and could lie anywhere between 5% and 40%. However, the fact that some studies identify high discount rates signals that some consumers take relatively little consideration of future fuel savings when purchasing a vehicle. At 15%, the incentive of a fuel tax, per unit of carbon taxed, is comparatively much smaller than that for a tax at registration time (e.g. see the supplementary information in Mercure and Lam 2015).

21 In the model, in many regions, small and mid-size EVs have zero market shares (zero sales in 2012, e.g. in India, China, Brazil), and thus, policy step 3 involved exogenously introducing non-zero shares. Our assumption is that in 2020, 0.01% shares are purchased by governments to kick-start the EV markets. We did not include infrastructure costs.

22 For reference, around 50 EJ of bioenergy is currently used globally for traditional heating and cooking (IEA 2015b).

23 Note furthermore that total use of biofuels declines post-2050 due to increasing diffusion of electric vehicles and the gradual phase out of ICE engines. We do not include in the transport sector land-use change emissions. Land-use modelling is required to estimate what an additional 27 EJ of biofuel production means for land-use change and agriculture emissions.

24 Total electricity demand was of order 80 EJ in 2016 and may increase to between 130 EJ (2^∘^C) and 170 EJ (baseline) in 2050, according to E3ME; more electricity is saved through decarbonisation than what is demanded by transport. We do not consider changes in intermittency of power demand due to EVs, which could be substantial.

25 Due to relative combustion efficiencies between power plants and internal combustion engines, fleet electrification reduces emissions even if the power sector is coal intensive. Thus we do not quote full life-cycle emissions from transport. Baseline power generation emissions from FTT:Power in our model increase by 1% when decarbonising transport alone, while total emissions go down by 8%. However we do not consider likely that stringent decarbonisation policies would be adopted for transport but not for power generation.

26 Optimising all possible scenarios that can be generated by FTT, with its huge policy parameter space, appears challenging with our computing power, simply due to its degree of non-linearity, which we argue is quite representative of the real world.
