# Supplementary material for: Integrated assessment modelling as a positive science: private passenger road transport policies to meet a climate target well below 2 ∘C
Source: Clim Change. 2018 Sep 3;151(2):109–29. doi: 10.1007/s10584-018-2262-7 (PMC6404732; doi:10.1007/s10584-018-2262-7)
Supplement: Supplementary file 1 — (PDF 1.66 MB) [file 10584_2018_2262_MOESM1_ESM.pdf]

FTT:Transport - Detailed model description and data gathering procedure

Jean-François Mercure[a,c,*], Aileen Lam[b,1]

[a]*Department of Environmental Science, Radboud University, PO Box 9010, 6500 GL Nijmegen, The Netherlands*
[b]*Department of Economics, Faculty of Social Sciences, Humanities and Social Science Building, University of Macao, E21, Avenida da Universidade, Taipa, Macao, China*
[c]*Cambridge Centre for Environment, Energy and Natural Resource Governance (C-EENRG), University of Cambridge, 19 Silver Street, Cambridge, CB3 1EP, United Kingdom*

Contents

1 Introduction 3

2 Review of the modelling context 4
- 2.1 Energy system models . . . 4
  - 2.1.1 Energy sector optimisation models . . . 4
  - 2.1.2 Macroeconomic models . . . 5
  - 2.1.3 Simulation models . . . 6
- 2.2 Modelling technology transitions . . . 7
- 2.3 Technological transition in the transport sector . . . 7
  - 2.3.1 S-shaped curves of technology diffusion . . . 8
  - 2.3.2 Path dependence . . . 9
- 2.4 Choice modelling . . . 9
  - 2.4.1 Choice heterogeneity . . . 9
- 2.5 Social influence and consumer choices . . . 10
  - 2.5.1 Modelling choice with intangibles . . . 11
- 2.6 Policy assessment for emissions from the transport sector . . . 11

3 Detailed theoretical model description for FTT:Transport 13
- 3.1 Basic equations . . . 13
  - 3.1.1 Transport demand . . . 13
  - 3.1.2 Fuel use and emissions . . . 14
  - 3.1.3 Investment, learning and cost reductions . . . 15
- 3.2 The decision-making model core . . . 15
  - 3.2.1 Perceived costs and decision-making . . . 15
  - 3.2.2 Diversity is crucial . . . 16
  - 3.2.3 Pairwise choices comparisons of distributed choices . . . 17
  - 3.2.4 Relationship to discrete choice modelling and the binary logit model . . . 18
  - 3.2.5 Correspondence between consumer diversity and elasticities of substitution . . . 19
  - 3.2.6 The levelised cost of transportation . . . 19
  - 3.2.7 Using log-normal distributions . . . 20
  - 3.2.8 The generalised cost as a comparison measure . . . 21
- 3.3 Population changes as a result of decision-making . . . 21

*Corresponding author: Jean-François Mercure
Email address: `J.Mercure@science.ru.nl` (Jean-François Mercure)

Preprint submitted to Climatic Change *June 28, 2018*

3.3.1 Survival rates and technology vintages . . . 21
3.3.2 Application: emissions factors of the fleet vs new vehicles . . . 22
3.3.3 Population dynamics . . . 23
3.3.4 The frequency of decision-making . . . 25
3.3.5 A theory of social influence in vehicle purchases . . . 25
3.3.6 Coherence length in FTT . . . 27
3.4 Integration to E3ME . . . 27
3.5 Policy in FTT:Transport . . . 28
3.5.1 Pecuniary incentives . . . 28
3.5.2 Regulatory and push policies . . . 29
3.5.3 Corresponding real-world policies . . . 29

4 Deriving general information from the UK fleet **30**
4.1 Procedure and datasets . . . 30
4.2 Distributions and heterogeneity . . . 31
4.2.1 Overall new car price distribution . . . 31
4.2.2 Price and emissions distributions of new vehicles by category . . . 31
4.2.3 Distribution of emissions, engine size and vehicle mass for the current fleet . . . 38
4.2.4 Distributions by size class for the current fleet . . . 38
4.3 Vehicle lifetimes and survival . . . 38
4.3.1 Age distribution of vehicles by category and survival functions . . . 38
4.3.2 Survival analysis . . . 38
4.3.3 Emissions factors changing over time . . . 41
4.4 Motorcycles . . . 42
4.4.1 Motorcycle age distributions and survival functions . . . 42

5 Data parameterisation of FTT:Transport **45**
5.1 Cross-sectional data gathering procedure and distributions for 18 regions and proxies . . . 45
5.2 Construction of historical time-series . . . 46
5.2.1 Market shares time-series . . . 46
5.2.2 Survival function . . . 50
5.3 General parameters . . . 52
5.4 Econometrics for transport demand and vehicle sales . . . 52
5.5 Determining the non-pecuniary γ values in practice . . . 57

6 Sensitivity analyses and model validity **59**
6.1 Sensitivity of technological parameters . . . 60
6.2 Comparison to other modelling approaches . . . 65
6.3 Validity range of the model . . . 66

A The addition of probabilistically distributed values **69**
A.1 The addition is a convolution . . . 69
A.2 Case for normal distributions: standard error analysis . . . 69
A.3 Error propagation . . . 70

B List of variables **71**

1. Introduction

FTT:Transport, in its 2016 version, is a global model of technological change in road transport. It is based, conceptually, in parts on previous work for the power sector, FTT:Power (Mercure, 2012), using the same evolutionary economics approach and the replicator dynamics equation. Vehicle choice for passenger transport, however, is much more complex to model, in our view, than technology choice in industry. This is simply because the reasons coming into play in consumer choices are more heterogenous and varied than in firms. For example, some vehicles sell at $15,000, some vehicles sell at $150,000, while both apparently supply the same mobility services. However, just as in the clothing industry, extensive sociological studies exist, and are continuously carried out, to better understand how this choice is made. Here we present the details of the FTT:Transport model, which attempts to model these choices in a way detailed enough to produce useful insight for transport policy design with the purpose of reducing road transport emissions.

FTT:Transport is dynamically integrated to the E3ME macroeconometric model, in the FORTRAN language, maintained and operated by Cambridge Econometrics Ltd (see `www.e3me.com`), but also implemented in a separate stand-alone version as an opensource MATLAB code and graphical user interface, that can be obtained by contacting the authors. When a model exists in two different implementations, it is a challenge to maintain both such that they continually produce identical results. While the authors make extensive efforts at maintaining the two models identical, this cannot be guaranteed. The version as integrated into E3ME features dynamical feedbacks with the global economic model, and is used more frequently, thus considered the official version. The matlab version, however, benefits from an easy-to-use graphical user interface and is opensource. The overall simulation-based integrated assessment model E3ME-FTT-GENIE1 is described in Mercure et al. (2017).

2. Review of the modelling context

This section presents a literature review of all the main aspects surrounding our research objective and identifies knowledge gaps that lead to our research. In our climate change mitigation perspective, transport modelling is seen through the lens of energy systems, since we are focusing on modelling transport emissions as opposed to many other aspects of transport systems. In this context transport will often be modelled within larger energy system models. Studying transport emissions however involves changing technologies, which thus invokes technology transitions theory in order to understand the process of diffusion. But technological change takes place only if decisions to change occur, and this requires understanding consumer choices, for which we bring in discrete choice theory. Thus this section is subdivided into three parts: a review on the existing energy system models, a review on the technology transitions literature and a review on choice modelling for energy system models.

2.1. *Energy system models*

Energy system models are valuable mathematical tools that have been used to help understand how to plan, operate and coordinate complex networks of energy supply and demand technologies. With the issue of climate change taking increasing prominence within policy discussions, energy models have taken a central for advising climate policy-making with the goal of reducing GHG emissions. Transport producing a significant fraction of global emissions, the generation of mobility services has often been included in energy models. Since studying climate change requires knowledge of the global amount of GHGs emitted, models used for climate policy are often global models. This is the type of models reviewed here.

A variety of modelling techniques have been utilised for studying energy and emission projections. They vary in terms of data requirements, technology specifications and computing demands. Following several existing studies (see e.g. Grubb et al. 2002, Herbst et al. 2012, Löschel 2002, Nakata et al. 2011), we divide energy system models into five main non-exclusive categories, according their underlying methodologies. This is summarised in Table 2.1.1.

2.1.1. *Energy sector optimisation models*

Energy-oriented models are designed to consider the energy sector and emissions from energy production and consumption in detail. One of the most adopted approaches in modelling the energy sector is optimisation. The most well known examples that utilise the optimisation approach include MARKAL and MESSAGE. Both have been employed extensively to model transport and energy systems (Gül et al., 2009, McCollum et al., 2013, Yeh and McCollum, 2011). MARKAL is a linear programming optimisation model developed by the Energy Technology Systems Analysis (ETSAP) of the IEA (IEA, 2012), which has been adapted into numerous variants across the globe. Related to MARKAL, the TIMES Model (**T**he **I**ntegrated **M**ARKAL-**E**FOM **S**ystem) is a bottom-up technology rich optimisation model generator (EC, 2008). MESSAGE is is a dynamic linear programming model that calculates cost minimal supply structures over a given time horizon (see Messner, 1995). It provides core inputs for major international assessment and scenario studies, such as the Intergovernmental Panel of Climate Change (IPCC), the World Energy Council (WEC) and the Global Energy Assessment (GEA IIASA, 2010). Other energy sector models that operate with an optimisation framework include LEAP (Heaps, 2012), REDGEM70 (Takeshita and Yamaji, 2008), MERGE (Kypreos, 2005), REMIND (Leimbach et al., 2010), BEAP (Grahn et al., 2007), GET (Grahn et al., 2007), OSEMOSYS (Moksnes et al., 2015) and PRIMES (Capros et al., 2009).

The strength of optimisation frameworks is their ability to find cost minimal energy pathways, as normative planning tools. They feature however potential convergence problems due to non-convexities when including learning curves, which can lead to multiple solutions that then need to be sorted (Köhler et al., 2006). The traditional optimisation model assume a social planner with perfect foresight, where ideal scenarios are sought that achieve particular goals, which are 'internalised' to the optimisation (e.g. energy security, climate change, health and other externalities). However, for a purely descriptive purpose, information for the full time frame is not in reality available immediately to agents responsible for energy sector developments, making perfect foresight inconsistent with positive modelling goals (Anderson et al., 2011, Greene, 2010). To this end, the new myopic (or limited foresight) modelling approach incorporated as an option in some optimisation frameworks, such as MESSAGE and GET-LFL, allows for the analysis using different time horizons for decision making (see Hedenus et al. 2005, IIASA 2003, Nyqvist 2005). While the myopic approach improves upon traditional perfect foresight approach by providing a framework for exploring some degree of 'path dependency' in the energy system (see below), it has been argued that *bounded rationality* (Simon,

Model name	Type	Methodology/tools	ETC	Global transport
MARKAL, TIMES, GET, MESSAGE, REDGEM70, BEAP, REMIND, LEAP	Energy sector models	Linear optimisation	Yes	Yes
PRIMES	Energy sector model	Non-linear optimisation	Yes	Yes
TREMOVE, WEM	Simulation model	Simulation and optimisation	Yes	Yes
IMAGE/TIMER, ASTRA, CIMS, GLADSTE	Simulation model	System dynamics and non-optimisation	Yes	Yes
POLES	Simulation model	Simulation and partial equilibrium	Yes	Yes
TAPAS	Simulation model	Agent-based model	Yes	Yes
MESSAGE-MACRO, MARKAL-MACRO, RICE, DICE, GEM-E3	Macroeconomic model	Optimisation framework	Yes	No
E3MG	Macroeconomic model	Non-optimising dynamic simulation approach	Yes	No
MERGE, FUND, CETA, WIAGEM	IAM	Optimisation hybrid models	Yes	yes
CIMS, GREEN, NEMS, GEM-E3, WITCH	CGE	Equilibrium structure	No	yes

Table 1: Examples some major energy models. *ETC* stands for endogenous technical change. *Global transport* indicates whether models have a component to model the global transportation system.

1972) embedded in decision making involves factors not considered in these approaches, such as adaptive behaviour or network (bandwagon) effects, which we discuss below.

2.1.2. Macroeconomic models

Macroeconomic models focus on the entire economy of a society, considering the energy sector as one subcomponent (Nakata et al., 2011). Macroeconomic models are usually top-down models that have higher sectoral aggregation and better characterisation of impacts of economic growth (Hourcade, 1993). Although they reflect greater details regarding macroeconomic feedbacks, they provide a less detailed description of technology and technological change.

Most macroeconomic models use the Computable General Equilibrium (CGE) approach. This includes GEM-E3 (**G**eneral **E**quilibrium **M**odel for **E**nergy-**E**conomy-**E**nvironment interactions, Capros et al. 1997) and NEMS (**N**ational **E**nergy **M**odelling **S**ystem, EIA 2010). CGE models have been criticised for a general lack of detailed technological information regarding the energy system (Böhringer, 1998). In particular, CGEs face considerable difficulties in incorporating Endogenous Technological Change (ETC). This is because linear programming is generally more suited to solving problems with a single maximum, and the introduction of a increasing returns to scale associated to ETC can generate several equilibria (Köhler et al., 2006), a consequence of the path-dependent nature of ETC and the accumulation of knowledge. Considerable improvement has been made regarding the incorporation of ETC into equilibrium models Edenhofer et al. (2006), which however involves complex searching methods for finding the 'true' system equilibrium.

Traditional macroeconomic models typically assume that there exists an autonomous energy efficiency improve-

ment (AEEI), and, thus, technological change is exogenous to the model (Grubb et al., 2002). The exogenous technical growth assumption with AEEI has been criticised for neglecting the interactions between policy options and technological change (see Gillingham et al. 2008, Köhler et al. 2006, Weyant and Olavson 1999). This can significantly bias the policy assessment because policies induce technical change (ITC) and cost reduction within the system (Goulder and Schneider, 1999, Köhler et al., 2006, van der Zwaan et al., 2002).

In response to criticisms on modelling technological change, hybrid models were that incorporate both a detailed energy component (bottom up) and a neoclassical optimal growth economic structure (top-down). This includes CIMS (**C**anadian **I**ntegrated **M**odelling **S**ystem, Navius 2010), GREEN (**G**eneral **E**quilibrium **E**nvironmental Model, Burniaux et al. 1992) and WITCH (**W**orld **I**nduced **T**echnical **C**hange **H**ybrid Bosetti et al. 2006). Hybrid models bridge the gap between conventional top-down and bottom-up modelling approaches (Hourcade et al., 2006). Other hybrid models include MESSAGE-MACRO and MARKAL-MACRO, MERGE (Kypreos, 2005, Kypreos and Bahn, 2003) and REMIND-D (Leimbach et al., 2010). Of particular interest here, the E3ME-FTT model is a hybrid non-equilibrium macroeconometric model based upon a Post Keynesian demand-led economic view (Barker et al., 2012, 2006, 2008), with bottom-up non-optimisation models of technology (FTT, see Mercure 2012, Mercure et al. 2014; see Mercure et al. 2017 for a full description of E3ME-FTT).

2.1.3. Simulation models

Quantitative simulation models can provide important insights about the effects of energy policies because they allow for the assessment of consequences of policy and economic measures and a straightforward incorporation of increasing returns and path-dependence (see below), which contrasts with optimisation models. Well known examples of simulation models include the World Energy Model (WEM) (IEA, 2007), POLES (Criqui et al., 1999) and TREMOVE for the transport sector in the EU (Van Herbruggen and Logghe, 2005). This section discusses two major approaches in simulation modelling: systems dynamics and agent-based.

Systems dynamics approach (SD):

SD is ‘the study of information-feedback characteristics of industrial activity to show how organisational structure, amplification and time delays interact to influence the success of enterprise’ (Forrester, 1961). It combines technology and market-behaviour frameworks into one in order to represent the causal structure of the system (Martinsen and Krey, 2008). It is used to analyse the wider impacts of policies being tested (Fernandez and Xavier, 2013) which, in terms of methodology, contrasts with the search for optimal scenarios done with optimisation models [1]. While SD offers clear benefits to modelling energy systems characterised by a large number of interactions between several variables (Armenia et al., 2010), it is not widely applied to energy system models. In this class, IMAGE/TIMER (The **T**argets **IM**age **E**nervy **R**egional, De Vries et al. 2002) analyses the long-term dynamics of the energy system. With a focus on transport, both ASTRA (**AS**sessment of **TRA**nsport Strategies, Fiorello et al. 2010) and GLADYSTE (Global Scale System Dynamic Simulation Model for Transport, FERMI et al. 2010) are system dynamics models at a European scale for the strategic assessment of policy scenarios. Technology vintage models also fall within this class since they track technology units from their construction to their dismissal, which includes CIMS (see above).

We note however that any simulation models based on Multinomial Logits (MNL) or constant elasticity of substitution (CES) functions use the assumption of perfect information and no interactions between agents, but instead, have a representative agent. The MNL can be converted into a CES using a basic optimisation calculation. This means that they have a theoretical basis closely related to optimisation in general (for instance, CGE models, even without foresight in recursive dynamic mode, remain utility-optimisation models).

Within its own class which also involves systematic feedbacks, amplification and time delays, the FTT family of models, which includes FTT:Transport, can be seen as SD models with a technology vintage component. However, unlike many SD models that use the Vensim system with multiple nodes, the Lotka-Volterra approach of the FTT family of models naturally includes more dynamics and interactions not represented in traditional SD models, while it assumes bounded rationality and multi-agent interactions (which implies that it rejects the notion of a representative agent).

[1] Unless one accepts a debatable premise that energy systems are perpetually optimal, a subject that requires a separate discussion. See Mercure et al. (2014).

Agent-based modelling approach (ABM)

Social science often involves and emphasises heterogenous human agents with diverse preferences, making diverse choices. ABM is a computerised simulation of a number of interacting decision-makers. Each assesses its situation and makes decisions on the basis of a set of rules (Bonabeau, 2002). Since ABMs simulate learning at the individual level and in the model of innovation networks (Gilbert, 2008), it is a way to model the system dynamics and complex adaptive properties.[2]

ABMs have clear advantages in modelling consumer decisions and agent diversity. They have been applied to modelling technology adoption in transport systems. For example, TAPAS (**T**ransportation **A**nd **P**roduction **A**gent-based **S**imulator, Holmgren et al. 2012) is a micro-level model for the assessment of different types of transport-related policies. Eppstein et al. (2011) has developed an ABM of vehicle consumers that incorporates spatial and social effects. Köhler et al. (2009) used ABM to produce a representation of the Multi-Level Perspective on technology transitions, which features strongly non-linear effects with a representation of technology diffusion consistent with observed S-shaped diffusion patterns, described below.

Unlike other global energy system models, the ABMs comprise primary research models applied to particular study areas, or to restricted geographical scope (Wegener, 2004). This is because complete input data for ABM above national levels is difficult. Furthermore simulating the behaviour of large numbers of agents can also be computationally intensive (Bonabeau, 2002). Thus, detailed ABMs are less suitable for modelling transport emissions at the global scale required for climate change-related research.

2.2. *Modelling technology transitions*

Within many of the approaches and models listed above for modelling GHG emissions remains a general lack of an *endogenous* representation of technological change. Within optimisation models, technological change takes place in such a way that given changes of policy context, the energy system remains at a cost minimum. The rate of diffusion is limited exogenously however, constraining the results of the optimisation. These diffusion rates are are therefore among the most critical assumptions for assessments of long term emissions and energy issues. In optimisation models, diffusion rates were found to be possibly pessimistic in comparison with observed patterns of diffusion Wilson et al. (2013). Beyond overcoming pessimism, added realism would require that, as empirical work tells us, diffusion should be represented as context and time dependent, and this effectively opens Pandora's box.

Missing in many models is a representation of how technologies come to gradually appear and diffuse to widespread use, which is not instantaneous and involves building up new markets and new production capacity for new technologies. Technology diffusion is known in textbooks to follow S-shaped curves, which is supported by a large body of literature. Ideally, added realism in technology models would include increasing amounts of elements found there. We thus review here the empirical and theoretical literature on technology diffusion and transitions theory.

2.3. *Technological transition in the transport sector*

The socio-technical system describes systems that involve complex interactions between human, machine and environment (Baxter and Sommerville, 2011). A transition of socio-technical regime (STR) is a set of processes that lead to a fundamental shift in the socio-technical regime (Geels and Schot, 2007, Kemp, 1994). STR involves technological changes, user practices, regulations, and industrial networks, with a range of actors and over a period of time (Geels, 2005).

The modern automobile industry is deeply embedded into legal, social, cultural and economic practices. The lock-in mechanisms imply that socio-technical regimes are extremely rigid in the automobile market. There are substantial sunk investments in plants for IC engines, skills and fuel infrastructure. Personal mobility practices have also become dominated by petrol-based cars, in turn shaping urban infrastructure. The majority of cars on the street are internal combustion engines and there are strong path dependencies on automobile consumption (Geels et al., 2011). Consumers do not only optimise cost when they choose a car, instead, consumers take into account the social trend, the availability of infrastructure and the models available in the market that match their preferences when they purchase a car (Tanaka et al., 2014).

[2]A complex adaptive system is defined as being composed of population of adaptive agents whose interactions result in complex non-linear dynamics (Brownlee et al., 2007).

2.3.1. S-shaped curves of technology diffusion

Technology diffusion is often described by S-shaped sigmoid curves. S-curves illustrate the fact that technology diffusion is a gradual process, with a slow initial growth rate, followed by accelerated growth as as new markets are reached, and slowing down again (Barreto and Kemp, 2008). One of the earliest classic studies was conducted by Griliches, who found that the penetration of corn seeds followed logistic curves (Griliches, 1957). Mansfield (1961) described how the diffusion innovations follows a behaviour similar to the spread of an epidemic.

Fisher and Pry (1972) subsequently also proposed a 'technological substitution model', which describes the penetration process of new technologies replacing old ones with S-shape curves. Marchetti et al. (1980) expanded the Fisher-Pry model into a model involving more than two technologies, while Nakicenovic (1986) explored specifically the early 20th century transition in transport. Empirically, analysis found that diffusion and substitution of transport technologies historically followed S-shape curves (see e.g. Grubler 1990, Wilson et al. 2012 for further reviews and analysis).

The logistic diffusion process is consistent with long wave theory (Freeman and Louçã, 2002), a socio-technical approach to transitions (Kemp et al., 2001) and evolutionary economics (Winter and Nelson, 1982) that highlight co-evolution and multi-dimensional interactions between technology and society. In particular, Metcalfe (2004) argues how Mansfield's work can be interpreted as an expression of evolutionary economic behaviour. Geels (2002) and Elzen et al. (2004) propose adopting a dynamic multi-level perspective on system innovations. The process in the multi-level perspective consists of four phases. The first two represent the emergence of technology niches and the development of technical trajectory through 'probing and learning'. The third describes the wider diffusion of the technology and in the four, how the new technology replaces the old technology.

In technology diffusion models, it is common to represent technology diffusion with an S-curve. Dominant models of explanation of this pattern have been epidemic and neoclassical models of diffusion (Nill, 2008). Diffusion in epidemic models is determined by the 'epidemic spread of information among potential adopters'. Thus, adoption is a function of the product of the uninfected numbers and the share of population that is already infected (Sarkar, 1998). While this approach picks up information contagions of users, it is often criticised by economists as having neglected the economic aspects of diffusion (Geroski, 2000). Neoclassical models base their explanation of diffusion on heterogenous adopters. While they provide useful insights into the differences between potential adopters, criticisms are mainly directed at the assumption of the equilibrium approach (Sarkar, 1998). In essence, both epidemic and neoclassical models might have missed important features of technological evolution, such as the dynamics of technological competition (Nill and Kemp, 2009) and complex systems driven by co-evolutionary interactions (Garnsey and McGlade, 2006).

The evolution paradigm of technological change has its root in Schumpeter's theory (Schumpeter, 1942), which analyses innovation as a historical process and technological substitution as a process of 'creative destruction' of prior technologies (Levinthal, 1998, Schumpeter, 1942). To use an analogy with biology, when an invasive species proves to posses better 'fitness' to environmental conditions, this species may come dominate at the expense of others by expanding in ecological space. Similarly, the growth of any technology depends on its fitness to markets in comparison to competitors. If the parallel with population ecology holds, then diffusion should depend on population sizes as well as fitness and follow standard population dynamics.

A number of studies have shown that Lotka-Volterra population competition equations (LVC), a set of coupled differential equations, can be applied to model technological diffusion (e.g. Bhargava, 1989, Grubler, 1990, Marchetti et al., 1980, Morris and Pratt, 2003, Saviotti and Mani, 1995) and organisational change (Lee et al., 2009). Equivalent to the replicator dynamics equations commonly used in evolutionary economics and evolutionary game theory (Hofbauer and Sigmund, 1998, Safarzynska and van den Bergh, 2010), the LVC effectively represents the growth and decline of technologies competing in a market according to the size of their industries and their fitness. In his argument bridging diffusion work to evolutionary economics, Metcalfe (2004) suggests that S-curves, corresponding to the two-technology case, are only a subset of possible profiles of diffusion, themselves governed by a more general law, the replicator dynamics or equivalently the LVC. In other words, in a problem where three or more units interact, diffusion does not follow simple S-curves but instead is described by the LVC.

Given that, in all previous work, to our knowledge, no clear method has been suggested for parameterising an LVC system. The problem is obvious: in a two technology case, because it is a non-linear system, one requires diffusion data up to beyond the inflexion point of the logistic curve in order to be able to determine the diffusion timescale

(the parameter of the logistic function). This means that it can only be determined for technologies that have already diffused past half of the total market. However, to be useful for forecasting technology diffusion, it is in the cases where diffusion is

2.3.2. *Path dependence*

The argument that technological change is path dependent was advanced by Arthur et al. (1987), Arthur (1989, 1994) and through the work David (1985, 2007) conducted on the economic history of technology. Path dependence arises when some economic processes have increasing returns to scale, or, say, technology adoption has increasing returns to adoption (i.e. positive feedbacks). As Arthur demonstrates, in the presence of increasing returns to adoption, technology lock-ins can arise as a result of small 'historical accidents', which may seem to make little economic sense from a rational perspective.

Path dependence in energy systems arises, when positive feedbacks are present, from differences in initial conditions, engineering traditions, policy choices and historical accidents leading to differences in infrastructures and consumption patterns (Grubler and Cleveland, 2008). Four types of processes will lead to path dependent behaviour: increasing financial returns to adoption (learning curves), and coordination benefits, the latter arising when technical constraints increase the usefulness of a technology the more it is adopted. Technology investments are also highly irreversible, with decision rules chained to the context (Saviotti and Metcalfe, 1991). Within the automotive industry, while visions of what future mobility could be exist, agents in the system can remain very stubborn facing change (Howey et al., 2010, Wells and Nieuwenhuis, 2012, Wells et al., 2010), for reasons that the modeller, or the theorist, cannot know or exhaustively enumerate. However, as is known historically, after the mobility transition of the early 20th century, once economies and societies were 'locked in' to petrol vehicles, then huge increasing returns were seen from petrol car production and infrastructure. Social life also became irreversibly locked into that mode of mobility (Urry, 2004). Thus from this historical perspective, it is difficult to justify theoretical structures that lack path dependence, despite that it implies a higher level of complexity: several or no equilibrium points, uncertainty propagation and the importance of small events.

Recognising the importance of path dependence, technological change modelling has gone through major improvements, especially in the field of climate policy, with Endogenous Technical Change (ETC) incorporated (Edenhofer et al., 2006). There are two main concepts employed: knowledge capital at the sectoral scale and learning curves at the technology level (Köhler et al., 2006). Essentially, knowledge can be generated through investment in R&D, while with learning-by-doing, the costs of specific technologies decrease with experience and cumulative investment. These however are only two of the possible ways in which path dependence arises, those associated to prices changing with adoption. Possible coordination benefits unrelated to prices, for example, are missing in these models.

Using the replicator dynamics, the FTT family of technology models has a natural representation of path dependence that stems from feedback structures and self-reinforces the dynamic system of equations. It describes the interactions between the populations of organisation and captures theories (including irreversibility, hysteresis and non-linearity) associated to path dependence (Ebeling et al., 2001). With cumulative causation endemic in technology adoption decisions represented in by the replicator equation (Carrillo-Hermosilla and Unruh, 2006, Jacobsson and Bergek, 2004), and economic factors considered by a dynamic simulation model of the economy (E3ME), FTT enables to simulate energy systems in the presence of strong path dependence.

2.4. *Choice modelling*

2.4.1. *Choice heterogeneity*

While industrial dynamics accounts for technology diffusion capabilities, consumer demand drives the rate and direction of innovation (Nemet, 2009). The adoption rate is related to the perceived benefits received by the user and the costs of adoption (Hall and Khan, 2003). Even though 'non economic factors', such as tastes and needs, are unique to heterogenous consumers, traditional technology models (e.g. MESSAGE, LEAP) often approximate the system to representative agents and assume a homogenous society with perfectly coordinated choices that keep technology systems at overall cost minima (Jebaraj and Iniyan, 2006).

More recently, some energy system models (e.g TIMER, CIMS, GLADYSTE, TREMOVE, IMACLIM) have integrated consumer heterogeneity with discrete choice models. Discrete choice theory is a prolific field of economics that enable to parameterise models that predict the choices of heterogenous groups of people using information either

from surveys, from socioeconomic data or from revealed preferences (see for instance Ben-Akiva and Lerman, 1985a). With such a parameterisation, a discrete choice model can predict how the choices of a group of people is likely to change given a change of context (e.g. prices, value, income, distance, etc), summarised by the MNL (and equivalently CES). This has been used extensively to model transport mode choice in cities using surveys carried out over, for example, a population of commuters, situations in which all agents have the same knowledge and do not influence each other. However, while parameterising a discrete choice model using surveys at the country scale is quite challenging, it is simply impossible at the global scale. This work proposes a method to parameterise a discrete choice model based on market revealed preferences, which gives a reasonable approximation to parameters without using surveys. But furthermore, the assumption of agents with the same knowledge clearly breaks down at a national scale and when modelling the whole transport fleet. Clearly, agents do not analyse the whole vehicle market (which features thousands of model variants), but instead clearly influence each other. In this case, an assumption of bounded rationality with social interactions seems unavoidable, and thus we argue that the MNL is not suitable.

'Diversity' is an attribute of any system whose elements may be apportioned into categories (Leonard and Jones, 1989). According to Stirling (1994, 2007), diversity concepts display some combinations of three basic properties, namely, 'variety', 'balance' and 'disparity'.

Variety is the number of variants to which system elements are apportioned. For this work, vehicles technologies are represented according to the distribution of revealed-preference data to people's actual choices. Balance is a function of the pattern of apportionment of elements across categories. Referred to as evenness and concentration, balance is analogous to statistical variance (Pielou, 1977). Thus, instead of utilising a mean cost comparison (Grahn et al., 2009, Schaefer and Jacoby, 2005), this work uses a probabilistic treatment with variance reflecting of heterogeneity of products (see Mercure 2012). Disparity refers to the manner and degree in which the elements may be distinguished. It is implicitly implied in the representations used to characterise variety and balance (Stirling, 2007). The degree of disparity between categories of products (or technologies) determines cross elasticities and the degree of substitutability between products and, thus, is consistent with discrete choice theory.

Product diversity can be interpreted as different consumers using different varieties, or as diversification on the part of each consumer (Dixit and Stiglitz, 1977). The basic premise is that product variety reflects the requirements of market segments (Adner and Levinthal, 2001, Horrace et al., 2009, Smith, 1956). Product differentiation is concerned with bending the will of demand to the will of supply (Smith, 1956). Thus, the diversity in products, represented by cost distribution in FTT: Transport, reflects choices diversity.

Evolutionary thinking in technology has long argued that competitive selection can only operate if there is sufficient economic diversity of behaviour (Metcalfe and Miles, 1994, Turner, 1992). The reasons are two-fold. Firstly, the difference between consumer choices drives competition between varieties, leading to a selection process that determines the rate of technological change (Basalla, 1988, Saviotti and Mani, 1995). Secondly, the basic premise of the adoption and diffusion of technology is that there are different categories of adopters (Rogers, 2010, Rogers and Kim, 1985, Slater and Mohr, 2006). Diversity of choices is therefore crucial in determining the rate of technology diffusion.

2.5. *Social influence and consumer choices*

Social influences and consumer choices are consistently found to be important in energy modelling. Within the transport sector, behaviour adaptation, network interactions and diversity of consumer preferences are central to the understanding of technology adoption (McShane et al., 2012, Mueller and de Haan, 2009). The rate of technological update is influenced by the diversity of the agent's perception towards car purchases. The difference between consumer choices drives competition between product varieties, leading to a selection process that determines the rate of technological change (Basalla, 1988). Based on Rogers (2010), diversity is responsible for the gradual adoption of innovations and technology diffusion.

Within the transport sector, consumer choices for certain vehicle technologies take place within contexts of distributed income that span several orders of magnitude (Mercure and Lam, 2015a). The fact that consumers are diverse implies that car technologies will not diffuse into the market instantly in the presence of a change in price or policy reform. Instead, car technologies diffuse gradually according to consumers' choices and the heterogeneity within the population.

General equilibrium and partial equilibrium (optimisation) models do not sufficiently account for agent diversity (Mercure et al., 2016a). People and firms in these models are represented by a representative agent with rational

expectations. There are various inherent limits for informing policy making if the model does not take into account agent heterogeneity. In the equilibrium model, agents all respond in the same way to changes in government policy. Without social influence, the market shares for the technology will change only if the incentives change (Mercure, 2016a).

Existing studies have shown the important influence of behavioural assumptions on policy-relevant outcomes in the diffusion of low emissions vehicles (Li, 2017, Mau et al., 2008, Pettifor et al., 2017a,b). This is because individual decisions are strongly affected by social norms and customs when choosing a car (McShane et al., 2012). Instead of instantaneous change in shares when an incentive is imposed, the technology diffusion will be shaped by a new diffusion trajectory. However, in the optimization model, as long as solutions remain with the multidimensional box of constraints, without considering any self-reinforcing effects, the shares for technologies rise instantaneously when an incentive is imposed and this is not consistent with the diffusion trajectory of technology (i.e. market shares only change when incentives change for the optimisation model, while in a diffusion process, the market shares change without the need for a change in incentives).

Capturing behavioural realism in consumer preferences of passenger cars in global IAM increases their usefulness to policy makers. Modellers have attempted to incorporate some behavioural realism in existing global IAMs. For instance, Pettifor et al. (2017b), Wilson et al. (2015) represents heterogeneous consumer groups for vehicle choices with varying preferences for vehicle range and variety in the MESSAGE model. While this approach considers consumer heterogeneity by segmentation, the MESSAGE model remains an optimisation model without self-reinforcing effects present in the diffusion of technologies (McCollum et al., 2016a).

The socio-MARKAL model integrates technological, economic and behavioural contributions to the environment in a few cities (e.g Nyon) (Nguene et al., 2011a). Similarly, Daly et al. (2014) incorporates travel behaviour into the TIMES model by accounting for individual travel budget constraint for Canada and Ireland. Bunch et al. (2015) incorporates behavioural content from MA3T (Market Acceptance of Advanced Automotive Technologies) into the TIMES model. The MA3T simulates vehicle market behaviour over time, where the core behavioural model is a nested multinomial logit discrete choice model that yields market shares of competing technologies for a large number of consumer segments. However, the MA3T is limited to projecting the behaviour of the vehicle market in the US under alternative policy scenarios.

While the above research does improve the behavioural realism of the global IAMs, it is possible to improve further the representation of consumer behaviour with the FTT-Transport model. Firstly, The E3ME-FTT-Transport model is an attempt to model consumer preferences change over time as a result of changes in trends, fashion and income. Secondly, in the E3ME-FTT-Transport model, consumer behaviour is integrated into the IAM in a global scale. This is different to the existing equilibrium models where the research mostly focus on a few countries and regions. Thirdly, with a improved representation of consumer behaviour, the model allows a higher numbers of policy levers in the model for the analysis of detailed policy incentives.

2.5.1. Modelling choice with intangibles

People's purchasing behaviour is not only affected by financial costs, but also by comfort, luxury, practicality and aesthetics (Axsen and Kurani, 2012, McShane et al., 2012). However, since these behavioural parameters are difficult to quantify, few attempts have been made to include intangible costs in technology models. With few exceptions, SOCIO-MARKAL modelled behaviour through sociological surveys in order to capture the perception of the population regarding energy consumption (Nguene et al., 2011b). Similarly, the CIMS user can specify an intangible cost factor to characterise estimated real-world consumer preferences (Jaccard et al., 2003). Focusing on residential energy projection, both the MESSAGE-Access model and the IMAGE-REMG model introduce 'inconvenient costs' that capture some of the non-monetary aspects of households preferences (Farsi et al., 2007, Maconachie et al., 2009, Pachauri et al., 2013).

Similarly, in FTT: Transport, intangible costs are included in the consumer decision function to represent all the perceived costs (or benefits) of a technology that are not derived from its financial attributes. Since survey data is not available on a global scale, the intangible costs are empirically determined from historical data for each technology.

2.6. Policy assessment for emissions from the transport sector

Energy models are useful for policy makers to assess the impact of policy incentives on the emissions from the transport sector in the long term. At a national level, a number of models have been applied to analyse the effectiveness

of policy incentives on emissions reduction. For instance, Kloess and Müller (2011) investigates the effect of various tax incentives and technological progress on the Austrian passenger car fleet. Using the UK Transport model, Brand et al. (2013) assesses the long term scenario of low carbon fiscal policies and their effects on transport demand. McCollum and Yang (2009) examines the potential of deep cuts in US transportation using Long-term Evaluation of Vehicle Emission Reduction Strategies (LEVERS) model. Alam et al. (2017) modelled passenger car fleets in Ireland from 2015 to 2050 to assess the impact of current and potential greenhouse gas mitigation policies using the integration of the COPERT model and the W2W model to assess the impact of current and potential greenhouse gas mitigation policies.

Most models for detailed policy analysis have been focused on a particular region or country. The shortcoming of studying one country is that such models have limited use in terms of the transport policy incentives in the global climate change context. Global IAMs are coupled with land use, agricultural and climate change models, and have been used to assess the medium to long term impact of transport policy on global climate change. With the policy analysis tool linked to a global IAM, it is possible to derive insights on the systematic consequence of technology policy options in the transport sector.

In order for global IAMs to be useful for policy assessment, it is crucial to feature at least a few policy instruments that realistically represent real world climate policy. While in reality, in the transport sector, climate policies feature a wide range of different types of incentives, most IAMs feature a few, sometimes only a single, policy lever for decarbonisation, a carbon price that is applied to all sectors targeted by the climate policy. As argued by Grubb (2014), it is likely that a carbon price alone will not be sufficient to achieve the climate target. Moreover, all Emissions Trading Schemes to date exclude road transport, so there is little reason to study the impact of a carbon price on road transport. To be realistic, models needs to study the impact of at least vehicle taxes, road taxes, fuel taxes, regulations, standards and biofuel mandates, since these are very common across the world, often all used simultaneously. Some recent studies recognise this limitation of current IAMs and integrate additional policies, including fuel taxes, registration or road use (Deetman et al., 2013, Yin et al., 2015, Zachariadis, 2005); Technology subsidies and mandates (Bertram et al., 2015, Deetman et al., 2013, Yin et al., 2015, Zachariadis, 2005); efficiency policies (Deetman et al., 2015, Siskos et al., 2015, Yin et al., 2015, Zachariadis, 2005), biofuel blends and mandates (Calvin et al., 2014). The FTT model includes eight policy instruments (vehicle taxes, registration taxes, fuel taxes, vehicle subsidies, regulations, fuel economy standards, biofuel mandates and possible kick-start programs) and can be used to model policies for each individual countries.

Policy assessment in the transport sector requires coping with a large number of decision makers, involved complex interaction between consumers and vehicle markets. However, the existing global IAMs are predominantly optimization models, assume a social planner and no interactions between consumers. In some interpretations, this implies that agents respond to policy incentives in a collective manner based on system-wide cost-minimisation or utility-maximisation criteria. However, in reality, no-one in society faces the system cost, each individual faces his/her own costs and benefits in his/her own social context. Therefore, while a social planner perceives tradeoffs between, for example, vehicle fleet carbon costs and power generation carbon costs, and can trade them for one another under the prevailing carbon price, in reality investment happens independently by different people in different sectors, and different sectors use different ranges and types of policies.

3. Detailed theoretical model description for FTT:Transport

The theory is presented here. A list of variable definitions is given in appendix B.

3.1. Basic equations

3.1.1. Transport demand

The model starting point is with an exogenous total transport service demand $D(t)$, in million vehicle-kilometres per year (denoted Mvkm/y), and a fleet size $N(t)$, in thousands of vehicles (denoted kv), derived from vehicle sales, $\xi(t)$. In FTT:Transport, we consider the choice of purchasing a vehicle, which happens every few years, quite independent from the decision to use a vehicle, which is a daily decision. Thus, depending on conditions, people may purchase vehicles that are later not used as much as expected (e.g. say due to higher than expected fuel prices), or more than expected. Transport services are also generated for people who do not own vehicles. Hence we assume that the vehicle owner and the vehicle user are different agents, where, to keep the model concept and equations clear, the vehicle owner sells a transport service to the vehicle user, even when both are the same person.

For that reason, the model features two independent econometric specifications as part of the macroeconometric model E3ME, one for the demand for transport $D(t)$ and one for the demand for new vehicles (i.e. sales) $\xi_{tot}(t)$ (in kv/y), extrapolated from historical data using a number of socio-economic parameters (we come back to this in section 5.4). D is obtained using chosen econometric relationships, function of macroeconomic variables: economic growth, employment, fuel prices, etc, while sales $\xi(t)$ are determined from disposable income and vehicle prices and operation costs. The role of FTT:Power is primarily to determine which transport technologies supply these demands, populating vehicle fleets, and which type and quantity of energy the fleets use.

Vehicles survive for a number of years statistically, something that we discuss in more detail in section 3.3.1, giving a total number of vehicles $N(t)$ that is endogenous and dependent on the sales $\xi(t)$ (we assume the survival rate constant):

$$N(t) = \int_0^\infty \xi(t-a)\ell(a)da, \tag{1}$$

where $\ell(a)$ is the survival function, the probability of a vehicle to still be operational on the road at age a, i.e. a years after purchase (see sections 3.3.1 and 4.3.2 for information on the survival function). Thus the fleet is composed of vehicles with a mixture of ages that depends on how many vehicles were purchased in each past year and their probability of having survived to the present (see Mercure, 2015, for an exposition on technology survival).

We assume here that sales and the demand for transport services is independent of the type of engine that vehicles have. This may not always be true, if for example, infrastructure for electric vehicles is not as extensive as that for liquid fuel Internal Combustion Engine (ICE) vehicles, or motorcycles which not everyone may like. Nevertheless, this makes our model simple, tractable and useable at a global scale with the data that we have, and we consider it good enough for the purpose at hand.

The relationship between the two independent variables $D(t)$ and $N(t)$ generates an endogenous average capacity factor $\overline{CF}(t)$, which expresses to which degree, or intensity, vehicles are used (in Mvkm/y/kv = k-km/y),

$$D(t) = \overline{CF}(t)N(t). \tag{2}$$

We have designed FTT:Transport to calculate transport generation in person kilometres rather than vehicle kilometres, enabling to include vehicles with different numbers of seats, notably motorcycles. Road transport technologies (e.g. petrol, electric, motorcycles) by class (economic, mid-size, luxury), indicated with a subscript i, generate each a transport service component G_i (in million person-kilometres per year, Mpkm/y), using a total seating capacity $U_i(t)$ (in thousands of seats, ks),

$$G_i(t) = CF_i(t)U_i(t) = FF_i N_i d_i(t)U_i(t), \tag{3}$$

where $CF_i(t)$ is now technology dependent, and further subdivided into an average filling factor (occupancy rate) FF_i (number of passengers per seat, p/s), determining how full vehicles are on average when they travel, which we consider constant over time, as well as an average distance travelled per year $d_i(t)$ (km/y), which is itself function of the fraction of time vehicles are used and at what average speed. $CF_i(t)$ varies mainly between vehicles and motorcycles, which on average travel shorter distances per year.

In the order of the calculation, we first determine $U_i(t)$ in order to obtain $G_i(t)$. For this, we define the transport technology market share:

$$S_i(t) = \frac{U_i(t)}{N(t)}, \quad N = \sum_i U_i. \tag{4}$$

We follow the lines of the main paper and of section 3.3.3, referring the reader to earlier work for a highly detailed treatment (Mercure, 2015). Shares, first determined from historical data, evolve over time following replicator dynamics equation, which is explained in section 3.3.3. The replicator dynamics is the source of dynamical behaviour of FTT models, and as we see further down, represents technology diffusion following S-shaped curves.

$$\Delta S_i = \sum_j S_i S_j \left(\frac{F_{ij}}{\tau_i} - \frac{F_{ji}}{\tau_j} \right) \Delta t. \tag{5}$$

The average capacity factor $\overline{CF}$ is obtained from shares,

$$D(t) = N(t)\overline{CF}(t), \quad \overline{CF}(t) = \sum_i S_i(t) FF_i N_i d_i(t). \tag{6}$$

This enables to write the capacity in terms of the demand and shares quite conveniently:

$$U_i(t) = \frac{S_i(t)}{\overline{CF}(t)} D(t). \tag{7}$$

In this equation, the number of vehicles in a category can change for three independent reasons: (1) the total demand can change, (2) the technology composition can change and (3) the efficiency at which vehicles are used can change. This is summarised by the following differential form:

$$\Delta U_i = \frac{S_i}{\overline{CF}} \Delta D + \frac{D}{\overline{CF}} \Delta S_i - \frac{S_i D}{\overline{CF}^2} \Delta \overline{CF}. \tag{8}$$

These are three chosen independent variables that influence the evolution of the numbers of vehicles by category; all other variables are by construction functions of these three.

3.1.2. Fuel use and emissions

One of the main outputs of this model are the emissions of greenhouse gases and other pollutants of type j (in t/y, e.g. tCO_2/y):

$$E_i^j = J_i \alpha_i^j, \tag{9}$$

where J_i is the amount of fuel used by vehicles of type i, while α_i^k is the emissions factor for pollutant of type k emitted by technology of type i, a property specific to the fuel used by that technology. In the case of petrol and carbon dioxide, α_i^k has units of tCO_2/GJ. However in the case of electric vehicles, no emissions of pollutants occur at the vehicle level.

Fuel use is function of vehicle use, and in most cases does not vary by large amounts whether the vehicle is used at capacity or not. It does not scale proportionally to the filling factor, but is a rather complicated function of that variable, depending the speed profiles, purpose of driving and driving habits, where more acceleration means higher fuel use for larger filling factors. Such information is not readily available, and therefore, as an approximation, fuel use is assumed independent of the filling factor of vehicles. Vehicle of type i uses fuel of type k (in PJ/y),

$$J_i^k(t) = \frac{G_i(t)}{FF_i} \beta_i^k, \tag{10}$$

where β_i^k is the fuel consumption per kilometre driven (in MJ/vkm) for technology i, a property of the technology.

3.1.3. Investment, learning and cost reductions

One of the reasons for calculating the capacity U_i is that costs evolve with the cumulative production of vehicles due to learning by doing. Each year a certain amount ξ_i of transport capacity of type i comes out of factories and is registered, sold at cost IC_i, generating investment $I_i = IC_i\xi_i$. Registrations correspond to positive increases in numbers of vehicles plus the replacement of scrapped vehicles, while negative changes correspond to destructions that are not replaced.

$$\xi_i(t) = \begin{cases} \frac{dU_i}{dt} + \frac{U_i}{\tau_i}, & \frac{dU_i}{dt} > 0 \\ \frac{U_i}{\tau_i} & \frac{dU_i}{dt} \leq 0 \end{cases},$$

where the term $U_i/\overline{\tau}$ refers to vehicle replacements, with $\overline{\tau}$ a technology life expectancy (Mercure, 2015).

Learning curves are expressed as cost reductions that occur with the cumulative production of units of technology, or in our case, cumulative sales of vehicles. The usual form in which learning curves are expressed is

$$IC_i(t) = IC_{0,i}\left(\frac{W_i(t)}{W_{0,i}}\right)^{-b_i}, \tag{11}$$

where W_i is the cumulative investment and the pair $IC_{0,i}$, $W_{0,i}$ are corresponding costs and cumulative sales at a particular point in time, taken here as the start of the simulation and b_i is the learning exponent, related to the learning rate.[3] Learning however happens on a component level rather than at the technology level (e.g. engines, batteries, materials), which may be used in more than one type of technology, and therefore sales in one technology category may induce learning in other categories. A spillover matrix B_{ij} is thus defined, mixing the learning:

$$W_i(t) = \sum_j B_{ij}\int_0^t \xi_j(t')dt', \quad W_{0,i}(t) = \sum_j B_{ij}\int_0^{t_0} \xi_j(t')dt'. \tag{12}$$

3.2. The decision-making model core

3.2.1. Perceived costs and decision-making

We detail further here our model of decision-making in the context of diverse agents. For a model of technology diffusion, we require an aggregate representation of decision-making when agents are diverse, and costs have variations. This core model component is evaluated at every time step, and the decision-making determines the composition of new technology units purchased, which in time gradually changes the overall transport fleet.

Diversity stems from different perceptions from agents when they take a decision, which may originate from a large set of particular preferences and constraints that is impossible to enumerate in a model. We summarise this by distributions. We assume that choice is made on the basis of a single quantity, a generalised cost, evaluated by agents for each option they see as available to them, and this value features a quantification of all possible aspects that weigh in the decision-making balance.

We postulate here that *distributions of perceived costs correspond to distributions of observed costs*, with a possible constant offset between them. People, we assume, when considering purchasing a vehicle, most likely choose something they have seen being purchased, perhaps by someone they know such that they were able to gather information (i.e. they most likely do not choose something they know nothing of, and they gather reliable information predominantly through observations of their peers). Their observations of the fleet is a subset of what is on roads, and every agent observes something slightly different from every other. This may be due to their belonging to a particular social group and social class, and they are most likely to choose amongst what their peers have previously chosen, which itself is a subset of what the whole market has to offer (e.g. poor rural households perhaps purchase different types of vehicles to rich suburban families, which itself is different than single middle-class persons, i.e. their peers are a subset of the population and their observations are a subset of all observations). Thus we assume *restricted technology/information access*, in other words, agents do not choose what they do not know, and they do not know all of the market (or even perhaps do not care for all of the market). *Importantly, this means that FTT does not have a*

[3] The learning exponent is $b_i = \ln(1 - LR_i)/\ln(2)$, where LR_i is the learning rate.

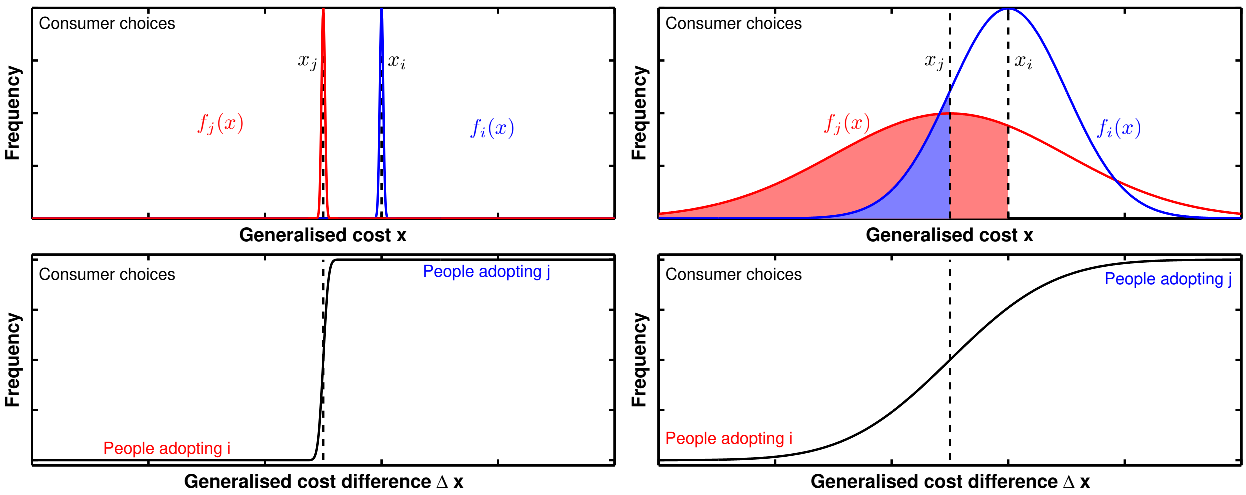

Figure 1: Illustration of the process of decision-making under diversity of agents between two technologies. The blue curve represents the distribution of perceived generalised costs for one technology, and the red curve that of the other. In the left panel, if diversity is very low, choices can flip very abruptly as average costs cross. This corresponds to the representative agent case. In the right panel, introducing significant diversity makes choices distributed and choices change very gradually as costs cross.

representative agent. Choices of particular social groups endure through peer observation and visual influence, which has been demonstrated is the case for vehicle purchases in McShane et al. (2012).

The frequency of observations of a particular model (by consumers shopping), sample of an ensemble of such events, corresponds to the frequency of recent sales of that model (purchases by their peers). We then postulate that the probability of choosing a particular model is proportional to this frequency of observation, and thus these preference distributions, associated to circumstances and constraints of consumers difficult to enumerate and unknown to the modeller, are relatively stable. These combined frequencies form a generalised cost distributions of sales (see for example the sales distribution in Figure 2). In this perspective, *the generalised cost distribution of recent sales is a representation of the diversity (hegerogeneity) of choices*. We go further and say that we can use the measured heterogeneity of sales and interpret it in terms of the heterogeneity of agents. Furthermore, in such a perspective, vehicle sales by vehicle model reinforce the sales of those very models, consistent with sociological evidence (see e.g.McShane et al. (2012)).

3.2.2. Diversity is crucial

The importance of diversity must be emphasised. Its is often taught in basic diffusion theory that the various parts of the diffusion curve belongs to different types of consumers: early adopters, middle adopters, followers, laggards, etc. This crude picture is useful here, as it connects the notion of diversity to a rate of technology adoption. We show below that it is a very important assessment of the problem, as follows.

Vehicle purchases are distributed in time, by consumers that take different decisions at different points in time for different reasons. If we were to imagine, temporarily, that agents had identical preferences and constraints, were there to be only two or more options for vehicles in the market with non-identical properties, they would nevertheless always all choose the same technology. We could furthermore temporarily imagine that agents know all of the market perfectly, and thus know about all these vehicle models that no one buys, and finally consider (by construction) that the vehicle model that everyone keeps buying is the less expensive in terms of generalised cost. Then, if for one reason or another, the generalised cost difference between of one of the unused technologies and the common one was to cross zero, then from then onwards all agents would simultaneously change their preference and the adoption of the new vehicle type would be instantaneous were it not for probable industrial supply problems. If we depict this situation using cost distributions for technologies and a cumulative probability distribution for choice in terms of a cost difference, we obtain the representation shown on the left panel of fig. 1. We know that this is an unrealistic representation. When we make abstraction of industrial growth dynamics, the diffusion of technology is a process that results from consumer heterogeneity.

We take a *diverse* group of technologies purchased by a *diverse* group of consumers. Comparing these technologies based on consumer choices (i.e. on the basis of their generalised cost) leads to a comparison of frequency distributions, shown in the right panel of fig. 1. That these distributions have unequal means signifies that one is on average less expensive than the other. However this does not mean that this is the case in all individual situations where a consumer makes a choice, in his own perspective, but it should be the case a major fraction of decisions. Thus if the generalised cost difference, say the difference between the means, gradually decreases to zero, at each value of this difference a larger fraction, but not all, of consumers will choose the technology which is on average the less expensive, until when means are equal exactly half of consumers choose each. As the cost difference crosses zero, this fraction decreases gradually below 50%. The resulting profile of adoption is then a very gradual one, the steepness of which depends on the widths of the distributions (the degree of heterogeneity), as we show next, and this results from all consumers having slightly different perspectives.

3.2.3. Pairwise choices comparisons of distributed choices

FTT:Transport operates by using chains of binary logits, which is made clear in section 3.3.3. We describe here the binary logit itself, i.e. preferences in a group of heterogenous agents for every possible pair of options. Combining them, with the unequal frequencies at which they take place, will later yield the replicator equation.

We assume that we have two cost distributions for two vehicle types for what we assume are the relative numbers of situations where agents, stating their individual preference between technologies i and j, face different situations and state different choices. By counting how many agents prefer which technology in each pair, one can state what the probabilities of preferences between these two technologies are for future situations where choices are to be made (e.g. 70% of agents choose i and 30% j).

We denote these generalised cost distributions $f(C, C_i, \sigma_j)dC = f_i(C - C_i)dC$ and $f(C, C_j, \sigma_j)dC = f_j(C - C_j)dC$, where C_i, C_j are the mean generalised costs and σ_i, σ_j are their standard deviations, for technologies i and j. These distributions can be of any kind, but they require to have a single well defined maximum and variance (e.g. they cannot have two maxima[4]). For FTT:Transport, we found that these are lognormal, and therefore we make cost comparisons in log space.

We evaluate the probability of choosing i over j using the following. First, we calculate the probability of choosing i in all cases where j has an arbitrary cost C. Our central assumption is that the fraction of agents for whom the generalised cost of j is C and for whom the cost for i is lower than C will choose technology i over j if given a choice, and this fraction is equal to the cumulative probability distribution $F_i(C - C_i)$. But this situation occurs a fraction $f_j(C - C_j)$ of the time, giving a total probability

$$P(C_i < C | C_j = C) = F_i(C - C_i) f_j(C - C_j) dC, \tag{13}$$

while the converse is

$$P(C_j < C | C_i = C) = F_j(C - C_j) f_i(C - C_i) dC. \tag{14}$$

In order to know how often the cost of technology i is lower than that of technology j, and the converse, we must sum over all possible values of C. For simplicity, we use as variables $C' = C - C_j$ and $C'' = C - C_i$, with the mean cost difference $\Delta C = C_i - C_j$:

$$F_{ij}(\Delta C) = P(C_i < C_j) = \int_{-\infty}^{+\infty} F_i(C' - \Delta C) f_j(C') dC',$$

$$F_{ji}(\Delta C) = 1 - F_{ij} = P(C_j < C_i) = \int_{-\infty}^{+\infty} F_j(C'' + \Delta C) f_i(C'') dC''. \tag{15}$$

This appears difficult without further knowledge of the distribution type, however we can use a simple reasonning: we take a derivative with respect to ΔC, which makes the integral a convolution of the two distributions

$$\frac{dF_{ij}}{d\Delta C} = -\int_{-\infty}^{+\infty} f_i(C' - \Delta C) f_j(C') dC' = -f_{ij}(\Delta C) = \int_{\infty}^{-\infty} f_i(C'') f_j(C'' + \Delta C) dC'' = -f_{ji}(-\Delta C) = \frac{dF_{ji}}{d\Delta C}. \tag{16}$$

[4] In which case we would need to subdivide such a technology category into two.

As derived in appendix A, this convolution yields a new distribution $f_{ij}(\Delta C)d\Delta C$ of which the standard deviation is $\sigma_{ij} = \sqrt{\sigma_i^2 + \sigma_j^2}$. This is the probability distribution of technology switching in terms of ΔC. The difficult integral having been computed, this distribution can be integrated again as a function of ΔC to yield a cumulative probability distribution that technology i is less expensive than j (and conversely):

$$F_{ij}(\Delta C) = \int_{-\infty}^{+\infty} f_{ij}(\Delta C)d\Delta C = 1 - \int_{-\infty}^{+\infty} f_{ji}(\Delta C)d\Delta C = 1 - F_{ji}(\Delta C). \tag{17}$$

Thus given a choice between technologies i and j, the fraction F_{ij} of agents tends to choose technology i and the fraction F_{ji} chooses j, these fractions being functions of the generalised cost difference, and this function has a standard deviation that follows the sum of the squares $\sigma_{ij} = \sqrt{\sigma_i^2 + \sigma_j^2}$. Note that this calculation is independent of probability distribution type; however $F_{ij}(\Delta C)$ should have roughly the shape of a 'smooth' step function, its 'smoothness' determined roughly by the combined root mean square widths of *both* cost distributions.

Note that if there is a transformation under which $f_i(C - C_i)dC$ is a normal distribution, then $f_{ij}(\Delta C)d\Delta C$ is also normally distributed (the convolution of normal distributions together yields normal distributions, see appendix A). In the case of FTT:Transport, costs are lognormally distributed, and therefore so is $f_{ij}(\Delta C)d\Delta C$. However the logistic function is a relatively close approximation of the normal distribution, but much faster to calculate than the error function, and thus in FTT:Transport, we compare logistic distributions of the log of the generalised cost.

3.2.4. *Relationship to discrete choice modelling and the binary logit model*

This model is equivalent in many respects to a binary logit model, as initially derived by McFadden (see Ben-Akiva and Lerman, 1985b, Domencich and McFadden, 1975). In the binary logit model, one assumes that heterogenous consumers maximise their individual utility (or minimise their cost), which is defined in terms of several factors including prices but also other non-monetary factors. The cost associated with choices i and j for a population is written in terms of an average value and a distributed value ϵ_i of mean zero and standard deviation σ), which represents consumer diversity (i.e. differences of perception, situations and constraints, which are unknown to the modeller):

$$C_i = \overline{C}_i + \epsilon_i \quad C_j = \overline{C}_j + \epsilon_j. \tag{18}$$

The difference in cost $C_i - C_j$ is thus also distributed. The assumption is taken that ϵ varies following a Gumbel distribution located at the mode value C_i

$$f_i(C) = \frac{1}{\sigma} \exp\left(-e^{\frac{C-C_i}{\sigma}}\right) e^{\frac{C-C_i}{\sigma}} \quad F_i(C) = \exp\left(-e^{\frac{C-C_i}{\sigma}}\right). \tag{19}$$

This distribution is common whenever a distribution is made of the maxima of underlying distributions, as in extreme value theory. Among others, the Gumbel distribution has the property that a distribution of the extreme values of Gumbel distributions follows a Gumbel distribution. It also has the property that the sum of two Gumbel distributed values follows a logistic distribution, an aspect used here. Thus the probability of consumers with distributed generalised costs (or utilities) choosing technology i over technology j is

$$P_i(\overline{C}_i + \epsilon_i > \overline{C}_j + \epsilon_j) = P_i(\epsilon_i - \epsilon_j > \overline{C}_j - \overline{C}_i), \tag{20}$$

which involves calculating the difference between both distributions $\epsilon_i - \epsilon_j$. This corresponds to a convolution of $f_i(C)$ with $f_j(C)$ (as done above), equal to a logistic distribution:

$$f_{ij}(\Delta C_{ij}) = \int_{-\infty}^{\infty} f_i(C' - \Delta C_{ij}) f_j(C') dC' = \frac{\exp\left(\frac{\Delta C_{ij}}{\sigma}\right)}{\left(1 + \exp\left(\frac{\Delta C_{ij}}{\sigma}\right)\right)^2}, \tag{21}$$

with a cumulative distribution which is a logistic curve:

$$F_{ij}(\Delta C_{ij}) = \frac{1}{1 + \exp\left(\frac{\Delta C_{ij}}{\sigma}\right)} = \frac{\exp(C_i/\sigma)}{\exp(C_i/\sigma) + \exp(C_j/\sigma)}. \tag{22}$$

This result is nearly identical to the one above, with the difference that the result above is more general with technology specific diversity parameters which follow standard error propagation, $\sigma_{ij} = \sqrt{\sigma_i^2 + \sigma_j^2}$, and arbitrary distribution forms, while in the logit model it is explicitly assumed that the widths and shapes of the distributions are identical and that the distributions are Gumbel, leading to a logistic choice function of the difference in generalised cost (or utility).

3.2.5. Correspondence between consumer diversity and elasticities of substitution

The probability of change of choice given a change in context, for instance prices, is generally expressed with elasticities and cross elasticities of substitution, defined in this context as

$$\frac{\frac{\partial F_{ij}}{F_{ij}}}{\frac{\partial C_i}{C_i}} = \frac{\partial \log F_{ij}}{\partial \log C_i} = \frac{\partial F_{ij}}{\partial C_i}\frac{C_i}{F_{ij}} \quad \text{and} \quad \frac{\partial F_{ij}}{\partial C_j}\frac{C_j}{F_{ij}} \tag{23}$$

This can be computed using eq. 22:

$$\lambda_{ii} = \frac{\partial F_{ij}}{\partial C_i}\frac{C_i}{F_{ij}} = -\frac{C_i}{F_{ij}}\frac{1}{\sigma}\frac{\exp\left(\frac{\Delta C_{ij}}{\sigma}\right)}{\left(1+\exp\left(\frac{\Delta C_{ij}}{\sigma}\right)\right)^2} = -\frac{C_i}{\sigma}F_{ji}, \tag{24}$$

$$\lambda_{ij} = \frac{\partial F_{ij}}{\partial C_j}\frac{C_j}{F_{ij}} = \frac{C_j}{F_{ij}}\frac{1}{\sigma}\frac{\exp\left(\frac{\Delta C_{ij}}{\sigma}\right)}{\left(1+\exp\left(\frac{\Delta C_{ij}}{\sigma}\right)\right)^2} = \frac{C_j}{\sigma}F_{ji}. \tag{25}$$

These elasticities can also be calculated using time series data, if available, of F_{ij} against parameters that include C_i and C_j:

$$\Delta \log F_{ij} = \lambda_{ii}\Delta \log C_i + \lambda_{ij}\Delta \log C_j. \tag{26}$$

This generates a connection between elasticities of substitution, as calculated from measured time series, and the diversity of consumer behaviour, as measured using price distributions. If we define a diversity parameter as the standard deviation of consumer revealed preferences for technology i, measured using sales data as done in this work, normalised by the average cost of that technology, $2\sigma/C_i$, then when costs are similar, the logistic form of F_{ji} in eqns. 24-25 can be linearised

$$\lambda_{ii} \simeq -\frac{C_i}{\sigma}\left(\frac{1}{2} + \frac{\Delta C_{ij}}{4\sigma}\right) \simeq \frac{C_i}{2\sigma}, \quad \lambda_{ij} \simeq \frac{C_j}{2\sigma}. \tag{27}$$

We thus find that *the elasticity is inversely proportional to consumer diversity*. This can be understood as follows: when diversity is low, consumers tend to all act similarly simultaneously, and this results in price changes having an impact on the whole population, leading to important changes of preference. Meanwhile, when the diversity is high, price changes may have an impact only on a subset of the population, leading to small changes of preference.

3.2.6. The levelised cost of transportation

For the decision-making component of this model, we separate the *investor* in transport technology from the *consumer* of transport services. We think of them as separate entities for clarity, even though in some cases they might happen to be the same person. Whether the roles are fulfilled by the same actors or not, they are quite distinct, where the *investor* purchases a vehicle to sell a transport service to the *consumer*. This is done in order to clarify the distinction between technology investment and associated market competition, and the consumption of the service technologies produce. It also allows for that even when a person purchases a car, he/she can still travel by train/plane (i.e. not use the car he purchased): the mode choice is distinct from the technology choice, even when performed by the same person.

The cost of the vehicle, as perceived by the investor purchasing a vehicle or unit of transport technology, must be taken to include all components relevant to the decision making. Many of the components are easy to quantify from available data. Others are not straightforward, and we show here how this is done. When a vehicle is purchased, an initial investment is made, or a loan is obtained, for the capital cost, and henceforth fuel and maintenance costs are

incurred for the lifetime of the technology. In addition to this taxes may be added either as a fixed initial cost or as a yearly fee, or both.

Following the main text of the paper, we define, as a component of the decision-making process, the Levelised Cost of Transport services (LCOT), before policies are applied (in section 3.5 we add policies):

$$LCOT_i = \sum_t \frac{\frac{I_i}{CF_i(t)} + \frac{FU_i(t)}{\beta_i} + \frac{MR_i}{FF_i}}{(1+r)^t} \Big/ \sum_t 1/(1+r)^t, \tag{28}$$

where each term is identified in the main text of the paper (see the list of variables in appendix B).

Several terms in eq. 28 are distributed, while others are single valued. Investment cost distributions can be assigned to a distribution of preferences, but variations can also arise in all other parameters. Since vehicles considered in each category are highly distributed in every one of their characteristics (emissions, price, engine size), most of these parameters are distributed, for example energy use. The discount rate could also be distributed, but we have not included this at this stage. It is to be kept in mind however that in a root mean square calculation, any dominating parameter rapidly makes smaller contributions negligible. Here, the vehicle price distribution dominates (it has the largest standard deviation), but we nevertheless keep energy use and maintenance parameters distributed.

As we show in appendix A, the distribution of the sum of two distributions corresponds to their mutual convolution, and therefore the sum of several distributions corresponds to a chain of convolutions of all of these. As a result, means are added while the standard deviations are combined using the root of the sum of the squares of the individual standard deviations, as follows (using eq. A.7), leading to

$$\Delta LCOT_i = \frac{\sum_t \frac{\sqrt{\frac{\Delta I_i^2}{CF_i^2} + \frac{I_i^2}{CF_i^4}\Delta CF_i^2 + \frac{\Delta F_i^2}{\beta_i^2} + \frac{F_i^2}{\beta_i^4}\Delta\beta_i^2 + \frac{\Delta MR_i^2}{FF_i^2} + \frac{MR_i^2}{FF_i^4}\Delta FF_i^2}}{(1+r)^t}}{\sum_t \frac{1}{(1+r)^t}}. \tag{29}$$

This standard deviation of the generalised cost, $\Delta LCOT_i$, is our model representation of agent heterogeneity, parameterised by data. Policies are assumed not distributed; their possible distributional impacts will stem from other distributed parameters already specified here.

3.2.7. Using log-normal distributions

Costs, as we show in the data section, are generally distributed asymmetrically and are almost always well described by log-normal distributions, as are many economic processes. As we have just shown, the calculation of preferences F_{ij} does not depend on a particular form of distribution. However, given this property of the data, the generalised cost comparison is better performed in logarithmic space than in real space. For this, one only needs to convert the means and the variances of the distributions measured in real (dollar) space into values measured in logarithmic space, with the following transformations:[5]

$$\mu = \ln\left(\frac{m^2}{\sqrt{v+m^2}}\right), \tag{30}$$

$$\sigma = \sqrt{\ln\left(1+\frac{v}{m^2}\right)}, \tag{31}$$

where μ and σ are the mean and standard deviation in logarithmic space, and m and v are the mean and standard deviation in normal dollar space. Thus a simple conversion can be made.

In FTT:Transport, the cost comparison is made in logarithmic space, in other words C_i above is the log of a cost in \$/pkm. Thus the LCOT and its standard deviation are calculated using eq. 28 and 29, and subsequently converted using these transformations before carrying out comparisons. This is consistent with the fact that the income distribution is usually lognormally distributed as well.

[5]See the wikipedia page on lognormal distributions.

3.2.8. *The generalised cost as a comparison measure*

As it is inferred from price distributions of sales, transport cost considerations are not the only elements of consumer decisions when purchasing a vehicle. Many additional aspects are valued by the consumer, of which we have little information beyond the price distribution of what they purchase. However, we do have existing trends of diffusion from historical data (section 5.2). We keep in mind that technologies have highly different pecuniary costs, particularly across engine size classes; and despite this, higher costs appear compensated by higher benefits, such that higher cost luxury vehicles maintain market shares.

Were we to simulate technology diffusion based on bare LCOT distribution comparisons, the lowest LCOT technologies would diffuse more successfully, which, as it turns out, is not consistent with our historical data. Clearly, components would be missing in the LCOT, for instance comfort, acceleration, style, that we may call the 'intangibles'. We define these 'intangibles' for this model as the difference between the generalised cost, which leads to observed diffusion, and the LCOT as calculated from pecuniary vehicle properties for which we have data. The value of the intangibles, denoted γ_i, is an empirical parameter that we obtain from making the FTT diffusion trajectory match the trajectory observed in our historical data, at the year of the start of the simulation. The theory goes as follows, while the practical method is described in section 5.5.

The diffusion of technologies in a set takes place at the expense of one another in market share space. According to sections 3.2.3 the choice of investors is made based on pairwise comparisons of generalised cost distributions. Based on the transformations above, we use the following pair:

$$C_i = \ln\left(\frac{LCOT_i^2}{\sqrt{LCOT_i^2 + \Delta LCOT_i^2}}\right) + \gamma_i, \tag{32}$$

$$\Delta C_i = \sqrt{\ln\left(1 + \frac{\Delta LCOT_i^2}{LCOT_i^2}\right)}. \tag{33}$$

When $\gamma_i = 0$, we obtain a rate of diffusion that does not normally match historical diffusion. One, and only one, set of γ_i leads to the diffusion of technology in the simulation to have the same rate as the historical rate at the starting point of the simulation. We describe this in section 5.5. The interpretation of the γ_i parameters is that they ensure that FTT projects in the future a diffusion trajectory (the rate of change of shares) that is the same as what is observed in historical data, and represents all costs not explicitly specified as perceived by agents. The γ_i are not distributed, and thus, are not to be associated with our representation of agent heterogeneity.

3.3. *Population changes as a result of decision-making*

3.3.1. *Survival rates and technology vintages*

The connection between sales and actual vehicle numbers in a fleet depends on the length of time that vehicles survive for. This is described by standard survival (or reliability) analysis (e.g. Liu, 2012). Vehicles come to the end of their useful life through various events or processes: accidents, failures or scrapping decisions. The rate of changes in the system depends on this length of time, which determines the size of the markets for second-hand and new vehicles. As we show in section 4.2.3, observations of the UK fleet are available, making possible the parameterisation of vehicle survival rates. This requires a bit of theory on vehicle survival, as follows. This analysis enables to parameterise the rates of fleet turnover, a component of the rates of diffusion. It also determines at which rate a fleet *can* physically be transformed. A detailed theoretical analysis is given in Mercure (2015).

Vehicles remain on roads for a length of time until they are scrapped, for one of the three reasons given above. For the vehicle fleet and its size, the nature of ownership, and the number of different owners of a vehicle along its life is not important in the perspective of this model. When vehicles are purchased, they remain in the system until they are scrapped, irrespective of their number of owners. What is important is the statistics of its survival: what its probability of making it to a certain age, which is in general very well defined. This is commonly termed the survival function.

The fleet in year t has an age distribution, which we denote $n_i(a, t')$, where i denotes the technology type, a is the age variable and t' is the year of first registration. This distribution evolves due to ageing, where vehicles gradually move to older age brackets, or with scrapages, where vehicles are taken out. The distribution tends to decrease to

zero as a increases, particularly beyond 25-30 years. This change can be expressed in terms of an age dependent probability of scrapage $p_i(a)$, which increases with age. Therefore the change in the distribution for one year of ageing is proportional to the existing distribution:

$$\Delta n_i(a, t') = -p_i(a) n_i(a, t') \Delta t' \Delta a. \tag{34}$$

This instantaneous probability of individual vehicles to be scrapped as they age translates to a probability of vehicles to survive up to a certain age, which is the solution to the previous differential equation:

$$n_i(a, t')\Delta t' = n_i(0, t')\ell_i(a)\Delta t', \ell_i(a) = \exp\left(-\int_0^a p_i(a')da'\right), \tag{35}$$

where $\ell_i(a)$ is the survival function, or the fraction vehicles of type i that survive up to age a. Assuming that the quality of the make of vehicles does not change with time (we see in section 4.2.3 that this has been the case in the UK), its life expectancy μ_i can be derived:

$$\tau_i = -\int_0^\infty a \frac{d\ell_i(a)}{da} da = \int_0^\infty \ell_i(a) da, \tag{36}$$

where the second integral is obtained by integrating the first by parts.

The survival function can be obtained in either of two ways, depending in which 'direction' one looks at the age distribution of a vehicle population, which is function time (t) and age (a). In age space at fixed time t, one notes from eq. 35 that

$$1) \quad \left.\frac{dn_i(a, t')}{da}\right|_t \Delta t' = n_i(0, t') \frac{d\ell_i(a)}{da} \Delta t', \tag{37}$$

and one looks how the distribution evolves between age brackets for a specific year of measurement. Here $n_i(0, t')$ corresponds to new registrations (the age zero population) at different years of make. Meanwhile, with age a fixed, one notes from eq. 34 that

$$2) \quad \left.\frac{dn_i(a, t')}{dt}\right|_a \Delta t' = n_i(a, t') p_i(a) \Delta t', \tag{38}$$

where one looks at how the distribution evolves in time t by comparing the change of population within specific fixed age brackets (e.g. specific years of make). Here $p_i(a)$ is the probability of making it to the next year given a certain age a.

In the first case, one can obtain the survival function $\ell_i(a)$ (or the probability of death $d\ell_i/da$) by dividing the distribution (or the age derivative of the distribution) for a particular year, e.g. 2011, by historical registrations, matching numbers and registrations by year of make. For instance, dividing out how many of the Citroen C3 2003 remain in 2011 by how many were initially registered in 2003. This procedure produces directly the survival function or its derivative but involves additional data, registrations of new vehicles.

In the second case, one looks at the change in numbers in each age bracket across years (e.g. 4 years old vehicles in 2011 compared to 3 years old vehicles in 2010, etc.). This then involves no additional data and yields $p_i(a)$, which has quite a different interpretation in comparison to $d\ell_i(a)/da$. Both analyses are done with UK data in section 4.2.3.

3.3.2. Application: emissions factors of the fleet vs new vehicles

As we show in the data section, it is easy to find out what are the emissions factors, or fuel efficiencies, of new vehicles currently in the market. However it is much more difficult to find out what the efficiency of the current fleet is, since it is composed of both old and new vehicles, and we expect that efficiencies may have improved over time. As we show below with data from the UK, for which we have the fortunate situation that a survey of the existing fleet has been carried out, this is effectively the case. The question arises then as to whether it is possible to work back fleet emissions from the distribution of emissions from newly purchased vehicles.

This is difficult and can only be done very approximately. Emissions within the fleet are distributed, due to a very wide range of vehicle models with different power ratings and engine sizes, but also due to vehicles of different ages, with older technologies having been engineered with other primary objectives than fuel efficiencies. To calculate average fleet emissions, it is important to include the relative amount of new and older vehicles in order to weight

the sum correctly. For instance, where numbers grow quickly as it does in China, the fleet is younger and average fleet emissions are closer to those of new vehicles than where numbers are stable, such as the UK. Using $E(a)$ as the average emissions from one age tranche of the fleet, average fleet emissions $\overline{E}_k$ of a particular world region k are:

$$\overline{E}_k = \sum_i \frac{N_{ik}}{N_{tot,k}} \frac{\int_0^\infty E_{ik}(a)\xi_{ik}(a)\ell_{ik}(a)da}{\int_0^\infty \xi_{ik}(a)\ell_{ik}(a)da} \tag{39}$$

We consider that technologies are produced by multinational car makers who apply the same technologies globally (international spillovers). Therefore, we do not assume that technology availability differs particularly significantly between regions, even though relative sales vary. However the rate of growth of car numbers does vary significantly between regions (e.g. China compared to the UK), making the ratio of new to old vehicles very different. We assume that international car makers standardise new fuel saving technologies in their models sold worldwide. For example, when a technology such as composite materials is developed, it eventually becomes applied to all models and is not subsequently removed unless it is superseded. This means that the time variation of $E(a)$ is not far from same globally, but that the efficiency of the fleet is determined by the relative numbers of vehicles with different emissions factors in different regions (e.g. vehicles with larger engines in the USA compared to the UK), $E_i(a) = E_i(0)f(a)$, where $f(a)$ is the relative variation of emission factors in time historically up to now, averaged across models. With this simplification the problem becomes separable:

$$\overline{E}_k = \sum_i \frac{N_{ik}}{N_{tot,k}} E_{ik}(0) \frac{\int_0^\infty f(a)\xi_{ik}(a)\ell_{ik}(a)da}{\int_0^\infty \xi_{ik}(a)\ell_{ik}(a)da} = \sum_i \frac{N_{ik}}{N_{tot,k}} E_{ik}(0)\Theta_{ik}. \tag{40}$$

Thus if $f(a)$ can be known for one region, by knowing the emission factors of new vehicles $E_{ik}(0)$ and the sales history by region, one can work back fleet emissions approximately for other regions. If furthermore emissions are dominated by petrol engines, we can furthermore approximate that the ratio emissions of old vehicles to new vehicles is technology independent, leading to

$$\overline{E}_k = E_k(0)\Theta_k. \tag{41}$$

Using the survey of the UK car fleet, an approximate function $f(a)$ was determined, as shown below in section 4.3.3.

3.3.3. Population dynamics

Out of survival analysis or technology demography, with additional arguments concerning allocation of new sales, it is possible to derive population dynamics identical to that of competing species in an ecosystem, in other words a Lotka-Volterra set of differential equations (LVEs), sometimes called 'Replicator dynamics', referred to in section 3.1, eq. 5. As opposed to many empirical works, the LVEs are not taken by assumption, they are derived from simple arguments of industrial dynamics, bandwagon effects and reliability theory, and its parameters have a meaning. This is done in detail in Mercure (2015), summarised here. This theory can be visualised in terms of flows of market shares between technology categories due to substitutions.

New vehicle purchases cover both replacements and increases in total population. Given that the global vehicle production capacity is large and that vehicles can be traded, we assume that sales are limited by the demand, not by the supply. Even in regions such as China, where growth is significant, sales leading to increases in population do not exceed sales for replacements, based on our data (section 5.2). Therefore the rate of increase of production capacity is small in comparison to global production capacity (see also Mercure, 2015, for a discussion of demand-led versus supply led assumptions in this population dynamics context).

During a time span Δt, out of a total $\xi_{tot}(t)$ of new registrations in a particular region, a certain fraction of sales is allocated to different technology categories according to consumer preferences F_{ij} as derived above, and replacement rates, denoted by $1/\tau_i$. These parameters can be understood as determining the rate of influx and out-flux of shares of sales in and out of technology categories i and j in a set of n possibilities. Using the variable N_i for the vehicle population in category i, increases in N_i due to purchases being allocated into i related to the replacement of vehicles scrapped in category j (i.e. substitutions of is for js at the time of scrappage) corresponds to:

$$\Delta N_{j\to i} = \begin{bmatrix} \text{Fraction of} \\ \text{prod. capacity} \\ \text{belonging to } i \end{bmatrix}_i \begin{bmatrix} \text{Consumer} \\ \text{preferences} \end{bmatrix}_{ij} \begin{bmatrix} \text{Fraction of} \\ \text{destructions} \\ \text{belonging to } j \end{bmatrix}_j \begin{bmatrix} \text{Number of} \\ \text{destructions} \end{bmatrix}_{tot}, \tag{42}$$

where destructions of vehicles in j are allocated to categories according preferences, which direct flows of units between categories. Meanwhile, the number of vehicles purchased that are not replacements are

$$\Delta N_i^{\uparrow} = \frac{1}{n}\sum_j^n \begin{bmatrix} \text{Fraction of} \\ \text{prod. capacity} \\ \text{belonging to } i \end{bmatrix}_i \begin{bmatrix} \text{Consumer} \\ \text{preferences} \end{bmatrix}_{ij} \begin{bmatrix} \text{Population} \\ \text{increase} \end{bmatrix}_{tot}. \tag{43}$$

The numbers of vehicles and vehicle destructions follow directly from the sum of the sales time series, multiplied with the survival function, over all ages (numbers), or its derivative (deaths), which correspond to convolutions.

$$N_j(t) = \int_0^\infty \xi_j(t-a)\ell_j(a)da, \quad \text{and} \quad d_j(t) = \int_0^\infty \xi_j(t-a)\frac{d\ell_j(a)}{dt}da \simeq \frac{N_i}{\tau_i}, \tag{44}$$

where d_i denotes deaths, a vehicle age and $\ell_j(a)$ the measured survival function for technology j. In a scheme where computational power minimisation is sought, deaths can be conveniently and safely approximated with the total population divided by the life expectancy, N_j/τ_j.[6] A question arises here as to whether the frequency at which vehicle choices take place is as slow as the life expectancy implies. We return to this question in section 3.3.4.

The production capacity by technology category changes through sales and re-invested income. We have shown that under particular forms of the survival function of the production capital, this can also be approximated as proportional to the current population, by category, divided by an industry specific growth rate t_i itself determined by the re-investment rate, the production efficiency and the survival rate of the production capital. This is a reminder that the production capacity established in an industry is built out of income made on selling units in the past, some of which may still be in use. Furthermore, a growing/declining production capacity is inseparable from growing/declining sales, such that a growing/declining population is associated with a growing/declining industry (see Mercure, 2015, for a detailed demonstration of this effect). Thus equations 42 and 43 are rewritten as

$$\Delta N_{j\to i} = \frac{N_i/t_i}{\sum_k N_k/t_k} F_{ij} \frac{N_j/\tau_j}{\sum_k N_k/\tau_k} \Delta N_{tot} = S_i \frac{\bar{t}\bar{\tau}}{t_i \tau_j} F_{ij} S_j \frac{N_{tot}}{\bar{\tau}} \Delta t, \tag{45}$$

where $\bar{t}$ and $\bar{\tau}$ are the average industry growth rate and life expectancy, while the S_i are technology category shares of the total fleet. For convenience we define the matrix of time constants $A_{ij} = \bar{t}\bar{\tau}/t_i\tau_j$. For all flow $\Delta N_{j\to i}$ of substitutions between i and j exists a reverse flow $\Delta N_{i\to j}$, and thus a net trend

$$\Delta N_{ij} = N_i \left(A_{ij}F_{ij} - A_{ji}F_{ji}\right) N_j \frac{N_{tot}^{\downarrow}}{\bar{\tau}}. \tag{46}$$

The growth of the fleet can also be expressed in a similar way:

$$\Delta N_i^{\uparrow} = \frac{1}{n}\sum_j^n \frac{N_i/t_i}{\sum_k N_k/t_k} F_{ij} \Delta N_{tot}^{\uparrow}, \tag{47}$$

where $\Delta N_{tot}^{\uparrow}$ is the time dependent population growth rate, in principle determined by the change in demand and capacity factor. We can combine both equations 45 and 47 in a convenient way, by considering expressing it in terms of shares of the total population by technology $S_i = N_i/N_{tot}$, instead of absolute numbers, which must involve a chain derivative:

$$\frac{dN_i}{dt} = N_{tot}\frac{dS_i}{dt} + S_i \frac{dN_{tot}}{dt}, \tag{48}$$

[6]Note that this implicitly assumes an exponential survival function with argument (half-life) τ_j. However changing the shape of the survival function for the same life expectancy changes this result very little, and thus results are not strongly dependent on shape, but rather more so on the life expectancy. Other shapes of the survival function, as measured in further sections, predominantly induce a sliding of N_j forwards in time, i.e. $N_j(t - t_0)$ where t_0 stems from the deviation from the exponential form. However for all practical purposes the convolution can really be safely approximated for N_i/τ_i.

the second term cancels with the equation for the population growth, leaving

$$\Delta S_{ij} = S_i \left(A_{ij} F_{ij} - A_{ji} F_{ji} \right) S_j \frac{\Delta t}{\tau}. \tag{49}$$

This equation expresses *exchanges of market shares* between technology categories i and j according to preferences and rates of replacement. Cumulating all gains or losses to technology i at the expense or profit of all other categories, we sum over j and obtain the *replicator dynamics* equation, or LVE (eq. 5)[7]:

$$\Delta S_i = \sum_j S_i \left(A_{ij} F_{ij} - A_{ji} F_{ji} \right) S_j \frac{\Delta t}{\tau}, \tag{50}$$

in which the net flow of shares is regulated by the product of the matrices $A_{ij} F_{ij}$ minus its transpose. While the matrix A_{ij} is interpreted to represent *industrial dynamics* and *reliability*, the matrix F_{ij} is interpreted to represent consumer choices according to our decision-making model, and thus they are completely independent. It is a standard representation of the process of *selection*, identically used in evolutionary biology and economics. This non-linear equation is extremely easy to implement computationally, encapsulating very compactly all relevant population dynamics. In FTT:Transport, t_i is assumed the same for all technologies, and thus we take $A_{ij} = 1/\tau_i$.

3.3.4. The frequency of decision-making

It is not exactly correct to assume that the frequency at which decisions are made matches the frequency of scrappage of vehicles. In fact, what *limits* the rate at which vehicle type decisions arise is tied to the rate at which purchasers of new vehicles go back to the new vehicle dealer. New vehicle buyers do not *always* keep vehicles until the end of their statistical lives. In fact, in many cases, they change vehicle every few years, selling them on to the second-hand market. The rate at which they do this is typically tied to the length of time for which they are contracted to pay for the vehicle, which is much shorter than the lifetime of the vehicle. Contract length are typically between 3 and 5 years while life expectancies are of around 11 years.

Here, we consider that second-hand markets are 'slave' to the new vehicle market, in that its composition is entirely constrained by what has been chosen in the new vehicle market in earlier years. Secondly, vehicles pass through the second-hand vehicle market until scrappage at a variable rate not solely determined by rates scrappage, but also by the rate of acquisition of new vehicles.

We thus use two timescales in the model, one for decision-making and one for vehicle survival. The time scale for vehicle survival, derived from the survival function, is used to calculate the size of the fleet. The purchasing rate is the one used in the replicator equation. This is substantiated by the simple fact that the replicator equation cannot match diffusion rates that we observe in historical data, in many countries and technologies, if we use the survival rate. It is clear from the data that new technologies can diffuse at a rate faster than what would be predicted by the survival rate. Note that these values are upper bounds; the rate of adoption is determined by the product of consumer preferences F_{ij} and the rate of decision making.

These assumptions are supported by our historical database (section 5.2), in which we observe rates of diffusion of new technologies that are substantially faster than what would be allowed by standard survival analysis using known scrappage rates. Purchasing timescales (or turnover rate) that enable to fit γ_i values for new technologies are of the order of 3-5 years, much shorter than the observed survival time of 11 years. Using purchasing times of 11 years simply does not allow fitting the γ_i values of most new technologies (using the method described in section 5.5). We conclude that this is an important factor to keep in consideration.

3.3.5. A theory of social influence in vehicle purchases

In this last part of the theory section, we show here briefly including social influence in a discrete choice model leads to the exact same replicator equation. This is explored in detail in Mercure (2016b), and only summarised here.

[7]Traditionally the LVEs are expressed in absolute numbers N_i while the replicator dynamics equation is expressed in relative numbers S_i. These are really equivalent, connected to one-another through the chain derivative eq. 48. See for example Hofbauer and Sigmund (1998).

Discrete choice models define a linear random utility model, in which the utility U_i^* associated to purchasing a particular type of vehicle i is expressed as a function of a number of variables V such as income, gender, distance travelled and so on, and regression parameters β and error ϵ,

$$U_i^* = \beta_i^1 V_i^1 + \beta_i^2 V_i^2 + \beta_i^3 V_i^3 + ... + \epsilon_i. \tag{51}$$

We look for the probability that option i is chosen over other options,

$$P(U > \max[U_1, U_2, U_3, ...U_n]) = P(U > U_1) P(U > U_2) ... P(U > U_n), \tag{52}$$

Following standard theory, this leads to the MNL

$$P_i = \frac{e^{\frac{U_i}{\sigma}}}{\sum_j e^{\frac{U_j}{\sigma}}}, \quad \sum_i P_i = 1. \tag{53}$$

Taking the probabilistic choice P_i as determining the shares of the market, this determines how the market evolves, in equilibrium, for changes in variables. This can be converted to a CES function, and therefore can be assumed the same as the optimal choice in an equilibrium consumer theory.

If, however, one takes vehicle shares S_i as a simplified proxy for social influence, then one obtains a model in which shares depend on themselves in a recursive way, and the model cannot be solved as an MNL. In fact, we show here compactly that if the random utility is function of vehicle shares, the representative agent cannot exist. This is easily explained: if agents choose according to the shares that they see in the market (their choice likelihood being higher for vehicles with higher shares), this can be interpreted as a group of agents each of which has a different set of knowledge over his/her options. Thus, there cannot be a representative agent.

If an agents k *finds value* V_i^4 *in purchasing vehicle i that other agents* ℓ *also consume*, then a term that links the utility between agents arises (see e.g. Durlauf and Ioannides, 2010):

$$U_i^*(k) = \beta_i^1 V_i^1(k) + \beta_i^2 V_i^2(k) + \beta_i^3 V_i^3(k) + ... + \alpha f\left(\sum_\ell \beta_i^4 V_i^4(k,\ell)\right) + \epsilon_i(k), \tag{54}$$

The probability of option i being chosen over other options is function of *weighted* sets of choices,

$$P(U > \max[U_1, U_2, ...U_n]) = P(U > U_1)^{S_1} P(U > U_2)^{S_2} ... P(U > U_n)^{S_n},$$

In this case, the solution is different to the MNL:

$$P_i = \frac{S_i e^{\frac{U_i}{\sigma}}}{\sum_k S_k e^{\frac{U_k}{\sigma}}}, \tag{55}$$

in which every option is weighted by its own shares. Preferences P_i are instantaneous, but purchases happen at a rate τ_i^{-1}, following consumer needs. We then take preferences as the rate of change of shares (as opposed to be equal to preferences, as it would be in an equilibrium model),

$$\frac{dS_i}{dt} = \frac{1}{\tau_i} \frac{S_i e^{\frac{U_i}{\sigma}}}{\sum_k S_k e^{\frac{U_k}{\sigma}}}. \tag{56}$$

This is a form of replicator dynamics. Adding to this some notions of survival analysis (see Mercure (2016b) for details), one can mathematically transform this to something useable for durable goods, which is the particular form of replicator equation that we use in FTT:Transport,

$$\frac{dS_i}{dt} = \sum_j S_i S_j \left(A_{ij} F_{ij} - A_{ji} F_{ji}\right), \tag{57}$$

In a more general model, if we assume the linear random utility function is function of the log of shares, with parameter α,

$$U_i^* = \beta_i^1 V_i^1 + \beta_i^2 V_i^2 + \beta_i^3 V_i^3 + ... + \alpha \log S_i + ... + \epsilon_i, \tag{58}$$

then we obtain the more general replicator equation

$$\frac{dS_i}{dt} = \sum_j S_i^{\frac{\alpha}{\sigma}} S_j^{\frac{\alpha}{\sigma}} \left(A_{ij} F_{ij} - A_{ji} F_{ji} \right), \tag{59}$$

in which the particular S-shape of the diffusion is scaled by the ratio of the strength of the social influence α with the heterogeneity σ. At this moment, due to a lack of data for the strength of social influence in each FTT:Transport region, the parameter is set to 1, and therefore, we use the replicator function given in eq. 57. Altering α does not radically change the results, but it changes the very particular shape of the diffusion profile. Finally, note that setting the social influence parameter α to zero yields the MNL, and thus, gives back the standard equilibrium consumer theory obtained when optimising at the system level. It follows that optimising at the system level is a result of assuming (1) perfect information, and (2) no social influence.

3.3.6. Coherence length in FTT

The coherence length of a function or signal is defined by the length, along an independent variable, over which data points are related to each other. In FTT, we have autocorrelation in time, which means that in time, states of the model are related to states of the model at earlier time. This is due to its path-dependence, as imposed by the replicator dynamics equation. Since the model has no foresight, it does not have forward autocorrelation. For example, in biological systems, populations at a certain time depends on what the population was at earlier times. However, as time goes by, this influence wanes as other more recent events become comparatively more influential.

The autocorrelation of a model can be measured using a expression of the form $g(t) = \sum_\tau f(t-\tau)f(t)$, where $f(t)$ is the signal. In FTT, the coherence length is of the order of 5-10 years, stemming primarily from purchasing rates (see section 3.3.4). This determines, for instance, the length in time over which the historical data has influence over the trajectory of diffusion. After 10 years, the influence of historical data has declined a factor of around 2. Changes in the historical data would imply changes in populations, which would have an important impact, in comparison to changes in cost data. This is why kick-start policies (defined in the main text) have an important impact on technological trajectories.

3.4. Integration to E3ME

The integration of FTT:Transport to E3ME is made through several variables. The vehicle and transport demand econometric equations are technically part of E3ME, not FTT, and interact directly with other variables such as income, prices and output (GDP). These are ways by which interaction takes place with the larger economic model; however these are not the most important. Since transport consumes a large fraction of world production of oil, and since the value of oil is high (oil enables all types of mobility, whether people or goods), any changes in the demand for oil has far reaching consequences for the global economy. FTT:Transport controls the major part of the oil demand in E3ME, which specifies an econometric equation for the demand of 'middle distillates' (petrol, diesel, kerosene, etc, excluding heavy oil), by 22 types of fuel users (see Mercure et al. (2017) for more details on the overall model), including road transport.

We split road transport between passenger and freight. The freight component is not at this stage developed in a specialised FTT model, unlike the passenger component, as this will be part of future work. We make the split based on estimates of fuel demand per tonne-km (tkm) using Kamakate and Schipper (2009), Liimatainen et al. (2014), which accounts to one third to half of fuel demand in most countries. We control freight emissions using biofuel mandates; as we later develop a dedicated FTT model for freight, we will include all current technologies (diesel, advanced diesel, CNG and electric) in various size classes using the same method. The passenger component is the main focus here, as we are interested in policies affecting road passenger. Total fuel used calculated in FTT:Transport originates from assumed emissions factors. However, actual fuel use is often more than what is rated by manufacturers, due to driver behaviour not exactly matching the standardised driving profile used by manufacturers for estimating emission factors. We find that total FTT fuel use in all countries accounts for around two thirds or more of fuel use obtained

from IEA statistics. Assuming that the error originates from these emissions factors, we scale FTT fuel use to match IEA values at the start of the simulation, and keep these scaling factors constant (one per region) until the end of the simulation.

Changes in fuel use have profound repercussions in E3ME, which we summarise here (more details can be found in Mercure et al. (2017)). Changes in the demand for oil for transport affect the price of oil through our fossil resources depletion algorithm (Mercure and Salas, 2013). This module determines the marginal cost of oil production based on a database of types of oil extraction and their cost (conventional oil, offshore, heavy oil, shale oil, tar sands etc). If the demand increases, more costly resources are developed and the marginal cost goes up, while if the demand declines, costly resources are abandoned and the marginal cost goes down. Oil and gas prices changes in E3ME are set proportional to changes in their marginal cost. When the price of oil changes in E3ME, it affects the oil & gas industries, which through multiplier effects (input-output tables) leads to changes in economic conditions that can be quite large. For example, it can leads to stranded fossil fuel assets and unemployment in fossil-fuel producing countries.

The demand for electricity from electric vehicles in FTT:Transport is also accounted for in E3ME, which is fed through to the sister model FTT:Power. It can affect for example the development of renewables or affect policies for decarbonisation if the demand increases as EVs diffuse to larger shares over time.

3.5. Policy in FTT:Transport

In this section, we review the representation of all policy types available in FTT:Transport. Eight types of policies are available to use independently, which we divide into two types: the policies that take the form of a pecuniary incentives that are applied at the time of vehicle purchase, and those that do not. The pecuniary incentives can be described by looking at the equation of the LCOT, while the other policies are not related to the LCOT, but rather, to share values. Note that all policies are defined by zero or non-zero values exogenously given between 2018 and 2050, with the exception of efficiency standards, which are currently set as a fixed target.

3.5.1. Pecuniary incentives

We reproduce here the LCOT, with added policy parameters. We use double letters to denote policies, while other symbols remain the same as in eq. 28:

$$LCOT_i = \sum_t \frac{\frac{I_i}{CF_i(t)} + VT_i + CT(\alpha_i) + \frac{FU_i(t)}{\beta_i} FT(\alpha_i, t) + \frac{MR_i}{FF_i} + RT_i(t)}{(1+r)^t} \Big/ \sum_t 1/(1+r)^t, \tag{60}$$

where,

- VT_i is a *registration vehicle tax or rebate*, in \$/veh, per vehicle type/class, paid at purchase time,
- $CT(\alpha_i)$ is a *registration tax based on the fuel economy* α_i, in \$/veh/(gCO$_2$/km), not type- or class-specific, paid at purchase time,
- $FT(\alpha_i, t)$ is a *tax on fuel consumption*, in \$/litre, paid each year, depending on the fuel economy α_i,
- $RT_i(t)$ is a *road tax*, vehicle type/class-specific, paid once per year.

These fall into two types: those that are paid once, and those that are paid yearly. The difference in impact is that the yearly policies are discounted, while the on-off policies are not. CT_i is a form of carbon tax, and so is FT_i; the difference however stems from the fact that CT_i is a tax on expected lifetime emissions, while FT_i is a tax on actual emissions. These imply slightly different outcomes, as CT tends to have a higher impact per dollar taxed than FT, simply due to time preference. VT_i is typically not used at the same time as CT, and their inclusion aims at enabling different types of strategies, where for instance VT_i can be made into a tax-feebate scheme in which tax income is recycled into subsidies for low-carbon vehicles. RT_i offers a similar counterpart to FT.

3.5.2. Regulatory and push policies

Many policies in the real world do not take the form of pecuniary incentives, and often do not apply at purchase time either. These can be of regulatory nature and apply to manufacturers. In FTT, this implies influencing the flow or value of shares in particular technology categories. For instance, it can involves exogenously changing the values of the preferences F_{ij} (see eq. 50).

Policies included are:

- *Regulations banning the sale of a technology type/class*. This involves setting $F_{ij} = 1$ and $F_{ji} = 0$. Existing vehicles of these types live to the end of their lifetimes.
- *Kick-start programme (public procurement)*. This policy exogenously changes the shares of a vehicle type/class at a specific point in time.
- *Biofuel mandates*. This exogenously determines the relative content of liquid fuels between fossil and renewables types, for all vehicles.
- *Efficiency standards*. This exogenously defines the efficiency of new internal combustion vehicles, per vehicle type/class.

We note that there is strong cross-policy interaction in FTT:Transport, as is the case in other FTT models (see e.g. Mercure et al., 2014). In particular, regulatory and pecuniary policies can enable each other's effectiveness. For example, setting up a kick-start programme in tandem with a fuel tax promotes the fast diffusion of low-share low-carbon technologies; acting faster than a tax alone.

3.5.3. Corresponding real-world policies

Real-world examples for each FTT policy exist, some of which are relatively common. We give some examples below:

- *Regulations banning the sale of a technology type/class*. UK Diesel car ban.
- *Kick-start programme (public procurement)*. China ten Cities, Thousand Vehicles Program; License plate quota for EV (430000 petrol cars quota and 170000 EV quota); China EV credit point; The Chinese government purchased. The Chinese government has directly purchased around 90000 EV in 2017 (see `https://www.ft.com/content/a55e7d36-db8a-11e5-a72f-1e7744c66818`).
- *Biofuel mandates*. Brazil National Alcohol Program and EU biofuel mandates.
- *Efficiency standards*. US CAFE; EU fuel economy standards; Japan Top-Runner program; China fuel economy limits.
- *Registration carbon tax* South Africa's registration carbon tax, equal to R100/gCO_2/km for each gCO_2/km above 120gCO_2/km, charged when a new car is purchased.

4. Deriving general information from the UK fleet

This part describes the procedure with which data that was collected for parameterising FTT:Transport. The data takes predominantly one of two forms: distribution of vehicle properties and vehicle numbers. While it is possible to obtain vehicle numbers regionally by technology type, this is not the case for vehicle properties, and in this case it is done for 18 representative regions, including Europe, USA, China, India, Japan and Brazil. We consider these regions to cover well enough most variations between car markets in the world that are of significance for global emissions, while maintaining our work manageable. Some of this works has been published already as Mercure and Lam (2015b).

The UK dataset for vehicle properties was used as a model against which we designed the data collection for the other regions. It is also the richest dataset of all, enabling to derive properties that cannot be obtained for other regions. Given this advantage, the UK dataset was analysed in great detail in order to derive a large amount of vehicle fleet properties. Where data is not available, whenever it makes empirical sense (e.g. in Europe), UK values are used in FTT:Transport. For example, the only survival function derived from actual data in this work is from the UK dataset, which is unlikely to be possible in other regions due to the lack of time-resolved survival data. We thus assume that the shape of the survival function is similar elsewhere. In sections beyond that for the UK, the methodology is the same unless stated otherwise. Similarly, the UK dataset enables to re-construct properties of the current fleet as opposed to more easy to access data about new vehicles. In particular, this enabled us to determine the distribution of current vehicle emissions factors as well as those for new vehicles entering the fleet, which are quite different, a difference that we extrapolated to other regions.

4.1. Procedure and datasets

Two sets of data for vehicle populations in the UK were used, along with datasets related to vehicle prices. These are, respectively, data from the vehicle registration agency DVLA for vehicles registered for the first time and existing registrations (DVLA, 2012a), DVLA observations of the vehicle population using cameras and number plate recognition (DVLA, 2012b), and data for new vehicles currently on the market (Car Pages, 2012) and motorcycles (Motorcycle News, 2012). The dataset for new registrations was matched with those of vehicle prices in order to derive price, engine size and emissions distributions for newly registered vehicles. Meanwhile, the dataset from the survey was used along with aggregate numbers of vehicles to derive properties relating to the current stock of vehicles, which differ from those of new vehicles.

The registration dataset provides the numbers of new registration entries for vehicles for years from between 2001 and 2012,[8] per vehicle model. For vehicles, this has a very large number of entries, around 30 000, as some models have numerous slightly different variants, and some filtering of entries was necessary. Filtering the entries with less than 100 new registrations, this narrowed down the list to less than 3000, more manageable, while still keeping the major part of the registrations. A large variation in model entry names resulted in many additional entries for vehicles with very similar features. Most of these correspond to special editions of other existing models.

The car price data from `www.carpages.co.uk` also had of order 2000 entries, each of which features a price value, a measure of emissions and an engine size. This was carefully matched by name, entry by entry, to those of the DVLA new registrations database. In this way, nearly 1.9 million vehicles were assigned a price, an engine size and rated emissions. The price data relates to new 2012 vehicles. Data was available in the DVLA set for new registrations in 2012, the year over which the data matching was done. Registration data is also available for previous years, however prices are not available historically. It is in principle possible to explore registrations in previous years matched to 2012 prices; however when going back several years, some of the models being sold back then do not exist on the market anymore in 2012, and therefore this produces an incomplete picture with missing models, having no counterpart in the price database. A similar procedure was followed to match motorbike registrations to the price database Motorcycle News (2012), using a filtering of entries with less than 10 registrations. Note that it took quite some time to develop this database, hence we use 2012 data. We may carry out an update shortly.

Meanwhile, the dataset for the survey of observations (DVLA, 2012b) provides vehicle features such as age, emissions, mass and engine size, for vehicles observed on the roads at 256 sites around the UK during years between 2007

[8] Document VEH0160 (2012)

and 2011. These observations were made using digital cameras on roads with automatic number plate recognition, and then matched with the DVLA registration database. This enabled to assign data from registration certificates to these observations. The aim of this exercise by the authorities was to find unlicensed vehicles on UK roads, but the data was released for research, removing personal information, as a statistical resource. This was performed and released for 5 years from 2007 to 2011. Each year features around 1.2 million observations, a number high enough to generate reliable distributions quite representative of the UK's operating vehicle stock, of about 28 million vehicles. The number of observations is however not proportional to the number of vehicles in the UK but varies over the years, and thus the distributions derived from these observations required to be rescaled to real population numbers to be comparable across years. For this, registration data for existing vehicles from the DVLA was used,[9] and thus all distributions produced for the existing population are scaled in fractions of the total population, not in terms of the number of observations. This was used to explore the evolution of the age distribution of UK vehicles, and to determine current distributions of emission factors, engine sizes and vehicles masses.

Further useful datasets exist from the DVLA. VEH0211 (2012) provides numbers of licensed vehicles by years since their first registration, i.e. by registration year. This therefore follows the gradual decline of vehicles in the fleet as they age year after year. We used this to evaluate the survival function of UK vehicles. Finally, document VEH0153 provides the longest time series of vehicles licensed for the first time (i.e. sales) in the UK, since 1954.

4.2. Distributions and heterogeneity

4.2.1. Overall new car price distribution

Figure 2, left, shows the overall price distribution of UK car sales in three different years matched to 2012 prices, calculated using ranges of £1000 of constant width in price. The distribution features a clear long tail in the upper price range (note that upper end of the price database ends with a Lamborghini at around £300k, beyond the scaling of this graph). We also observe that the shape of the distribution has not changed across years, which we interpret as evidence that purchasing behaviour has not changed significantly since 2008. In other words, a similar proportion of expensive or economic vehicles was purchased in 2012 compared to 2010 and 2008 (also in 2011 and 2009, not shown).[10]

The right panel of figure 2 displays a similar distribution but calculated using constant widths in the natural logarithm (base e) of the price of $\Delta \log_e P = 0.1$ (while the plot expresses spacing in base 10). With this we observe that the distributions are symmetric in log space, in other words the distributions are lognormal, as is generally the case for income distribution in most countries, suggesting that these may be related (Mercure and Lam, 2015b). We conclude from this that price comparisons are better performed in logarithmic space as opposed to linear. In other words, price ratios can be used; if one compares two prices P_1 and P_2, one evaluates $\log(P_2/P_1)$. The bottom panels show the distribution of engine sizes and emissions, which are equally widely distributed. Averages and standard deviations are given in the charts.

4.2.2. Price and emissions distributions of new vehicles by category

The FTT framework requires sub-divisions of engine type categories in order to feature a certain amount of resolution, and because different vehicle classes may evolve differently (i.e. the overall price distribution may change). As seen in the main text, this division is done in terms of engine size class. Vehicles were classified into three classes of engine power, which we term *economic* (Econ), *mid-range* (Mid) and *luxurious* (Lux), for each type of engine technology (petrol, diesel, hybrid and electric). These classes were defined for liquid fuel based technologies according to engine size ranges : < 1400cc, 1400cc to 2000cc, and ≥2000cc, based on the classification of Eurostat.[11] For electric vehicles, only three models were found in both UK databases, each of which conveniently happens to match a particular market segment, economic (Renault Twizzy), mid-range (Nissan Leaf) and luxurious (Tesla), and were thus assigned their own category. As can be observed in the chart, price distributions in each class are roughly similar between engine technology type. This supports defining FTT categories as such.

Price distributions by category are given in figure 3, and are observed to be unimodal, suggesting that this is an appropriate disaggregation. These can be used to evaluate consumer choices in the FTT binary logit decision

[9] Document VEH0205 (2012)

[10] Missing models in years earlier than 2012 are not related to their price, as far as we know, and therefore this conclusion can be drawn.

[11] This was done for convenience when processing vehicle number data for the EU, saving large amounts of time.

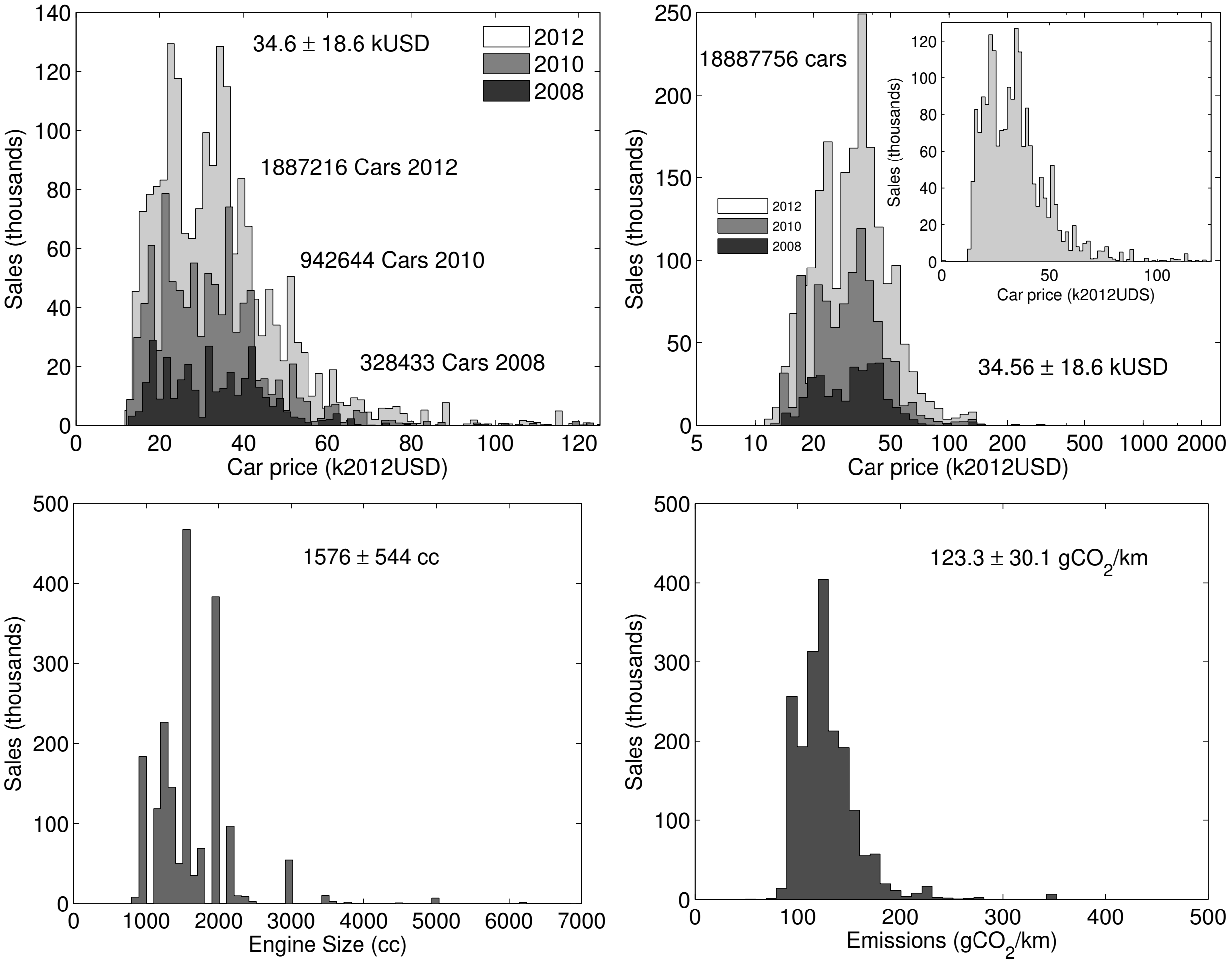

Figure 2: *Left* Price distribution of UK vehicles for three different years, based on 2012 prices, scaled linearly in prices, with constant spacing linearly in price. *Right* Same price distribution in logarithmic price space, with constant spacing in the logarithm of the price.

framework, as distributed investment terms in the LCOT. The standard deviations are also used and represent consumer and technology diversity. Note that for the UK, CNG and LPG engines are rather rare and were left out of the analysis, even though they are be present in other nations.

Emission factors vary significantly between engine class and technology, and therefore must be determined accurately in order to track changes in in total emissions as the technology composition changes. This is given in figure 4. Electric vehicles are considered zero emissions at the tailpipe, electricity emissions being counted elsewhere.

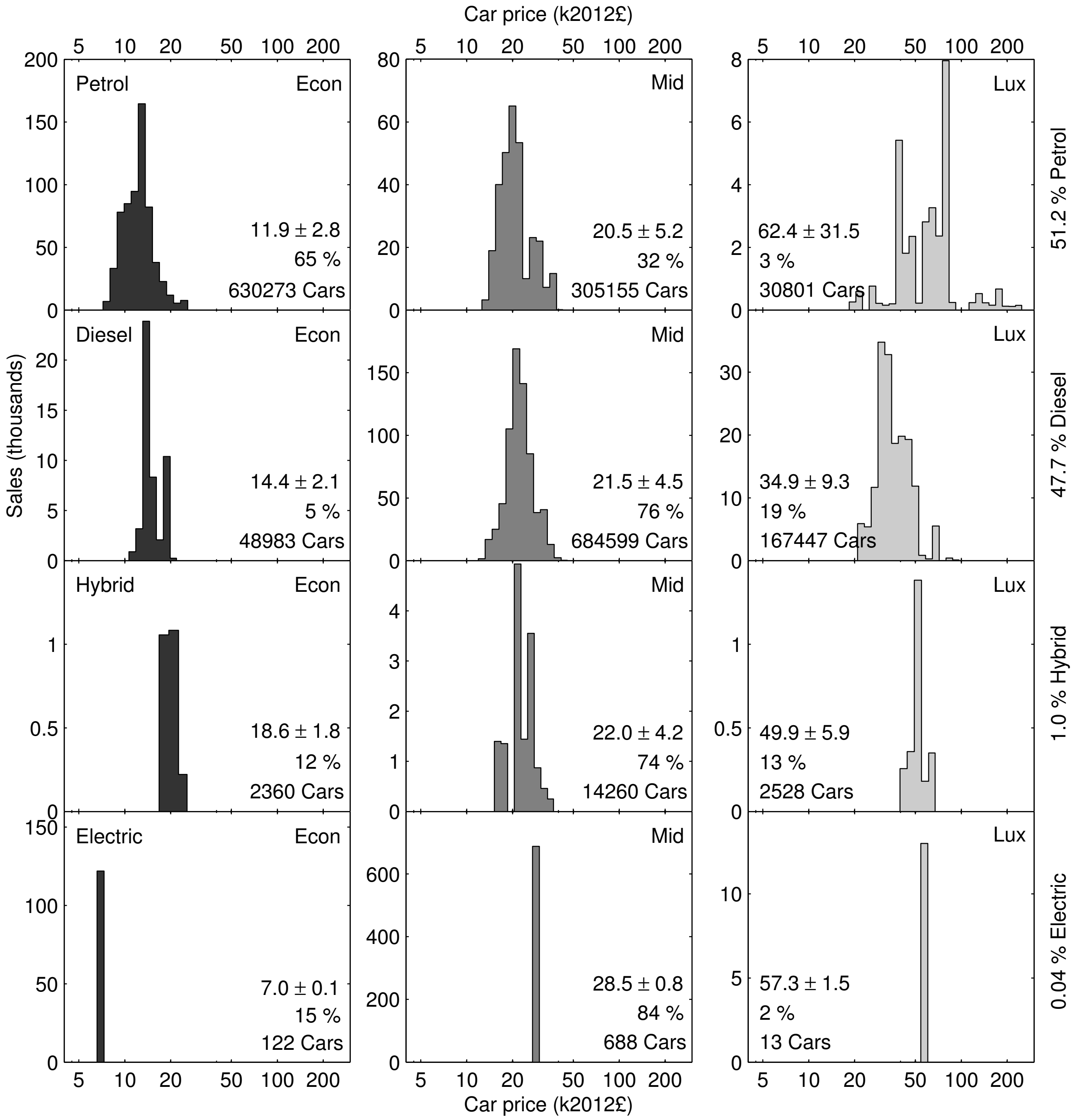

Figure 3: Price distributions of vehicles matched in the database for three classes (economic, mid-range and luxurious) of engine power by type of engine technology. Percentage values indicate the share of each power class in a technology type. Few instances of electric vehicles were observed in the database, and since the number of available models is restricted, they only have one price value for each class and thus cannot strictly speaking be considered distributions.

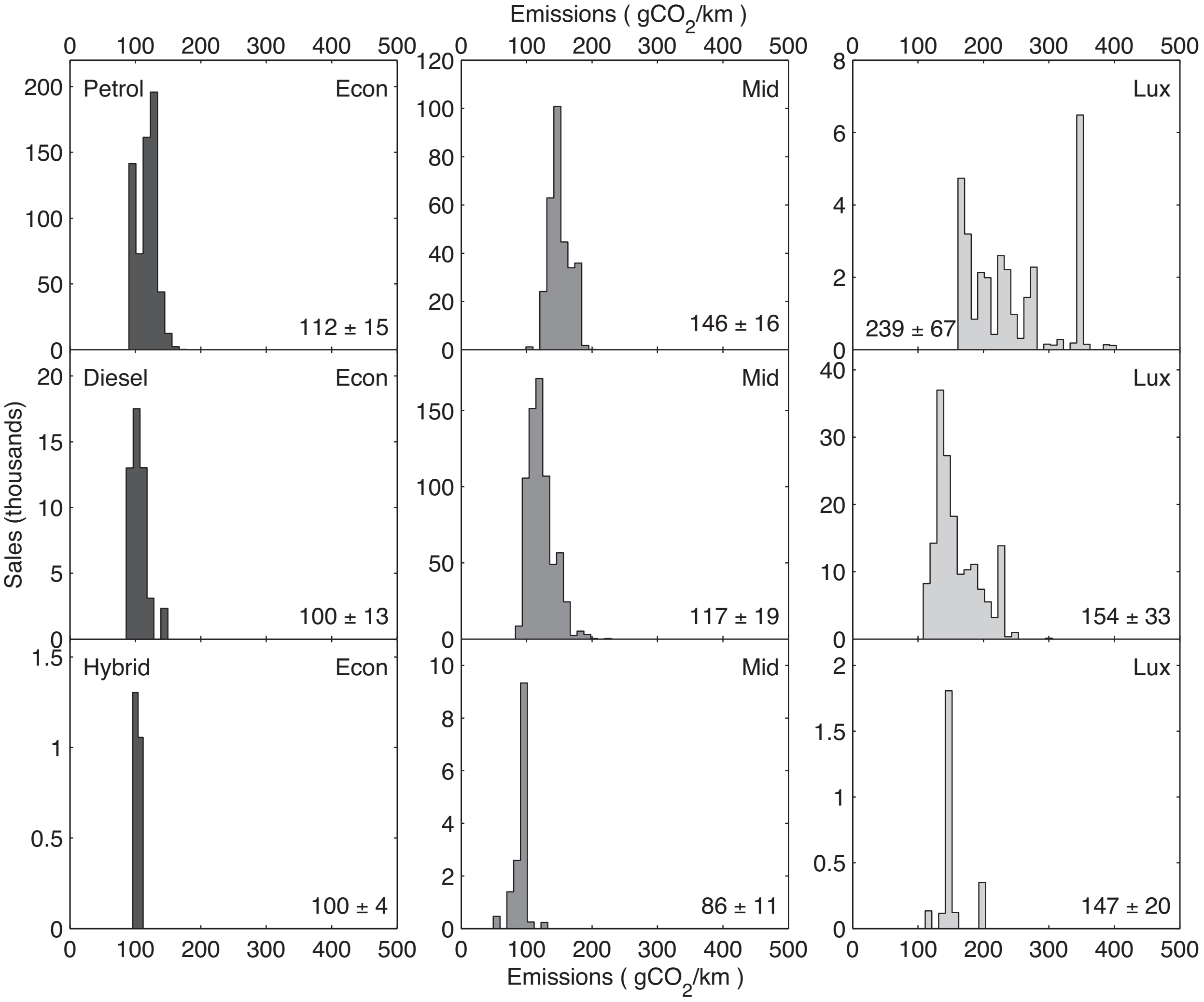

Figure 4: Emission factors by engine type and class. Numbers in the charts represent averages and standard deviations.

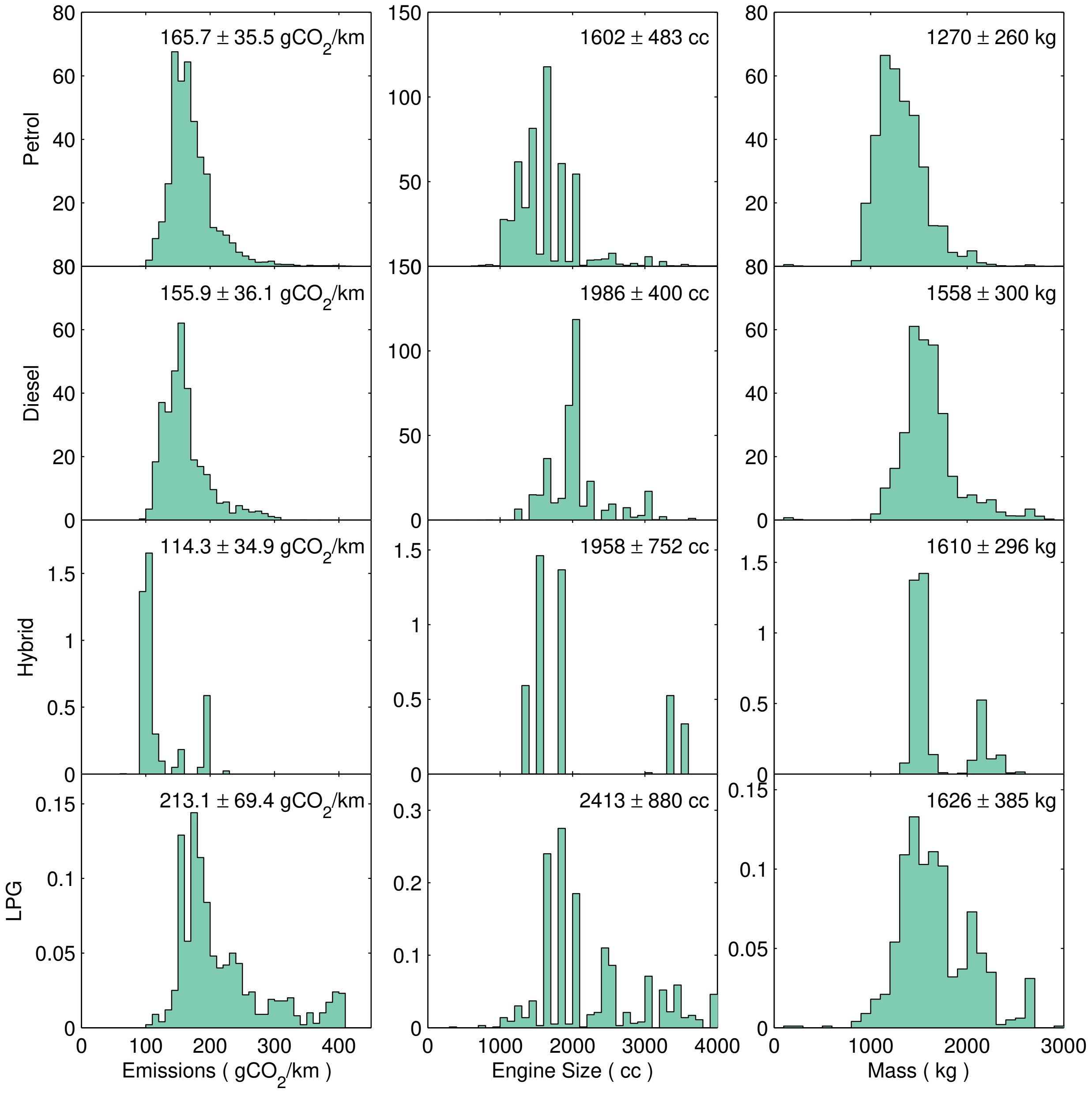

Figure 5: General properties of the UK car fleet by technology type with size classes combined.

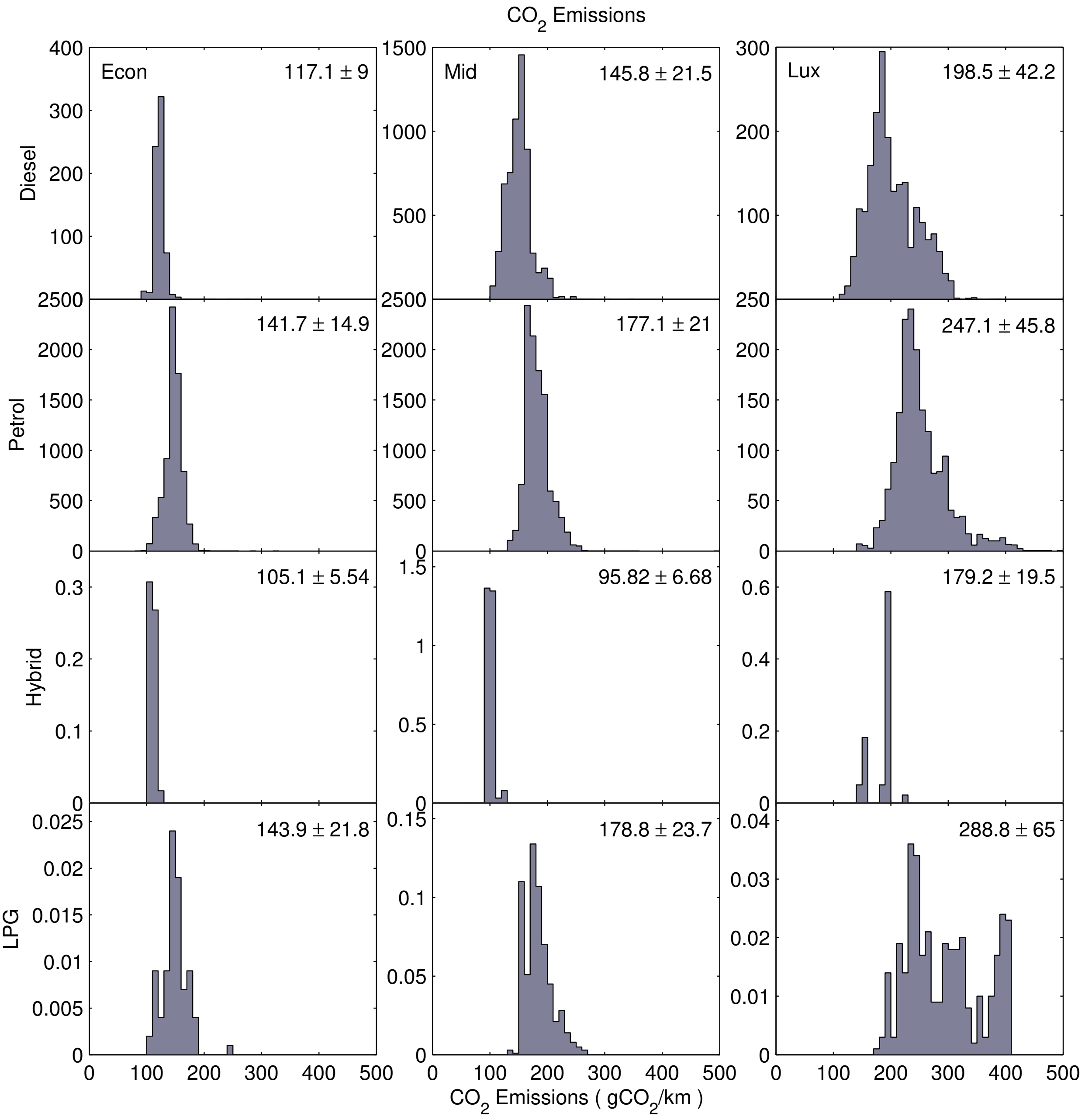

Figure 6: Emissions by engine type and size class.

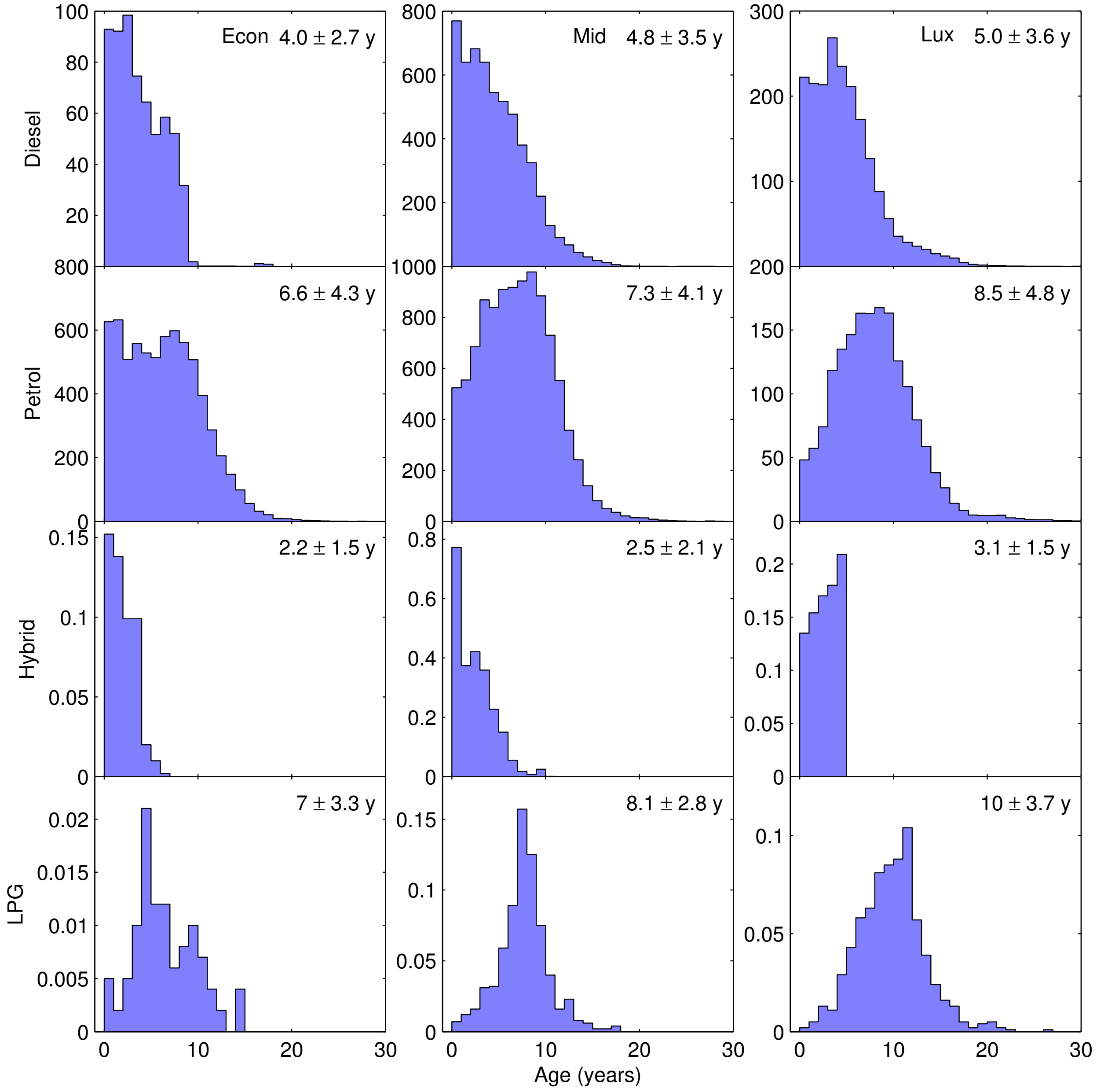

Figure 7: Age distributions by engine type and size class.

4.2.3. *Distribution of emissions, engine size and vehicle mass for the current fleet*

Figure 5 shows a summary of the profile of the current car fleet in the UK. Using the survey dataset of vehicle observations with number plate recognition, we reconstructed a current snapshot picture of emission factors, engine sizes, age and mass composition of the current UK car fleet. This is extremely important for our model, since the previous dataset on car sales only provides information on new vehicles, and the properties of the fleet can differ significantly from those of new vehicles. This is particularly crucial for emissions factors, and this model's goal is to project future transport-related emissions.

Figure 5 shows distributions of engine sizes, emissions factors and vehicle masses, with mean and standard deviation values given in each panel, for both petrol and diesel vehicles. Mean values as well as standard deviation values are given in each panel, calculated from the data shown in the panel. This helps understand how the current fleet is composed. This is further subdivided in our usual three classes in the following section for more details. Note that LPG vehicles (converted petrol engines) have come in vogue for some years but their sales have declined again in recent years and thus do not appear in sales data.

4.2.4. *Distributions by size class for the current fleet*

Figure 6 gives emission factor distributions for all classes and technologies (excluding electric engines) for existing vehicles in the UK. Averages and standard deviations are given in each panel, and these are values used in the FTT model. The distributions in this figure are much better defined and narrow than when looking at the whole fleet, reflecting again that the chosen subdivision between size classes is very important. In particular, the emission factors of the Econ and Mid classes are very narrowly defined. The distribution of emissions for the Lux range is broader, reflecting that this range caters for a range of people with widely differing but high levels of wealth. Emission factors of current vehicles are approximately 20% larger than those of new vehicles. We model and explain this in section 4.3.3 using age distributed data.

4.3. *Vehicle lifetimes and survival*

4.3.1. *Age distribution of vehicles by category and survival functions*

Figures 7 shows age distributions by technology type and size class. Their shapes vary significantly between technology types, a representation that some technologies are established since longer than others, hybrid vehicles having been introduced very recently, and diesel vehicles displaying a change in the rate of adoption some years back. Meanwhile petrol vehicles have the expected age distribution decreasing with age following the survival function, and LPG vehicles have seen a past popularity that has waned to the extent that nearly no new LPG vehicles are seen anymore in the fleet. If we can assume that the survival function is a constant of time, these distributions essentially correspond to a product of registrations with the survival function along the age variable. We obtained these age distributions for five consecutive years of observations, from 2007 to 2011. They are clearly not constant, and this is a reflection that sales and registrations have been changing with time. A time series for car registrations can be obtained from DVLA document VEH0153.

4.3.2. *Survival analysis*

By calculating how many vehicles disappear between years of observation within particular registration year groups, it is in principle to determine the force of death and survival function, as described in the theory given in section 3.3.1. This was calculated for every engine type and size class, and re-averaged either over observations years or size class. Results are given in fig. 8. The resulting survival functions have a peculiar shape with a dip in early years, indicating that vehicle use may be age-dependent.

The DVLA provides a document where the decline of car numbers by year of manufacture are followed. This is shown in fig. 12, top left panel, normalised by the registration number. For each year of manufacture, a similar ageing pattern is observed. These can be shown to be very close to logistic functions of one displays them, using F the fraction of initial sales remaining, and plotting $F/(1-F)$ on a logarithmic y axis. This produces linear trends (not shown here). These curves can be overlaid over each other by displaying them according to age. All of these curves trace small sections of a longer trend which represents the survival function, shown in the top right panel.

The deviation of the survey data from this survival function has an explanation. For this we used the data in a different way than what was done in fig. 8. Taking the number of observations as a function of year of observation and

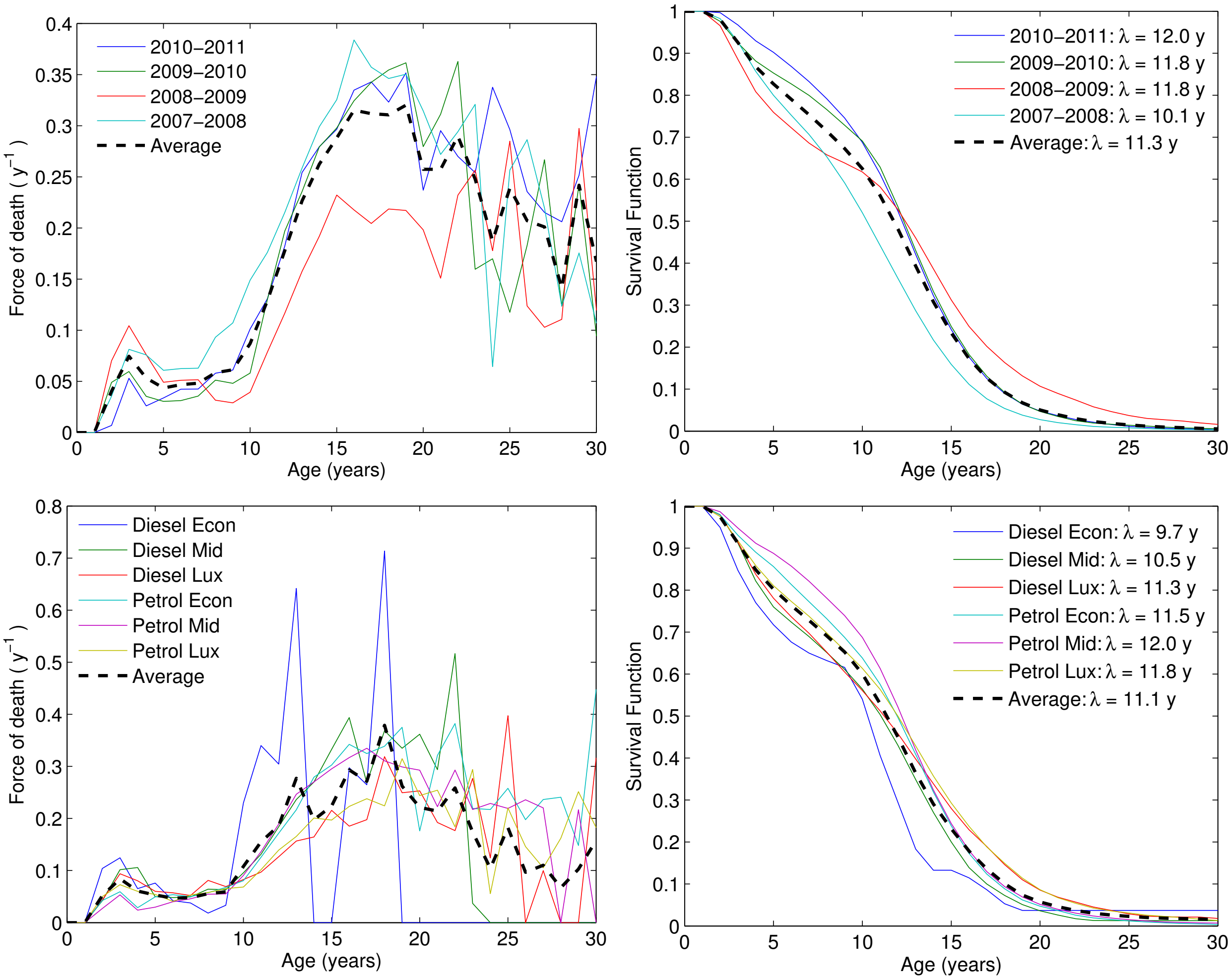

Figure 8: Survival functions as calculated using equations of section 3.3.1 with survey plate recognition data.

age, one can draw a three dimensional surface (fig. 12, middle left panel). If one follows on this surface trajectories followed by specific age groups, a gradual decline is observed along the time of measurement. If normalised by the initial number of registrations, such trajectories generate sections of the survival function at different ages, 5 data points long, assuming that the survival function has not changed significantly with time. Fractions of vehicles remaining from initial registrations for registration years since 1970 are shown in the middle right panel, with DVLA car registrations in the inset. Note that this fraction increases above one in recent years, apparently contradictory. The same data is shown in terms of age in the bottom left panel, with the same registration data in the inset. Overlaid with this is the survival function taken from the top right panel. We see that above 10 years of age, the survey data agrees very well with DVLA registration data, but that for younger ages the survey data climbs above one. This is interpreted to the fact that vehicles younger than 10 years of age are used significantly more than older vehicles, and approximately linearly so the younger they are. In the survey data, an arbitrary observation frequency arises that could be age-dependent, and this seems to be the case. Thus in the survey data, younger vehicles are over-represented because they are driven more often; meanwhile above 10 years old their frequency of observation in cameras is representative of their registration numbers. The bottom right panel shows the difference between the survey data and the survival curve.

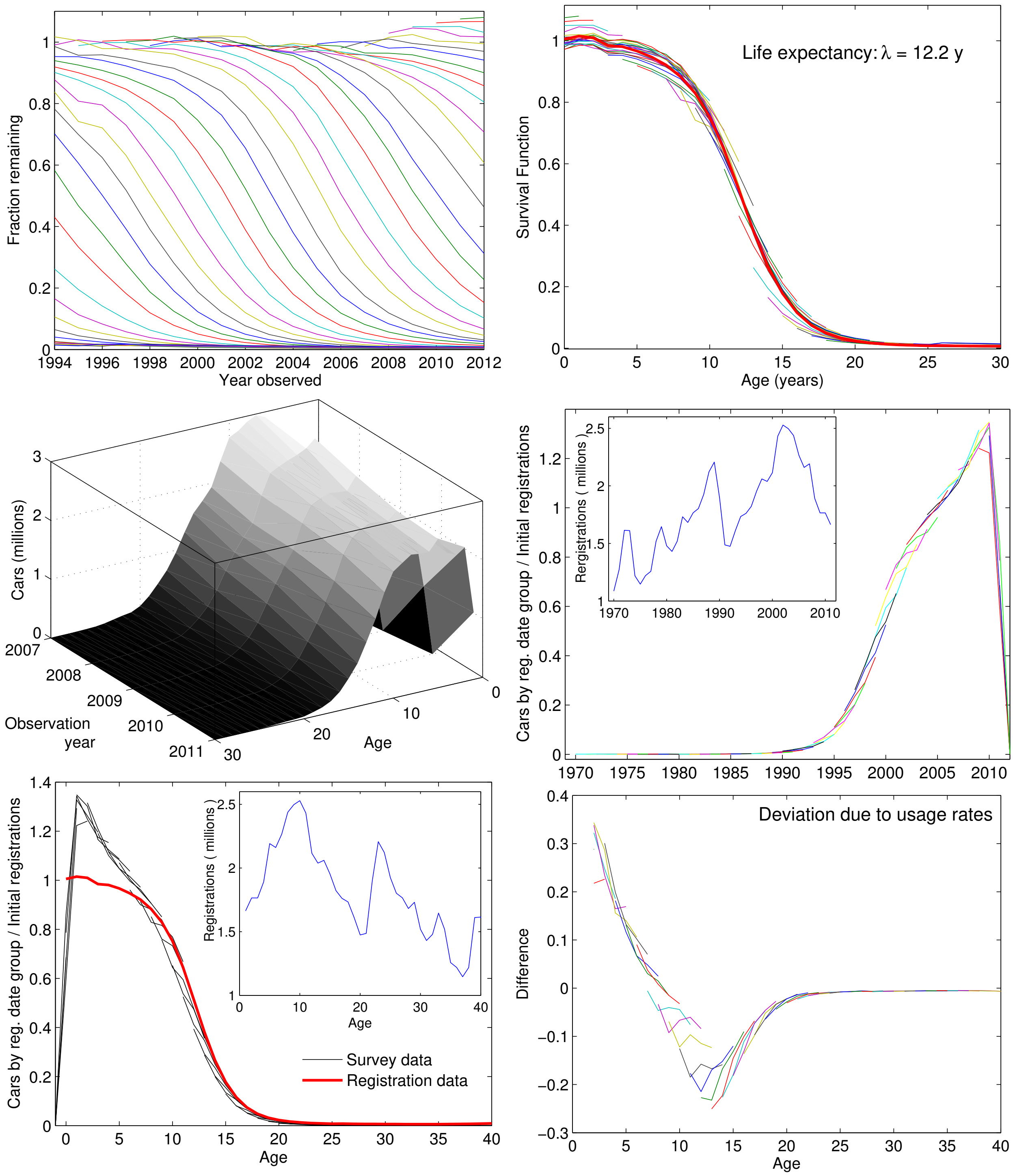

Figure 9: *Top two panels* DVLA registration data document VEH0211. *Bottom panels* Survival rates calculated using survey plate recognition data: young vehicles are over represented.

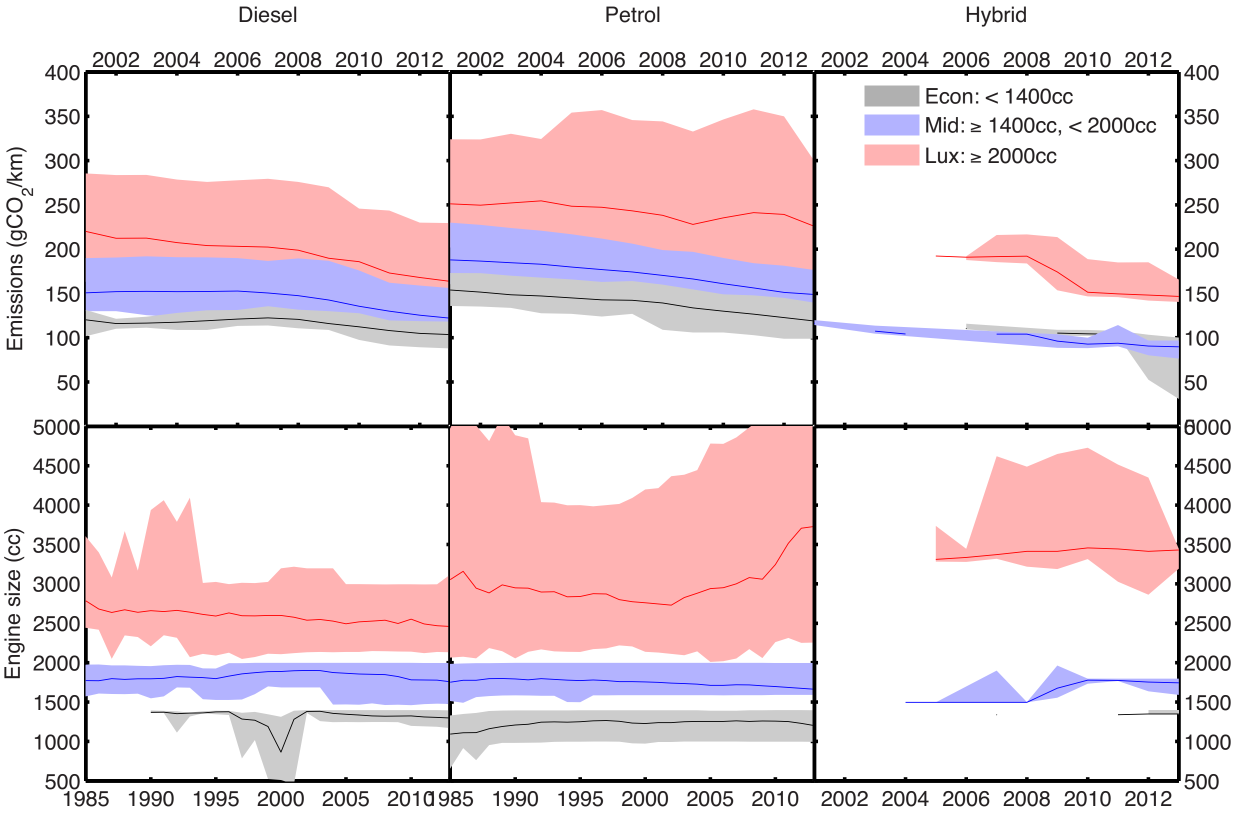

Figure 10: Changes of engine sizes and emissions factors over time for three engine size classes and three technologies against year of registration. vehicles registered prior to 2001 do not have rated emissions in the database. The shaded areas include 67% of all vehicles by category (the standard deviation).

4.3.3. Emissions factors changing over time

Section 3.3.2 describes how to determine fleet emissions factors from those of new vehicles using sales time series in different regions if the evolution of emission factors can be obtained for at least one country. The UK survey dataset offers this possibility, without which it might not have been possible. By determining the function $f(a)$ of relative changes in emissions in time, one can in principle have an idea how emissions of new vehicles have changed over the years, and use it to evaluate fleet emissions from a time series of sales and a survival function. If we assume that to first order, $f(a)$ is the same worldwide, then we can work back average fleet emissions by category from those of new vehicles.

In the the UK survey, each entry feature a year of make, an engine size and an emissions factor if the year of make is after 2000. We have analysed how emissions factors and engine sizes are distributed by engine type and size class and year of make. This is shown in figure 10, where lines indicate the mean of the distributions and the shaded areas the regions within their standard deviation. One observes that emissions factors have clearly gone down with time in each category, while engine sizes have not changed significantly. This indicates improvements in combustion technologies, not a change in the power of vehicles. We also observe wide variations in these characteristics for petrol and diesel engines, which are established and diverse, while hybrid vehicles feature a small number of available models.

Using these average trends, the function $f(a)$ can be determined for each category, shown in figure 11, by normalising mean emissions by their 2011 value. This thus indicates how emissions factors of new vehicles have changed relatively since 2001. These curves are noisy but a weighted average is given with the thick black curve. The grey shaded area indicates the 95% confidence level (two standard deviations). Using these numbers and the time series for

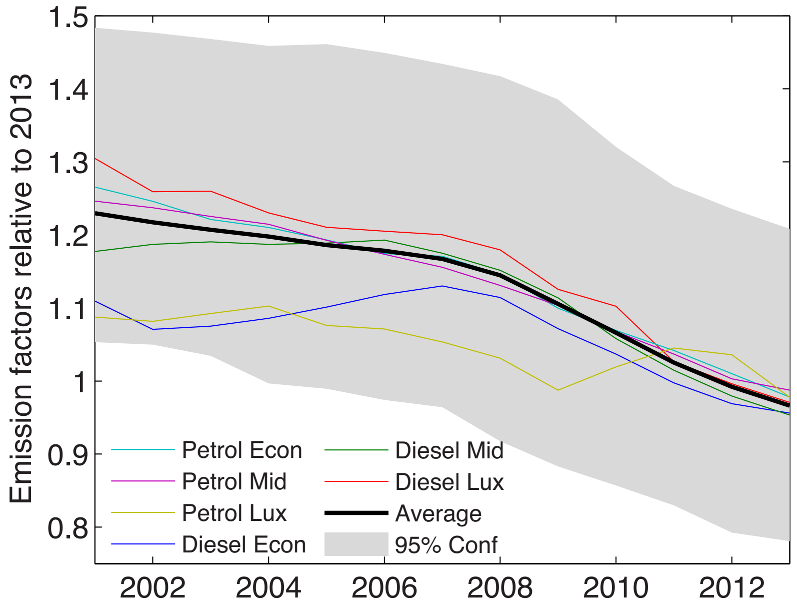

Figure 11: Trends of emissions factors normalised by their 2011 value (mean curves in the top panel). Solid lines are averages weighted by population numbers. Shaded areas indicate regions of parameter space in which 95% of the vehicles are located.

total registrations of new vehicles given in DVLA document VEH0153 and the survival function calculated above (see figure 12, bottom left panel), we evaluated fleet emissions relative to those of new vehicles using the function $f(a)$, which is taken as the average given in figure 11. This can be compared against measured fleet emissions from the survey, given above, in order to check whether this methodology works. Using the sum $\sum f(a)\xi_i(a)\ell_i(a)/\sum \xi_i(a)\ell_i(a)$, we obtained that fleet emissions are 15% higher than those of new vehicles, with an uncertainty range that spans from 10 to 20%. Meanwhile, comparing figure 6 to figure 4, we had established that fleet emissions were in general 20% higher than those of new vehicles entering the fleet. We conclude that this method works but only very approximately. The error stems from working with averages. It is, however, the only method available to us to evaluate fleet emissions in other countries than the UK.

4.4. Motorcycles

4.4.1. Motorcycle age distributions and survival functions

A survival function was calculated for motorcycles in the UK, which turn out quite different than that for vehicles, and hence it is useful to explore in detail. Following the same methodology as for vehicles, we used the age distribution by years from the DVLA survey data, as well as DVLA registration data from document VEH0311. In exactly the same way as for vehicles, the survey data produces survival functions that include an age dependent level of use of motorcycles, while the registration data does not.

Obtaining survival functions from registration data using document VEH0311, normalised by first registrations, is as straightforward as for vehicles, and this is shown in the top two panels of fig. 12. For every year of registration, the slope of the number of vehicles remaining with time shortly after first registration is very high, and slows down with age (left panel). By displaying the data in terms of age, and averaging, we obtain an average survival function (right panel). This shows that the survival function has not changed significantly with time and is fairly consistent. The average is shown with a thick red line, featuring a life expectancy of 7.3 years. The sharp decline at low ages is ascribed to high rates of accidents for recently purchased motorcycles. This slows down with age, where for old vehicles, breakdowns dominate and those that survive through an accident dominated young age often survive up to 20 years or more. Thus the distribution of ages of death is wider than for vehicles, the latter being dominated by breakdowns.

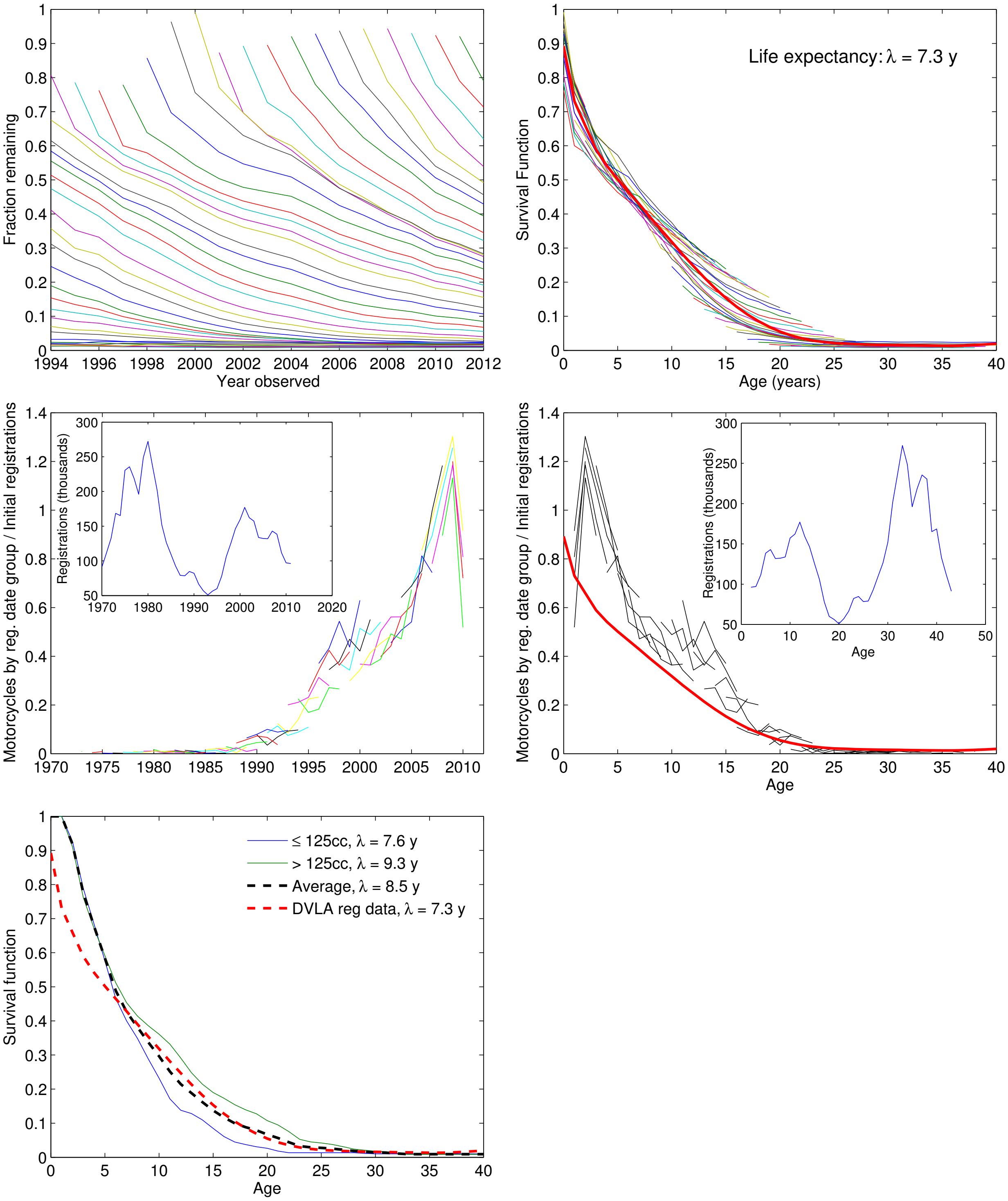

Figure 12: *Top two panels* DVLA registration data document VEH0311. *Middle panels* Survival rates calculated using survey plate recognition data. *Bottom panels* Survival functions calculated using survey data and equation 38, for large and small motorcycles, their average, and the survival function calculated in the top panels.

The survey data was used to determine declines in motorcycle populations by year of registration. This is shown in the middle panels (with historical registrations in the inset). This features an age dependent level of use. The survival function calculated in the top panels is overlaid to this data with a thick red line. We thus observe very rough consistency, and that the age dependent level of use is higher at young ages. Finally, survival functions were calculated from the survey data using eq. refeq:Survival2 as was done for vehicles. This produces survival functions again roughly consistent albeit with higher life expectancy by one year on average, with 1.7 years difference between small and large motorcycles. The difference between calculated survival functions most likely stems from an age dependent level of use, similarly for vehicles.

5. Data parameterisation of FTT:Transport

For the present study, an original database detailing the technological profile of vehicles and vehicle populations was built. Table 2 shows the data that were used to build everything needed to parameterise FTT:Transport. Columns two to four are give the scope and level of detail required, including the selected time periods and the resolution of the data. The following sections explain how each type of data was collected, and shows the data when possible. The rest of the FTT parameterisation can be obtained from the Supplementary Excel File that can be downloaded with this publication.

Table 2: Data sources for the main variables

Variable	Year	Differentiated in 18 countries?	Data source
Vehicle sales by model	2012	Yes	Fleet numbers are collected from Marklines
Vehicle prices	2012	Yes	Car manufacturer's website and car sales website (see details in table 4,table 5 and table 6)
Fuel cost	2013	Yes	Fuel use data are collected from car manufacturer's website and fuel price per litre is collected from the World Bank
Fuel economy	2013	Yes	Car manufacturer's website and car sales website (see details in table 4,table 5 and table 6)
Discount rate	2013	No	E.g. Harrison et al. (2010), Inderwildi and King (2012), Zhuang et al. (2007)
Learning rate	2012	No	E.g. AEA (2010), IEA (2013), McDowall (2012), Weiss et al. (2012)
Income data	1990-2012.	Yes	E3ME
Fleet size	1990-2012	Yes	Euromonitor
Historical fuel prices	1990-2012	Yes.	E3ME (IEA)

5.1. Cross-sectional data gathering procedure and distributions for 18 regions and proxies

This section provides an overview of new vehicle prices and emissions data we have collected. In FTT, all cost data comes in the form of distributions. This required to find data per vehicle model: number of sales, price, emissions, engine size. Such a database either does not exist for most countries, or would be too expensive to purchase. It was possible, however, to obtain vehicle sales per individual model from Marklines in one bundle for many countries. We verified that the totals match national data. The other characteristics of vehicles could be found on various commercial websites, including for instance www.carpages.co.uk, and manufacturer websites. We matched name by name the data for each individual vehicle model. This is quite time-consuming, but provides a very robust dataset. Since this took some time, the data dates back to 2012. We do not expect that the shape of these distributions change substantially over time, since they are mostly related to income distributions. The full methodology is explained in Mercure and Lam (2015b), and more details are given in tables 4, 5 and 6 discussed in section sect:General.

Figures 13, 14 and 15 shows our vehicle prices, emissions and engine size distributions for 18 countries. Sales of alternative technologies, hybrid and electric vehicles are shown in pink and red respectively, where available, scaled by factors indicated to make them visible, as their numbers are often orders of magnitude lower. We provide average and median prices, emissions and engine sizes with their standard deviation in Table 3. We observed that the USA has the largest average engine sizes, and hence the largest emissions, while India has the smallest average engine sizes, and hence the smallest average emissions out of all the countries.

Table 3: The average, median and standard deviations for car prices, engine sizes and emissions in the six representative regions.

Country	P(USD)			Engine sizes (cc)			Emissions (gCO_2/km		
	Average	Median	S.D.	Average	Median	S.D	Average	Median	S.D
USA	25959	23871	10570	3026	2550	1225	186	176	50
UK	34285	31520	18640	1576	1498	544	123.3	118.8	30
Japan	18317	14968	11526	1286	1252	728	113	102	44
China	22826	18970	16633	1704	1596	481	154	153	31
India	8674	6947	12418	1220	1170	445	140	145	27
Brazil	20642	16425	13770	1527	1558	458	112	106	29
Korea	19949	15432	13799	1840	1998	652	171	168	38
Argentina	20850	18720	11578	1646	1598	350	172	167	50
Australia	31948	28415	19029	2284	1998	863	127	123	34
Canada	21407	20914	10680	2857	2500	1285	210	206	60
Indonesia	11805	15654	10021	1630	1495	440	171	176	43
Malaysia	29976	14970	39277	1714	1586	628	183	181	47
Mexico	16630	13138	9253	2002	1800	779	168	171	57
New Zealand	52481	29611	72191	2280	2000	592	193	186	26
Russia	23560	23325	10690	1656	385	1600	188	190	54
South Africa	19976	1598	601	1869	1598	601	173	161	84
Taiwan	22922	21318	3250	1818	1798	247	146	160	64
Saudi Arabia	27027	22086	21600	2378	1180	2000	200	174	59

The diagrams give an overview of the diversity of markets in different nations, and are different everywhere. Mercure and Lam (2015b) shows that in the UK, where our data is most detailed, this distribution is roughly proportional to the income distribution (see section 4.2). Although we don't demonstrate it, this is probably the case in all countries (down to a cutoff value below which people do not purchase new vehicles). We stress that these exclude second-hand markets. For example, the price distribution in Japan is much narrower compared to China and the USA. Similarly, there is a clear difference between engine sizes and fuel economy between countries, implying that it is essential to consider different fuel economy and engine sizes for individual countries. For instance, in Saudi Arabia, the distribution of engine sizes covers 1000cc to 6000cc, while in Taiwan engine sizes are concentrated between 1500cc to 2800cc. In terms of emissions distributions, vehicles in the US have the highest emissions, while Japan has the lowest emissions as shown in table 3.

In terms of alternative technologies, price distributions are very different across countries. For instance, the UK has a wide range of hybrid vehicles and Japan has the highest penetration of hybrid vehicles. Notice that in Japan, Australia and Canada, the market for hybrid vehicles and EV is mostly concentrated in the lower engine class segments, while in the developing nations such as Indonesia, there are very few hybrid vehicles and EVs on the road. Some of this data was published by Mercure and Lam (2015b).

Following the method shown for the UK in section 4.2, these distributions were segmented into three distributions for each engine size class and technology type. For each, a mean and STD is taken in log scaling, since these distributions are highly assymmetric and roughly lognormal. We do not show this here, but the mean and STD values are given in the Supplementary Excel File.

5.2. *Construction of historical time-series*

5.2.1. *Market shares time-series*

To derive the γ_i values, one requires historical market shares for different vehicle technologies. To do this, we have either obtained the shares directly from Eurostat or derived the shares using historical sales of new vehicles and their survival rates. The market shares for petrol and diesel vehicles of different engine sizes for the EU countries are available from the Eurostat website [12] from 2002 onwards.

[12] http://ec.europa.eu/eurostat/statistics-explained/index.php/

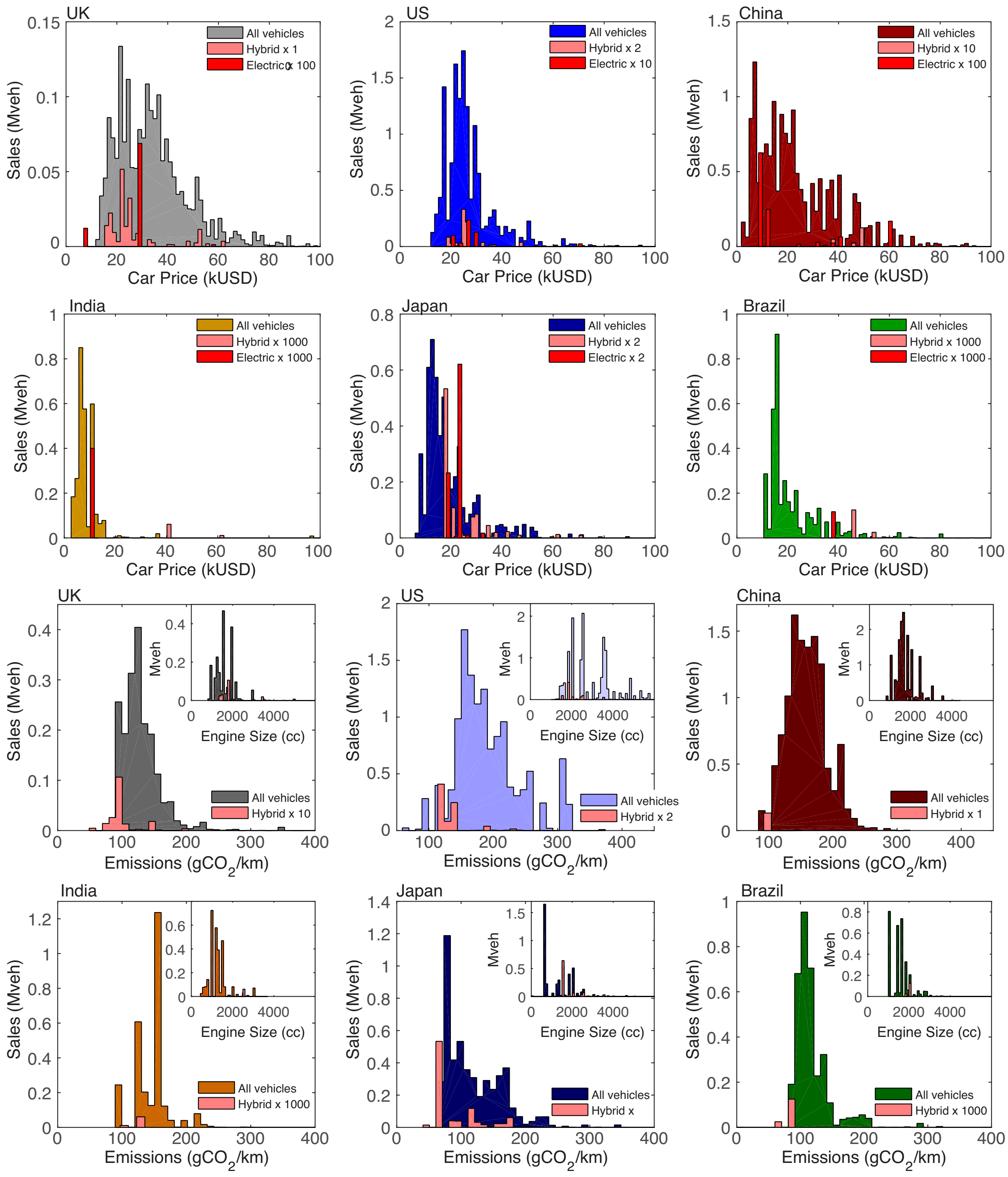

Figure 13: Price and emissions distributions for the UK, US, Japan, China, India and Brazil. Top two rows: price distributions for the six countries with identical price scaling.The price distributions for alternative vehicles are shown in pink. Bottom tow rows: emissions distributions (main graphs) and engine size (insets) of 2012 vehicles sales for the six countries. The emissions and engine size distributions are shown in pink (Mercure and Lam, 2015b).

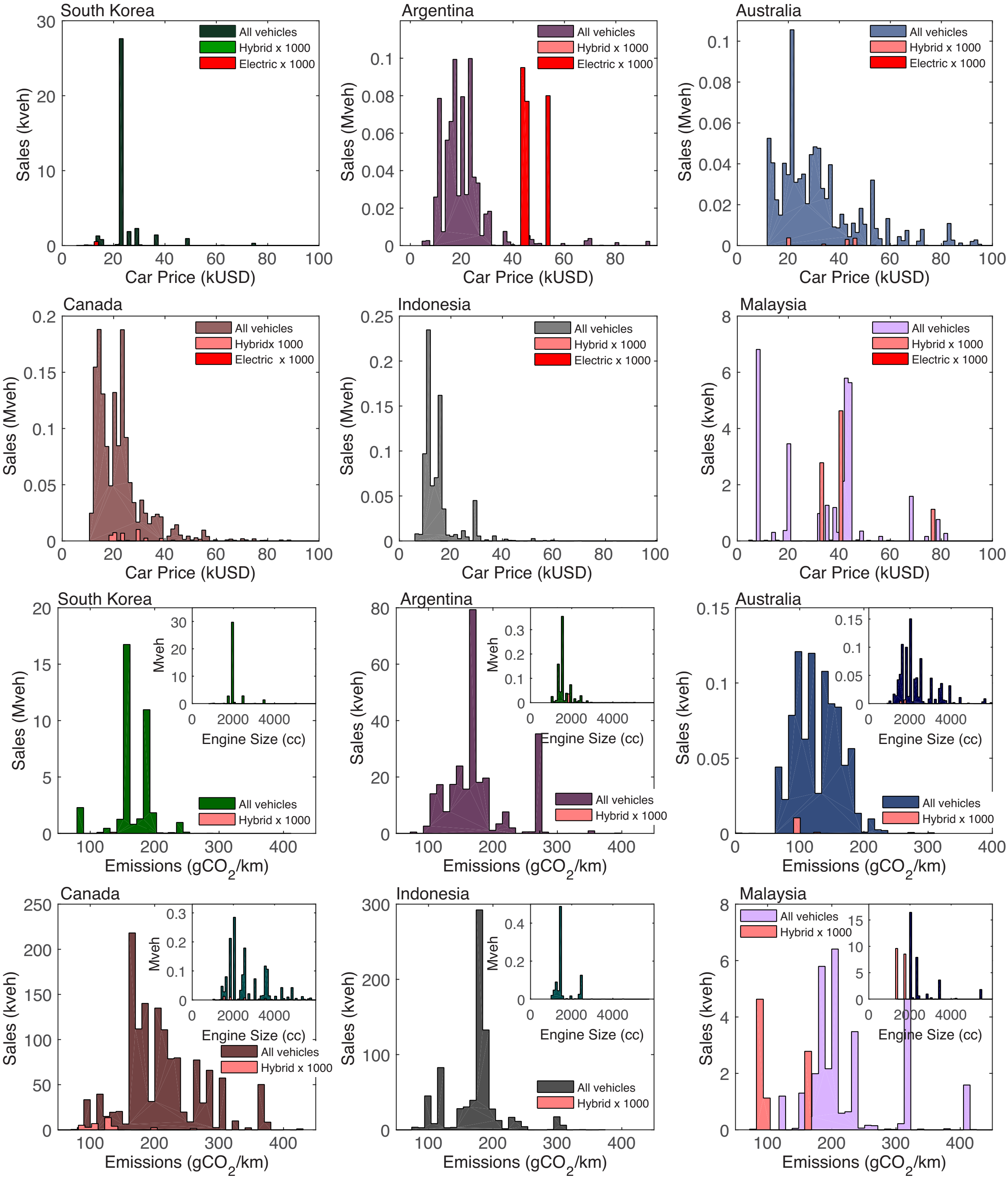

Figure 14: Price and emissions distributions for the Korea, Argentina, Australia, Canada, Indonesia and Malaysia. Top two rows: price distributions for the six countries with identical price scaling.The price distributions for alternative vehicles are shown in pink. Bottom two rows: emissions distributions (main graphs) and engine size (insets) of 2012 vehicles sales for the six countries. The emissions and engine size distributions are shown in pink.

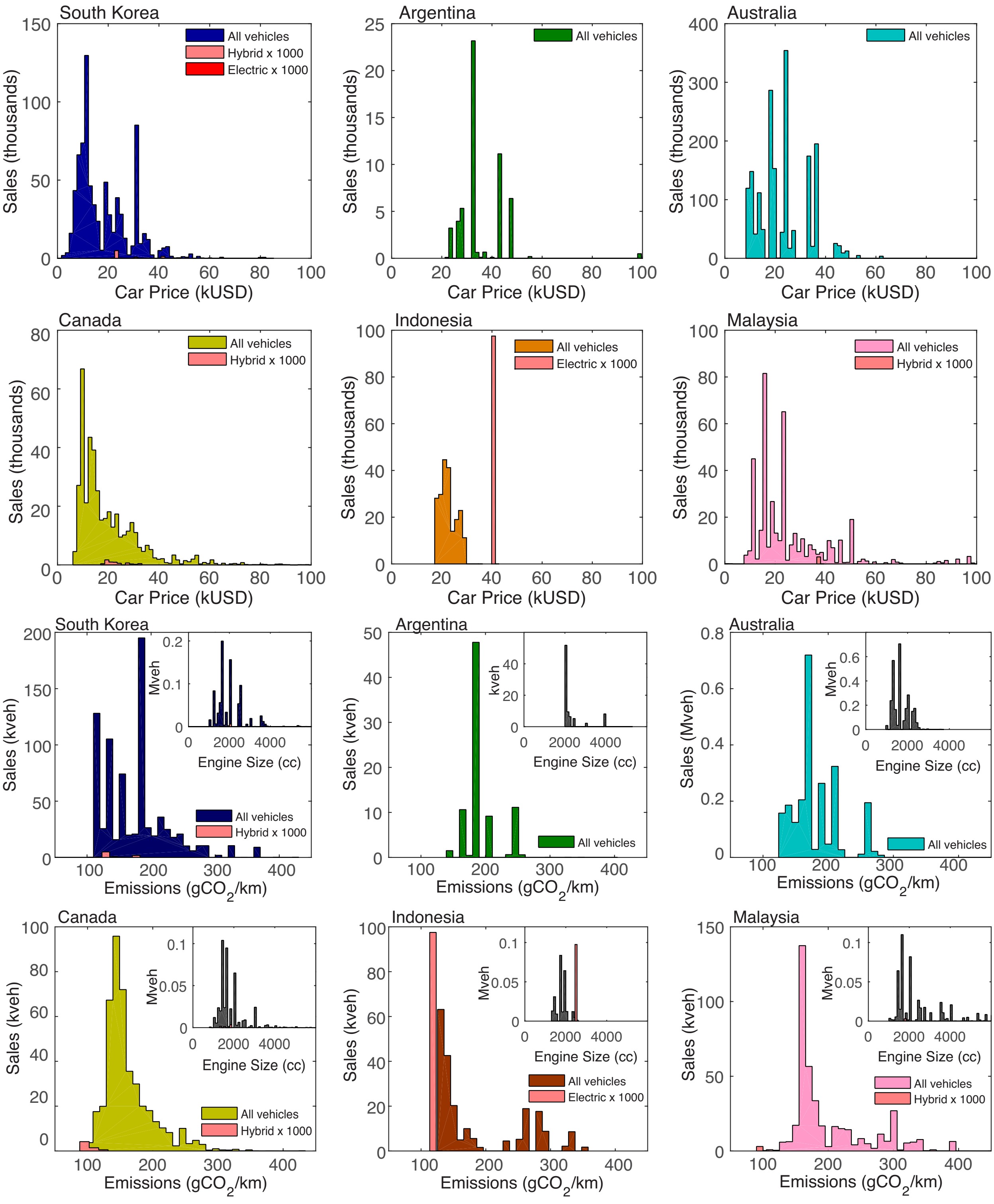

Figure 15: Price and emissions distributions for Mexico, New Zealand, Russia, South Africa, Taiwan and Saudi Arabia. Top two rows: price distributions for the six countries with identical price scaling. The price distributions for alternative vehicles are shown in pink. Bottom two rows: emissions distributions (main graphs) and engine size (insets) of 2012 vehicles sales for the six countries. The emissions and engine size distributions are shown in pink.

Outside the EU countries, market shares are not readily available. In particular, it is challenging to find market shares data for engine sizes that match the Eurostat engine size classification for petrol and diesel vehicles. The shares for these vehicles were calculated based on historical car sales from Marklines convolved with the survival function with the following equation:

$$N(t) = \sum_a \xi(t-a)\ell(a), \tag{61}$$

where $N(t)$ is the number of vehicles, $\xi(t)$ is the number of sales at year t, and $\ell(a)$ is the survival ratio of vehicles at age a. The survival function gives the fraction of vehicles that survive up to a certain age. It is typically represented as a monotonically decreasing function that declines from 1 to 0 as age increases. For example, in Zachariadis et al. (1995) the survival rates were simulated using a Weibull distribution,

$$f(x) = e^{-(\frac{x+b}{T})^b}, \tag{62}$$

where T parameterises the vehicle lifetime and b is the parameter that affects the shape of the survival function. Here, we did not parameterise a function but instead did the calculation directly with data (see Fig. 16).

We have purchased annual vehicle sales data from the Marklines website in the year 2013 and 2014, which is an automotive industry portal that consists of motor vehicles market data. Marklines provides for the total sales by model and brand for 63 countries from 2004 onwards. Hence, it is possible to know the sales for each model name for individual countries. Marklines sales numbers were checked for reliability against total sales given by a number of data sources (including official data published by the transport departments of various nations). We concluded that the total sales number in the Marklines dataset is consistent with the official data. This implies that the Marklines data cover all the models available on sale for each country, making our historical shares data reliable. We obtained in this way shares between 2004 and 2012, sufficient to determine γ_i values wherever shares are not zero. Since we cover every individual vehicle model, our data is very accurate even when numbers are very small. For example, orders of magnitude differences in sales are observed between petrol and EV vehicles, but that does not make γ_i values necessarily less accurate for EVs, unless numbers are very small or the non-zero time series is shorter than 5 years.

5.2.2. *Survival function*

The determination of vehicle survival rates requires substantial historical information on the stocks and scrappage. Three approaches could be used to find the survival function. Firstly, survival functions for some countries (e.g. China, Japan) have been found directly from existing literature (figure 16, see Goel et al. (2013), Hao et al. (2011)). Secondly, when the survival function is not readily available, we could derive a survival function by generating a survival profile based on the survival function derived for the UK (using data obtained from DVLA (2012b)). This is based on the assumption that the reliability functions of mechanical systems for vehicles are similar between countries for the first few years of their lifetime. This is because reliability of vehicles is not necessarily related to political borders, since most firms sell internationally. The difference in survival function between countries is related to weather and traffic context. This assumption is largely consistent with existing empirical evidence (Huo and Wang, 2012). Then, we constantly adjusted the survival values until the difference between total sales and total stocks (available from Euromonitor International (2012) for many countries) became approximately equal (figure 16, bottom left).

When neither total sales nor stocks were available (i.e. Indian motorcycles), we have to borrow survival functions derived from other countries. The variation in survival patterns of vehicles between countries can be attributed mainly to the difference in scrappage policies in different countries, vehicle management and improved technologies. For instance, China has mandatory scrappage standards for vehicles and motorcycles (e.g. 13 years for motorcycles). The scrappage policy causes a shorter average life expectancy for Chinese motorcycles than those in the UK (figure 16). Survival patterns should be more similar between countries sharing similar scrappage policies. For motorcycles in India, as for those in the UK, there is no scrappage scheme. While it could be argued that technology is more advanced in the UK than in India, vehicle makers are largely multinational, so technology spillovers almost certainly occur. Thus, the UK motorcycle survival patterns should give a fairly good approximation for Indian motorcycles (see figure 16, bottom right). A sensitivity analysis will be carried out to examine the effect of the uncertainties introduced by survival function approximation.

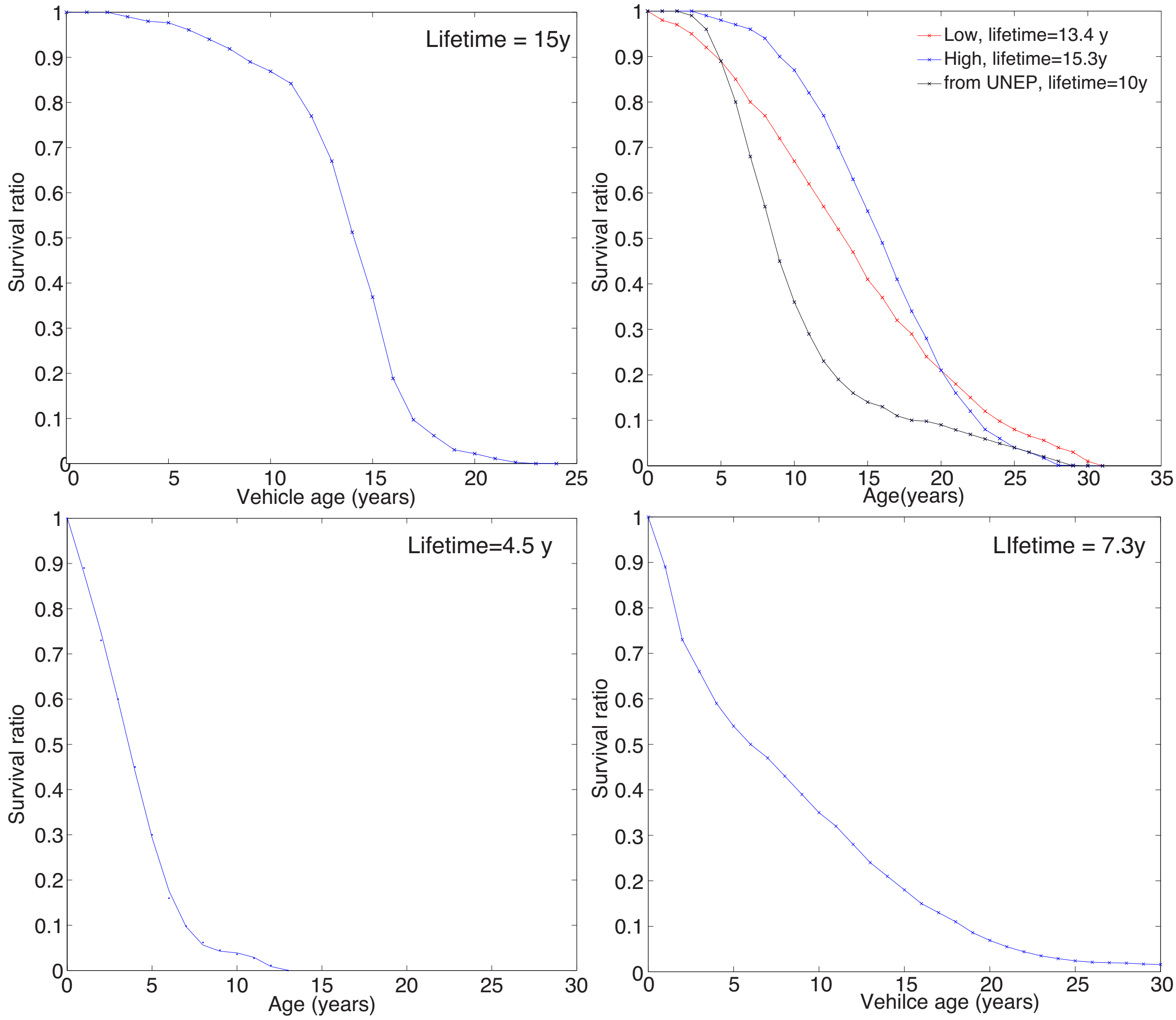

Figure 16: *Top Left* Survival function for vehicles in China. Data from Hao et al. (2011) *Top Right* Survival functions for vehicles in India. Data from Goel et al. (2013) *Bottom Left* Survival functions for motorcycles, adjusted for China. *Bottom right* UK survival function for motorcycles derived from the DVLA (2012b).

5.3. General parameters

As shown in tables 4, 5 and 6, car prices, engine sizes and fuel economy data for each car model listed in Marklines are collected from various sources, including car manufacturers, car sales websites, car industry market reports and government institutions, matched to the car models listed in the Marklines data. Note that the prices obtained are the listed price in the year 2013 when the data were collected. Car fuel economy data were collected from the manufacturers' websites when available. In some cases (such as Taiwan), it was much faster to obtain the car specification and prices from one single car research website where these data were readily available. To ensure the reliability of the data outside the manufacturer's website, we checked the price, engine sizes and fuel economy data from these car sales, research websites and government institutions against the data obtained from the manufacturers.

In cases where the fuel economy data were not available (e.g. Argentina), we used other countries as a proxy for the fuel economy data in similar markets. This is a reasonable assumption since similar car models are shipped/supplied to a number of Latin American countries. We also borrowed the price data across similar countries in cases where the price data were not available. For instance, in the case of South Korea, the prices for certain car models cannot be obtained from the official manufacturer's website in South Korea, and hence, we borrowed the Japanese car prices. Since we have matched the sales of the outdated models where data are not available readily from the car manufacturers, it is possible to look up the specification from the second-hand market without obtaining the car prices. Note that we have taken the car prices before the addition of any taxation.

In many cases, each car model has several car price and fuel economy values, depending on vehicle options for a car model. We usually take the mid-value for prices and engine sizes, unless it is known to us that a particular vehicle option/alternative is very popular. The data sources are listed in 4,table 5 and table 6.

Table 7 shows the full E3ME-FTT classification. For countries outside the 18 countries above, table 8 shows the country we have used as a proxy. For instance, we have used the price, engine sizes and emissions data collected for the UK as a proxy for all European countries. Our approach is based on the fact that it is the relative prices for vehicles within a country that matters for consumers when they are choosing vehicles, instead of the absolute price for vehicles [13]. Although there could be price diversity between countries, EU countries are regulated by the same emissions standards. While in Latin America, we have taken the Brazilian data for the Latin American countries such as Colombia. This is justified by the fact that similar vehicles in different countries in Latin America have been selling vehicles produced by the same manufacturers. In the case of countries defined as a group (e.g. ASEAN, OPEC), we have picked a major country as representative for the organisation. As shown in table 8, Malaysia is taken as a representative country for ASEAN and Saudi as a representative country for OPEC. Part of the data (the data for US, UK, Japan, China and India) is published in the Mercure and Lam (2015b), with the data collected by the authors.

5.4. Econometrics for transport demand and vehicle sales

Transport demand depends on the needs of the economy and preferences of people in society. Passenger transport demand can be measured in different ways, including the number of trips, vehicle kilometres and passenger kilometres. Two factors are particularly important in projecting emissions, namely the number of vehicles registered to be on the road and the number of passenger kilometres, which measure the quantity and volume of travel required by future passenger vehicles.

The future demand for transport is related to economic activity (GDP), fuel costs and demand elasticities. In the present model, by regressing the vehicle travel demand (pkm) with respect to income and fuel prices, the model predicts the vehicle travel demand up to 2050. Notice that the limitation for the regression is that it omits variables that are potentially useful in predicting vehicle travel demand, such as the public transport network, congestion, geographic area etc., due to the difficulty of finding sufficient data for 59 regions over a 20-year period. However, this would not be picked-up substantially well in the econometric specifications since many of these omitted parameters are not likely to change rapidly over time, while the econometric equations model changes. Equation 63 and equation 64 show the demand for passenger kilometres and the number of new vehicle sales, used by the E3ME-FTT model:

$$PM_j = \alpha_{1,j} * RRPD_j + \alpha_{2,j} * PFRM_j + \epsilon_j \tag{63}$$

[13] We note that for this study it was more important to focus on developing countries rather than on Europe for a global model. We plan to improve the data resolution for Europe in the future.

Table 4: Summary of data sources

Country	Car price	Engine size	fuel economy
UK	Car prices are collected from http://www.carpages.co.uk/ for both new models and outdated models.	The car engine sizes are collected from http://www.carpages.co.uk/, along with car prices and fuel economy.	The fuel economy for vehicles is collected from http://www.carpages.co.uk/, along with car prices and car engine sizes.
USA	Official websites of car manufacturers in the US for the existing models. For the old/outdated models, the price data were obtained from car dealers such as http://www.autonews.com/section/prices	Official websites of car manufacturers in the US. For the outdated/old models, engine size data were obtained from car dealer such as http://www.autonews.com/section/prices	Official websites of car manufacturers in the US. For the outdated/old models, fuel economy data were excluded from the calculation.
Japan	Official websites of car manufacturers in Japan for the existing models. The price data for vehicles sold historically were obtained from http://toyota.jp/service/dealer/	Official websites of car manufacturers in Japan for the existing models. The engine size data for vehicles sold historically were obtained from http://toyota.jp/service/dealer/spt , along with the price data and the fuel economy.	Official websites of car manufacturers in Japan for the existing models. The engine fuel economy data for vehicles sold historically were obtained from http://toyota.jp/service/, along with the price data and engine size data.
Canada	Official websites of car manufacturers in Canada for the existing models. For the missing/outdated models, we have used prices from the USA.	Official websites of car manufacturers in Canada for the existing models. For the missing/outdated models, we have used fuel economy from the USA.	Official websites of car manufacturers in Canada for the existing models. For the outdated/old models, fuel economy data were excluded from the calculation.
Australia	Official websites of car manufacturers in Australia for the existing car model prices. For old models, we have obtained prices data from http://www.redbook.com.au/	Official websites of car manufacturers in Australia for the existing car model engine sizes. For old models, engine sizes data can be obtained from http://www.redbook.com.au/, along with the price data and fuel economy data	official websites of car manufacturers in Japan for the existing models. The fuel economy data for vehicles sold historically were obtained from http://www.redbook.com.au/, along with the price data and engine size data.
New Zealand	We have used car prices in Australia for New Zealand	We have used engine sizes obtained for Australia in New Zealand.	We have used fuel economy obtained for Australia in New Zealand.
Russia	Official websites of car manufacturers in Russia for the existing models. The prices data for vehicles sold historically/outdated vehicles were obtained from http://auto.mail.ru/ and http://www.avtomarket.ru/	Official websites of car manufacturers in Russia for the existing models. The engine size data for vehicles sold historically were obtained from http://toyota.jp/service/dealer/spt/search-addr, along with the price data and the fuel economy.	Official websites of car manufacturers in Russia. For the outdated/old models, fuel economy data were excluded from the calculation.
China	Car prices data (both new vehicles and old models) were obtained from commercial dealer websites, such as China Auto Home (http://www.autohome.com.cn/) and Sohu Auto (http://auto.sohu.com/).	Engine sizes data were collected from http://www.autohome.com.cn/ and http://auto.sohu.com/, along with price and fuel economy data.	Fuel economy data were collected from http://www.autohome.com.cn/ and http://auto.sohu.com/, along with price and engine sizes data.

Table 5: Summary of data sources continued

Country	Car price	Engine size	fuel economy
India	Car prices data have been obtained from the official car manufacturers′ website for India. For old models, we have obtained prices data from http://www.carwale.com/new/ and http://www.zigwheels.com/newcars	Engine sizes data were obtained from the official car manufacturers′ website for India, alongside price data. Similarly, for old models, engine sizes data were obtained from http://www.carwale.com/new/ and http://www.zigwheels.com/newcars	If available, fuel economy data were obtained from the official manufacturers′ website for India. For some new models (where fuel economy data are not available from the manufacturers) and outdated models, fuel economy data were obtained from http://www.carwale.com/new/ and http://www.zigwheels.com/newcars
Mexico	Car prices data have been obtained from the official car manufacturers′ website for Indonesia. For old or unavailable models, we have not been able to obtain data so they are not considered in the calculation.	Car engine sizes data were obtained from the official car manufacturers′ website for Mexico. For old or unavailable models, we have used engine sizes obtained for other Latin America countries (e.g. Brazil) if available.	Fuel economy data were obtained from the official car manufacturers′ website for Mexico. For old or unavailable models, we have used fuel economy obtained for other North America countries (e.g. USA) if available.
Brazil	Car prices data have been obtained from the official car manufacturers′ website for Brazil. For old models, we have obtained prices data from http://www.icarros.com.br/catalogo/	Car engine sizes data were obtained from the official car manufacturers′ website for Brazil. For old models, we have obtained the engine sizes data from http://www.icarros.com.br/catalogo/, along with car prices data.	Car fuel economy data were obtained from the official car manufacturers′ website for Brazil. For old or unavailable models, we have used fuel economy obtained for other North America countries (e.g. USA) if available.
Argentina	Car prices data have been obtained from the official car manufacturers′ website for Argentina. For old models, we have obtained prices data from http://www.cars.com.ar/	Car engine sizes data were obtained from the official car manufacturers′ website for Argentina. For old models, we have obtained the engine sizes data from http://www.cars.com.ar/, along with car prices data.	Car fuel economy data were obtained from the official car manufacturers′ website for Brazil. For old or unavailable models, we have used fuel economy obtained for other North America countries (e.g. USA) if available.
South Korea	Car price data have been obtained from the official car manufacturers′ website for South Korea. For old or unavailable models, we have taken the prices from Japan.	Car engine size data were obtained from the official car manufacturers′ website for Indonesia. For old or unavailable models, we have taken engine data from Japan.	Car fuel economy data have been obtained from the official car manufacturers′ website for South Korea. For old or unavailable models, we have taken data from Japan.
Taiwan	Car prices data have been obtained from the official car manufacturers′ website for Taiwan. For old models, we have obtained prices data from https://tw.autos.yahoo.com/car-research	Fuel economy data were collected from https://tw.autos.yahoo.com/car-research, along with price and engine sizes data.	

Table 6: Summary of data sources continued

Country	Car price	Engine size	fuel economy
Indonesia	Car prices data have been obtained from the official car manufacturers′ website for Indonesia. For old or unavailable models, we have not been able to obtain data so they are not considered in the calculation	Car engine size data have been obtained from the official car manufacturers website for Indonesia. For old or unavailable models, we have not obtained data so they are not considered in the calculation.	Car fuel economy data were obtained from the official car manufacturers website for Indonesia. For old or unavailable models, we have not obtained data so they are not considered in the calculation.
Malaysia	Car prices data have been obtained from the official car manufacturers′ website for Malaysia and Singapore. For old models, we have obtained prices data from http://www.sgcarmart.com/newcars/	Car engine sizes data were obtained from the official car manufacturers′ website for Malaysia and Singapore. For old models, we have obtained the engine sizes data from http://www.sgcarmart.com/new_cars/	Car fuel economy data have been obtained from the official car manufacturers′ website for Malaysia and Singapore. For old or unavailable models, we have not obtained data so they are not considered in the calculation.
Saudi Arabia	Car prices data have been obtained from the official car manufacturers′ website for Saudi Arabia. For old models, we have obtained prices data from http://saudi.dubizzle.com/en/home/, http://www.carsemsar.com/en/saudi-arabia and http://www.drivearabia.com/	Car engine sizes data were obtained from the official car manufacturers′ website for Saudi Arabia. For old models, we have obtained the engine sizes data from http://www.drivearabia.com/ , along with car prices data.	Car fuel economy data were obtained from the official car manufacturers′ website for Malaysia and Singapore. For old or unavailable models, we have not obtained data so they are not considered in the calculation.
South Africa	Car prices data have been obtained from the official car manufacturers′ website for South Africa. For old models, we have obtained prices data from http://www.cars.co.za/.	Car engine sizes data were obtained from the official car manufacturers′ website for Saudi Arabia. For old models, we have obtained the engine sizes data from http://www.cars.co.za/. , along with car prices data.	Fuel economy data were obtained from the official manufacturers′ website for South Africa. For some new models (where fuel economy data is not available from the manufacturers) and outdated models, fuel economy data have been obtained from http://www.cars.co.za/.

Table 7: List of E3ME world regions

E3ME region	Countries	E3ME region	Countries	E3ME region	Countries
1-28: EU-28	EU-28	39: Russian Fed.	Russian Fed.	50: Indonesia	Indonesia
29: Switzerland	Switzerland	40: Rest Annex I	Belarus	51: ASEAN	Thailand, Cambodia, Lao, Malaysia, Myanmar, Philippines, Singapore, Vietnam
30: Iceland	Iceland	41: China	China	52: OPEC	Iran, Iraq, Kuwait, Qatar, UAE
31: Croatia	Croatia	42: India	India	53: Rest of World	All other countries
32: Turkey	Turkey	43: Mexico	Mexico	54: Ukraine*	Ukraine
33: Macedonia*	Macedonia	44: Brazil	Brazil	55: Saudi Arabia*	Saudi Arabia
34: USA	USA	45: Argentina*	Argentina	56: Nigeria*	Nigeria
35: Japan	Japan	46: Colombia	Colombia	57: South Africa*	South Africa
36: Canada	Canada	47: Rest of Latin America	Bolivia, Chile, Ecuador, Peru, Central America	58: Rest of Africa*	Rest of Africa
				59: Africa OPEC*	Algeria, Angola, Libya
37: Australia	Australia	48: South Korea	South Korea		
38: New Zealand	New Zealand	49: Taiwan*	Taiwan		

Notes: Note: stars denote countries not included in FTT:Transport, where fuel used is assumed proportional to the World average. In these countries, either some data is missing, or it has to do with that the E3ME classification recently changed from 53 to 59 countries and FTT:Transport has not yet been updated accordingly. Thus, the FTT:Transport classification is bound to change in the near future. Some regions, however, may never be modelled, due to lack of data.

Table 8: Parameterisation of the model outside the 18 nations where direct data were available.

Country	Proxy country
EU 28, Norway, Switzerland, Iceland, Croatia, Turkey and Macedonia	UK
Rest of Annex 1	We have taken Ukraine as the representative country in Rest of Annex 1.We have used car prices in Russia for Ukraine.
Colombia	We have used car prices collected for Brazil to represent prices in Colombia.
Rest of Latin America	We have taken Brazil as the representative country in Rest of Latin America.
ASEAN	We have taken Malaysia as the representative country in ASEAN.
OPEC	We have taken Saudi Arabia as the representative country in OPEC.
Rest of world	We have taken South Africa as the representative country in this category.

and

$$VS_j = \beta_{1,j} * RRPD_j + \beta_{2,j} * PV_j + \beta_{3,j} * ST_j + \epsilon_j \qquad (64)$$

where PM is the demand for transport in passenger kilometres (pkm), $RRPD$ is the real income, $PFRM$ is the price of middle distillates for road transport, VS is the number of new vehicle sales and PV is the real price of vehicles and ST is the number of vehicles on the road, all specified for each region j.

In the FTT:Transport integration to E3ME, $RRPD$ and $PFRM$ are modelled endogenously, while ST is obtained from VS by integrating with the survival function. Thus E3ME supplies endogenous values of PM and VS to FTT:Transport, which determines fleet compositions, and feeds PV and ST back to E3ME. FTT:Transport furthermore feeds back to E3ME its estimation of fuel use for road transport. It is to be noted that in this formulation of feedbacks, changes in the use of liquid fossil fuels from FTT:Transport has large impacts on economic activity in oil producing countries.

For the projections of future car sales, we have obtained the number of car fleets (numbers of vehicles registered) for the global 59 countries (1990-2012) from the Euromonitor website.

5.5. *Determining the non-pecuniary γ values in practice*

When $\gamma_i = 0$, we obtain a rate of diffusion that does not normally match historical diffusion (see Fig. 4 of the main paper, reproduced here in fig.17). One, and only one, set of γ_i leads to the diffusion of technology in the simulation to have the same rate as the historical rate at the starting point of the simulation. Since decisions are based on cost differences, the number of γ_i parameters equals that of technologies minus one (or one of the γ_i equals zero). As found by experience, the unique set of γ_i cannot be obtained by simple optimisation, as too many spurious local solutions arise. We thus designed a dedicated graphical user interface software that enables to robustly determine these parameters by hand. This is done for each technology in every region, thus a time-consuming procedure, but visual inspection ensures that the parameters are not spurious.[14] We find that γ_i values follow what is expected: luxury models have large negative values (large benefits). We typically keep the value for the small economic petrol vehicles near zero; however any arbitrary constant can be added to all γ_i without consequence to the results. This is a reflection that FTT represent flows of shares according to relative generalised cost differences between categories. Note that since generalised cost differences already exist in the baseline, *diffusion trends exist in the baseline*, a fact that is observed in the data, and the determination of the γ_i parameters is of primary importance. Although it does not provide significant information on the non-pecuniary benefits themselves, it is in this way a robust methodology.

Figure 17 shows an example of visual adjustment of the γ_i parameters for the UK, with the graphical user interface used. Note that it is critically important to obtain good values for technology categories with large share values. Meanwhile, it is sometimes *difficult* or *ambiguous* to determine γ_i values for some new technologies, when they have small and/or noisy historical data, and sometimes no data is available at all. However, the γ_i values are *strongly tied to vehicle class categories* (Econ, Mid, Lux), as we observe across countries, but less so to engine technologies types (Petrol, Diesel, EVs). However, they are not particularly tied to particular countries. For example, a luxury diesel vehicle is desirable in a roughly similar way to a luxury petrol vehicle, but not to an economic petrol vehicle. The relative difference in γ_i between technology categories is always much smaller than across engine size classes. It is thus feasible to use proxies for missing values, either across similar technology types (Petrol and Diesel) or across countries for the same technologies (e.g. EV Econ). Thus in instances where γ_i values could not be obtained using the visual interface, for instance if they have zero shares, they were inferred from other technology categories within the same engine size class. This is because it is not realistic to assume that these technologies would have zero shares forever, and indeed, we use in some cases kick-start policies for these categories to start diffusing (e.g. EVs in India).

Finally, it is to be noted that changing one γ_i value in a set for one region requires to re-determine all the others, as it changes the relative value of all technologies. Furthermore, if the definition of the LCOT is changed for any reason (e.g. adding a pecuniary parameter, or changing the discount rate), the empirical γ_i must all be re-determined since their meaning also changes. In this sense the γ_i contain everything of relevance that is not explicitly represented in the LCOT; the more parameters are included in the LCOT, the less are implicitly represented in the γ_i. It takes a few hours to determine all γ values.

[14]Determining the set takes approximately one hour.

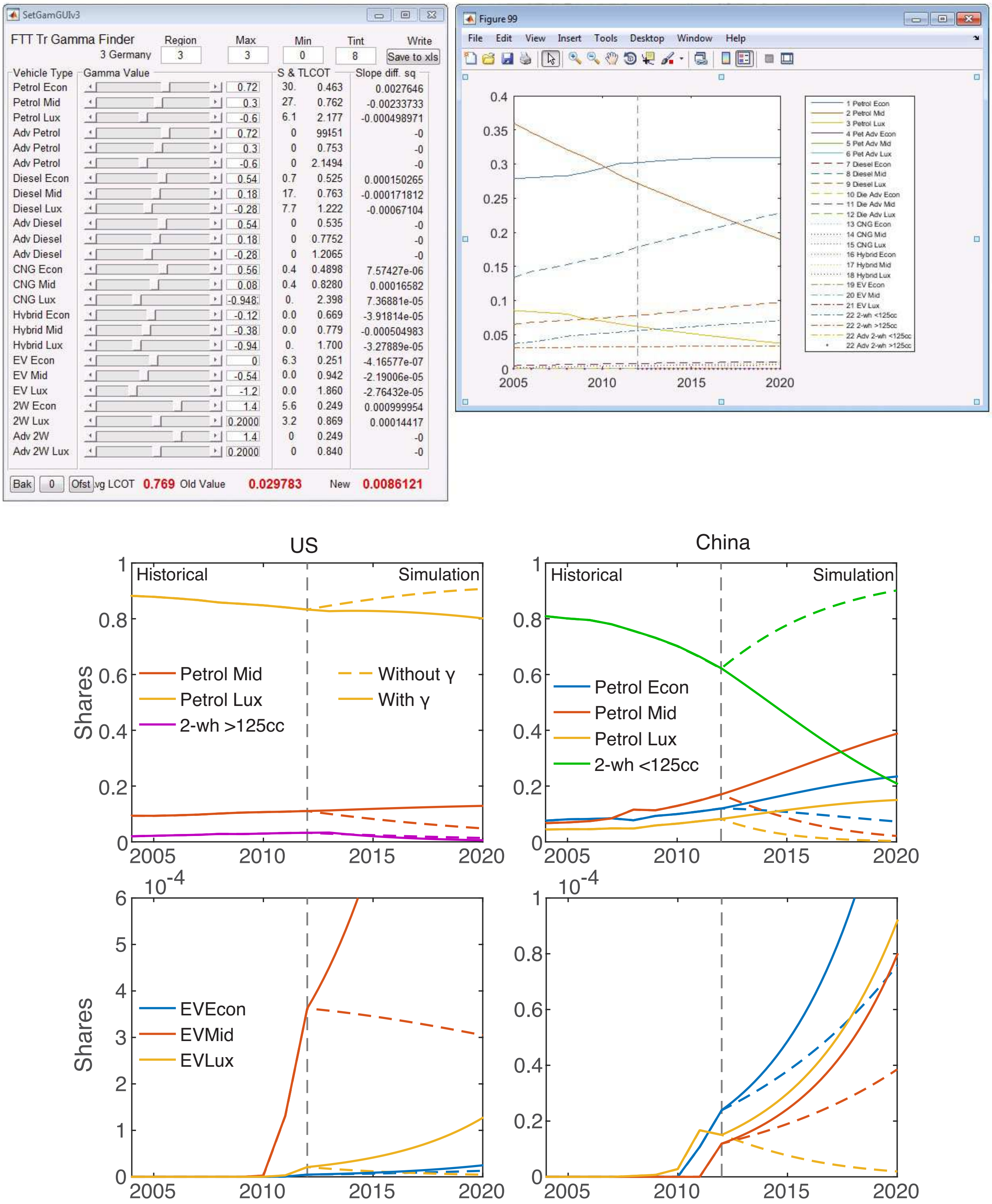

Figure 17: Graphical user interface used to determine γ parameters. Sliders or value inputs are used in order to change the diffusion trajectory of the model (to the right of the dashed line), until it is consistent with historical data (to the left of the dashed line). This is done for every technology in every country, meaning that $59 \times 25 = 1475$ parameters are adjusted. The fact that this is done by hand ensures that every single diffusion profile has been inspected. Technologies with lower shares are parameterised in the same way by zooming in.

6. Sensitivity analyses and model validity

In this section, we carry out a sensitivity analysis over most relevant technological parameters of FTT:Transport. We say *most* since we could, in principle, produce pages and pages of tables of numbers for additional different sensitivities, but we consider that this would increase substantially the amount of insight in comparison to what is given here. We chose the parameters that we expected would generate the most changes in emissions and technological shares. For example, we put emphasis on EVs since these have the most impact on transport emissions. However, the technological trajectory itself is important, and therefore we provide both changes on emissions and changes on shares of technologies. All sensitivities are carried out globally, meaning that the parameters are changed in the same way in every country. It is clear that in each country, these changes have a different impact (e.g. changing EV costs in countries with few EVs has low impact), however, we do not consider sufficiently instructive to generate this data for every country.

Results are given in table reftable:models. Numbers shown refer to percent changes in a scenario in which a parameter variation is imposed, against the corresponding scenario without the variation. Changes in shares are in 2050, while changes in emissions are cumulated to 2050. Variations are in percent of the cost if applied to cost values (e.g. 20%), or in percentage points if applied to a rate (e.g. learning and consumer discount rates). We find that results are not highly sensitive to any particular parameter.

The highest changes we see are the changes that arise for changes in the γ values. In particular, if we amplify the differences between the γ (first and second rows, $\gamma \pm 20\%$), we see re-allocations of shares between categories, especially across engine size classes, of at most 30%. If we change γ of vehicle classes, we also see substantial re-allocations of shares across engine size classes. Importantly, however, changing the γ of EVs does not produce large changes in the allocation of shares. This is because shares of EVs are low to start with, so changing their parameters has a comparatively low influence on the overall trajectory, until EVs take a substantial market share in around 2050. It is to be noted that changing values of γ implies diffusion trajectories that are inconsistent with historical data for all vehicle types (i.e. broken at the start year of the simulation), and therefore can in themselves be quite unrealistic. For example, changing the γ of mid-range petrol vehicles implies changes in diffusion trajectory of all other vehicle types, larger for larger share categories. γ values for low-share low-carbon vehicles are more uncertain than those for large-share categories, and correspondingly, have less impact on the overall trajectory (i.e. larger changes are needed).

We provide in table reftable:models combinations of uncertainties across all variations carried out. We do not know what the uncertainty over these parameters is in reality. However, experience with data tells us that these variations are reasonable. The interpretation of this is therefore that if the real uncertainty over these parameters matched what has been used here, and that all of these were introduced simultaneously (e.g. in a Monte-Carlo analysis), we would obtain the variations given in the rows labelled 'Root sum square', where the root of the sum of the squares was calculated. In the bottom row of the table, combined uncertainty is given for a scenario comparison between the baseline and the 2°C scenario. We find that at most 50% uncertainty is generated (in this case, for EV shares in 2050). This may seem relatively large; however this corresponds only to 30% change in transport emissions, and thus does not have a major impact on any climate scenario studied.

6.1. Sensitivity of technological parameters

In this section, we analyse responses of the model to changes in its key parameters. Key parameters of FTT:Transport include

1. All cost parameters explicitly specified (vehicle prices, fuel costs, consumer discount rates, learning rates etc),
2. Technical parameters (fuel efficiency, emissions factors, lifetimes),
3. Non-pecuniary costs not explicitly specified (i.e. the γ_i values).

Some parameters are equivalent or related, for example emissions factors and the fuel efficiency. Some parameters are derived from data (e.g. γ_i values, prices, emissions factors), while others are directly taken from the literature (e.g. learning rates, discount rates). Therefore, it is possible that some choice of parameters are non-sensical or lead to violating the assumptions of the model. This happens for instance if we vary vehicle prices without re-estimating γ_i values, which makes diffusion trajectories in the simulation inconsistent with trajectories observed in historical data. Such scenarios are by definition not self-consistent and thus not realistic, but useful for analysing the properties of the model.

With these issues in mind, it is important to analyse model responses to variations in key parameters, in order to ensure that the model is not 'highly sensitive' to very specific values for any particular parameter. As a benchmark, if the outcome variation is less than the input variation, we assume here that the model is not 'highly sensitive' to the particular values chosen.

We varied 28 parameters or sets of parameters, by quantities that we consider either representative of uncertainty, or sensible values for observing model responses, shown in table 9. We did this in both our 'current trajectory' and our 2°C scenarios. The reason for doing so is that policies constrain model evolution in particular directions, and thus one should not expect to find the same response (decarbonisation policies constrain outcomes more). Variations were carried out in all countries simultaneously, for simplicity, as we expect similar results to be observed at the regional level.

However, we did not vary the list of possible technologies, as is often done in cost-optimisation models. The reason for this lies with our modelling philosophy in order not to violate our assumptions. The reason for which we would not remove technologies is that FTT:Transport only models the diffusion of technologies already sold in the market, as evidenced by our historical database. It would not be consistent with reality to remove any of these. The reason for which we do not add technologies (e.g. 'disruptive technologies') is due to our modelling time-frame: we already know from diffusion data, turnover rates and literature that technologies with vanishingly small market shares in the present day can only marginally change the market by 2050. Thus, sensitivity analyses that would consist of only adding a radically new technology at near-zero shares will by FTT construction make a difference of a marginal order to model outcomes. This is true unless specific action is taken, for example, with a relatively large government-led purchasing programme. Linking an ambitious programme of that kind with a new technology could indeed change outcomes to an appreciable degree; however we consider this within the realm of technology push policy, not a sensitivity analysis.

The parameters varied here are as follows:

1. *All γ_i values simultaneously*; (for all vehicle types)
2. *Learning rates of all vehicle types*;
   Note that learning cost reductions for conventional vehicles are marginal even when changing rates, due to their existing market domination.
3. *Consumer discount rates*; (equal for all vehicle types)
4. *The price of electric vehicles*;
   Note that changing vehicle prices without changes of γ_i breaks FTT assumptions and leads to broken diffusion trends at the start of the simulation. Changes can made within fitting accuracy are acceptable.
5. *The fuel efficiency of new liquid fuel engines*; (ADV category).
   This affects both emissions and the attractiveness of vehicles.
6. *The rate of vehicle purchases (turnover rates)*;
   Note that, as noted in section 3.3.4, these are not the same as the rates of scrappage, and in fact are not directly related. Turnover rates here mean the rate of acquisition of new vehicles, i.e. the rate of decision-making. Note further that changing turnover rates without changing γ_i leads to highly inconsistent model definitions, and

	Parameter	% change in CO_2 and technology shares over the same scenario without changes								
		CO_2	Econ	Mid	Lux	Hybrid	CNG	EV	ADV	FF
**2°C Scenario**	1- All γ +20%	2.31	-11.58	6.86	12.04	17.47	-1.75	-2.35	1.07	9.30
	2- All γ -20%	-0.05	7.47	-6.14	-10.30	-16.09	-14.44	12.12	-8.00	-16.16
	3- Learning rates +5%	3.38	-2.97	0.52	7.62	-1.06	0.49	1.37	-0.02	-6.71
	4- Learning rates -5%	-13.05	-8.22	3.09	-5.80	2.53	-20.24	4.53	-7.98	7.14
	5- Discount rates +10%	-16.14	-13.80	10.09	-5.60	14.18	-28.07	12.89	-12.12	-3.75
	6- Discount rates -10%	14.30	8.35	-7.17	6.76	-11.26	20.55	-11.79	9.89	5.34
	7- EV prices +10%	1.67	-0.06	0.84	-1.93	1.76	0.88	-2.46	1.13	0.09
	8- EV prices -10%	-1.38	0.37	-0.95	1.78	-1.44	-0.84	2.85	-1.24	-0.14
	9- Fuel efficiency +20%	7.90	-0.09	-1.12	2.45	-5.78	-0.14	-0.59	0.41	-4.03
	10- Fuel efficiency -20%	-6.99	0.33	1.46	-3.54	6.82	0.14	0.81	-0.52	5.43
	11- Turnover Rate +50%	55.82	-15.83	-3.02	9.65	-27.07	24.00	-79.83	28.33	1304.87
	12- Turnover Rate +25%	37.00	-4.11	-4.88	0.86	-10.43	30.00	-57.26	23.68	393.97
	13- EV γ +10%	-0.65	-1.19	-0.39	3.31	-0.55	-0.62	0.83	-0.42	-0.01
	14- EV γ -10%	0.16	1.27	0.42	-3.52	0.83	0.70	-1.04	0.53	0.01
	15- Hybrid γ +10%	1.66	-0.88	3.13	-3.26	11.90	-0.17	-0.80	0.57	-0.06
	16- Hybrid γ -10%	-0.98	0.82	-2.92	3.12	-10.43	0.12	0.57	-0.41	0.06
	17- CNG γ +10%	0.34	-3.25	2.52	1.04	0.25	-0.44	0.09	-0.49	0.14
	18- CNG γ -10%	0.38	3.13	-3.04	0.10	-0.22	1.17	-0.14	0.50	-0.12
	19- ADV γ +10%	0.13	-0.88	-2.32	6.33	-1.57	-0.30	-0.25	0.22	-0.51
	20- ADV γ -10%	0.40	1.32	1.20	-5.14	1.26	0.44	0.15	-0.13	0.46
	21- 2W γ +10%	0.75	0.51	-0.02	-0.52	-0.16	2.26	-2.03	1.22	5.58
	22- 2W γ -10%	-2.65	-2.14	0.48	1.24	0.18	-6.82	4.76	-3.26	-7.65
	23- Econ γ +10%	-1.15	2.59	-3.49	-0.79	1.26	-2.30	0.78	-0.87	-0.77
	24- Econ γ -10%	1.26	-4.84	6.29	0.48	-1.10	2.17	-0.80	0.85	0.89
	25- Mid γ +10%	-0.54	-9.31	14.89	-6.32	9.05	0.37	-0.52	0.41	0.03
	26- Mid γ -10%	1.32	6.56	-11.68	6.57	-7.85	-0.17	0.30	-0.26	-0.03
	27- Lux γ +10%	3.38	-2.09	-5.83	15.41	-0.30	0.19	-0.22	0.27	0.71
	28- Lux γ -10%	-1.38	1.95	4.13	-11.89	0.69	0.47	-0.09	-0.05	-0.58
	**Root sum sq. 1-10**	27.71	22.95	15.90	21.10	31.37	42.81	22.25	19.41	23.07
	**Root sum sq. γ**	5.52	14.15	22.41	24.00	20.06	8.05	5.59	3.93	9.61

Table continued...

	Parameter	% change in CO_2 and technology shares over the same scenario without changes								
		CO_2	Econ	Mid	Lux	Hybrid	CNG	EV	ADV	FF
**Current Trajectory Scenario**	1- All γ +20%	4.34	-9.96	4.74	33.76	5.44	-1.07	-5.36	1.40	-0.19
	2- All γ -20%	-4.91	11.48	-14.12	-24.74	-4.77	-3.00	14.59	-2.78	-1.19
	3- Learning rates +5%	-1.86	-4.30	7.62	5.36	7.53	-1.41	2.16	11.45	-27.28
	4- Learning rates -5%	6.70	3.64	-6.44	-3.88	-6.78	-0.72	-9.23	-7.85	22.29
	5- Discount rates +10%	-2.19	2.78	-4.77	-5.89	2.63	-0.86	6.93	0.25	-4.15
	6- Discount rates -10%	2.46	-4.00	7.16	8.01	-2.28	1.02	-8.98	0.10	4.42
	7- EV prices +10%	3.13	0.84	0.38	-3.18	3.94	1.09	-9.67	1.66	0.99
	8- EV prices -10%	-6.66	-9.01	14.45	6.68	-9.69	-1.87	-46.47	-1.53	26.37
	9- Fuel efficiency +20%	1.86	0.07	0.11	-0.32	-0.36	-0.02	-0.03	2.33	-5.31
	10- Fuel efficiency -20%	-1.49	-0.04	-0.03	0.13	0.29	0.02	0.02	-1.90	4.33
	11- Turnover Rate +50%	30.18	-21.85	26.73	25.20	-45.51	-17.40	-88.55	-15.83	79.62
	12- Turnover Rate +25%	16.39	-9.15	14.83	5.85	-21.99	2.13	-70.07	-0.14	34.80
	13- EV γ +10%	-1.36	-0.84	-0.39	3.21	-1.52	-0.48	4.08	-0.69	-0.45
	14- EV γ -10%	1.62	0.91	0.44	-3.49	1.95	0.51	-4.74	0.80	0.51
	15- Hybrid γ +10%	0.16	-0.49	1.87	-0.90	8.62	-0.25	-1.30	1.34	-2.41
	16- Hybrid γ -10%	-0.03	0.29	-1.26	0.70	-6.82	0.13	1.17	-1.07	1.88
	17- CNG γ +10%	-0.03	-1.64	4.34	-0.81	-0.19	0.95	-1.59	0.52	-0.40
	18- CNG γ -10%	0.12	1.42	-3.98	0.99	0.22	-0.98	1.42	-0.52	0.48
	19- ADV γ +10%	0.49	-0.58	-1.96	5.25	-1.08	-0.33	-0.37	2.47	-5.44
	20- ADV γ -10%	-0.21	0.68	-0.02	-2.52	0.43	0.11	0.19	-1.44	3.18
	21- 2W γ +10%	1.63	-0.86	3.52	2.51	-1.19	-0.31	-3.30	0.22	1.32
	22- 2W γ -10%	-2.52	0.75	-3.27	-2.21	0.80	-0.36	6.02	-0.69	-1.58
	23- Econ γ +10%	0.93	2.94	-5.84	-1.29	0.55	0.29	-1.75	0.25	0.29
	24- Econ γ -10%	-0.85	-3.18	6.83	0.68	-0.47	-0.40	1.42	-0.22	-0.21
	25- Mid γ +10%	-0.74	-6.15	19.44	-5.70	4.63	0.25	-0.76	0.82	-1.44
	26- Mid γ -10%	1.09	4.77	-15.75	5.41	-3.28	-0.23	0.24	-0.39	0.73
	27- Lux γ +10%	2.02	-2.70	-9.71	23.86	-1.46	-1.26	1.87	-0.76	0.88
	28- Lux γ -10%	-0.93	2.38	6.57	-17.76	1.42	0.92	-2.51	0.72	-0.46
	**Root sum sq. 1-10**	12.73	19.19	24.58	44.15	16.64	4.37	52.08	14.72	44.97
	**Root sum sq. γ**	4.69	10.05	30.19	31.92	12.94	2.36	10.47	3.91	7.61
	**Combined error**	31.35	34.58	47.65	63.16	42.79	43.84	57.87	24.98	52.01

Table 9: Sensitivity analysis on key technological parameters. Each number refers to a percentage change in CO_2 emissions or technological shares (share of the total fleet), in a scenario with parameter change, with respect to the same scenario without variation. Variations used are what we consider realistic uncertainty values. Changes in rates (e.g. learning rates) are percentage point changes (e.g. learning rate of 15% changed to 20%). Outcome changes on emissions are cumulated to 2050, while for shares the values are in 2050. Turnover rates represent the rate of replacement of the fleet. Note that when changing the turnover rates, the γ values lose meaning and the projections *do not match* trends observed in historical data. In other words, slower turnover rates cannot reproduce observed rates of technological change, and are included for reference, but are not realistic. The root of the sum of the squares of the variations are given for the technological parameters (rows 1-10) and all the individual γ values (rows 13-28), excluding turnover rates. Root sum square values can be interpreted as combined uncertainty if the variations were normally distributed and known. Here, there are not precisely known or known to follow a particular distribution, and thus these values do not correspond to actual uncertainty, but instead, give an indication of model response to variations. The variations in rows 13-20 are shown graphically in fig. 18.

moreover, often leads to the inability to explain historical data in the case of fast diffusing technologies (such as electric vehicles).

7. γ_i *values for EVs, Hybrids, CNGs, ADV and 2-Wheelers;*;
   ADV stands for higher efficiency new internal combustion engines, while 2W are motorcycles.
8. γ_i *values for vehicle class categories*;
   Note that these are changed for all vehicle types, for one engine size class at a time.

We conclude with this analysis the following broad findings. (1) Learning and discount rates and vehicle prices have a relatively large impact on results, however, only of a similar order to the variations imposed; (2) Changing turnover rates has a relatively large impact on results (changes in outcomes much larger than changes in the parameters). (3) changing individual γ_i values has, in general, little impact on overall results (much smaller than the variations themselves), but leads to some re-allocation of shares across alternate vehicle types or class;

(1) This result is self-explanatory. Outcomes will depend on the particular form chosen for calculating the generalised cost to the consumer. This includes the choice of form for discounting future costs. Indeed, no-one can truly know what thought process takes places in the minds consumers when choosing vehicles, and that furthermore, one cannot ascertain the degree of diversity of explicit or intuitive methods used by consumers when deciding (whether all consumers even use similar decision methods or criteria, see e.g. Knobloch and Mercure (2016)). Thus, decision-making criteria are not clear; however, it is important to remember that in the FTT formulation, all cost values (excepting γ_i) are distributed, and that these distributions are combined. This allows for some degree of flexibility of interpretation, as different formulations for decision-making will not lead to radically different outcomes if differences remain within variations already explicitly specified. For example, if consumer discount rates are in reality lower than explicitly specified, or of consumers do not in fact discount following the standard method, if the changes remain within the range specified in vehicle prices data (mean and STD values), little difference will be observed in model outcomes.

(2) Turnover rates in FTT models are chosen in a way that (i) enables to fit the historical data, and (ii) is consistent with typical financial constraints. The reasoning behind this is that once costs are sunk, consumers are free to take new financial contracts. Here, it means that once a consumer has completed payments for a vehicle purchase (which typically takes 3-5 years), he is free, if he wishes to, to sell the vehicle and acquire a new one. However, it doesn't imply that he does so, and therefore, turnover rates in fact are upper limits to the rate of vehicle acquisition. In this, we assume that new vehicle markets are independent from second-hand vehicle markets, but that second-hand vehicle markets are 'slave' to new vehicle markets (see section 3.3.4). Changing turnover rates effectively implies changing the duration for which new vehicle consumers are financially constrained before they can replace their vehicle. This is not directly related to the lifetime of vehicles (or scrappage rates, which depend on a combination of failure rates and prices in the second-hand market). It also implies a different definition to γ_i values, which are contingent to a turnover rate. Changing the turnover rates changes the pace at which technological change takes place; such changes are partly compensated by corresponding changes in γ_i.

Here, changes of turnover rates are carried out without changes of γ_i. This implies highly inconsistent diffusion trajectories between projections and historical data. However, these changes also imply large changes in model outcomes. We found that slower rates makes fitting γ_i values often impossible in the cases of new technologies, in particular electric vehicles, as observed in historical data. It is clear that longer time-series would enable to better constrain turnover rates empirically. We therefore urge caution when assessing the impact of changing turnover rates in FTT:Transport. Here, the changes correspond to changing financial schedules length from 4 to 6 and 8 years.

(3) Changing one value of γ implies changing the attractiveness of one vehicle type, to the benefit or expense of all others. This leads, by construction, to violating the premise that diffusion trajectories are inferred from historical data. However, fitting γ_i values is accurate only to a certain extent, which we estimate at between 5% (established technologies) to 20% (technologies with short time-series). As noted in section 5.5, estimating can be reliably be made with time-series as short as 5 years. Here, we vary the γ_i by 10% for each technology type. We find that outcomes vary by between 0 and 10%. As intuitively expected, changes in the γ for one technology mostly affects its own pace of diffusion. The relatively low impact of varying these parameters is explained by the fact that these are done for individual technologies one at a time, which has relatively low impact overall in a multi-technology system: each alternative gains or loses a relatively small amount of market share. Changing several or all γ_i simultaneously has a higher impact, however. Note that additively changing all γ_i by a constant has no impact by construction, but

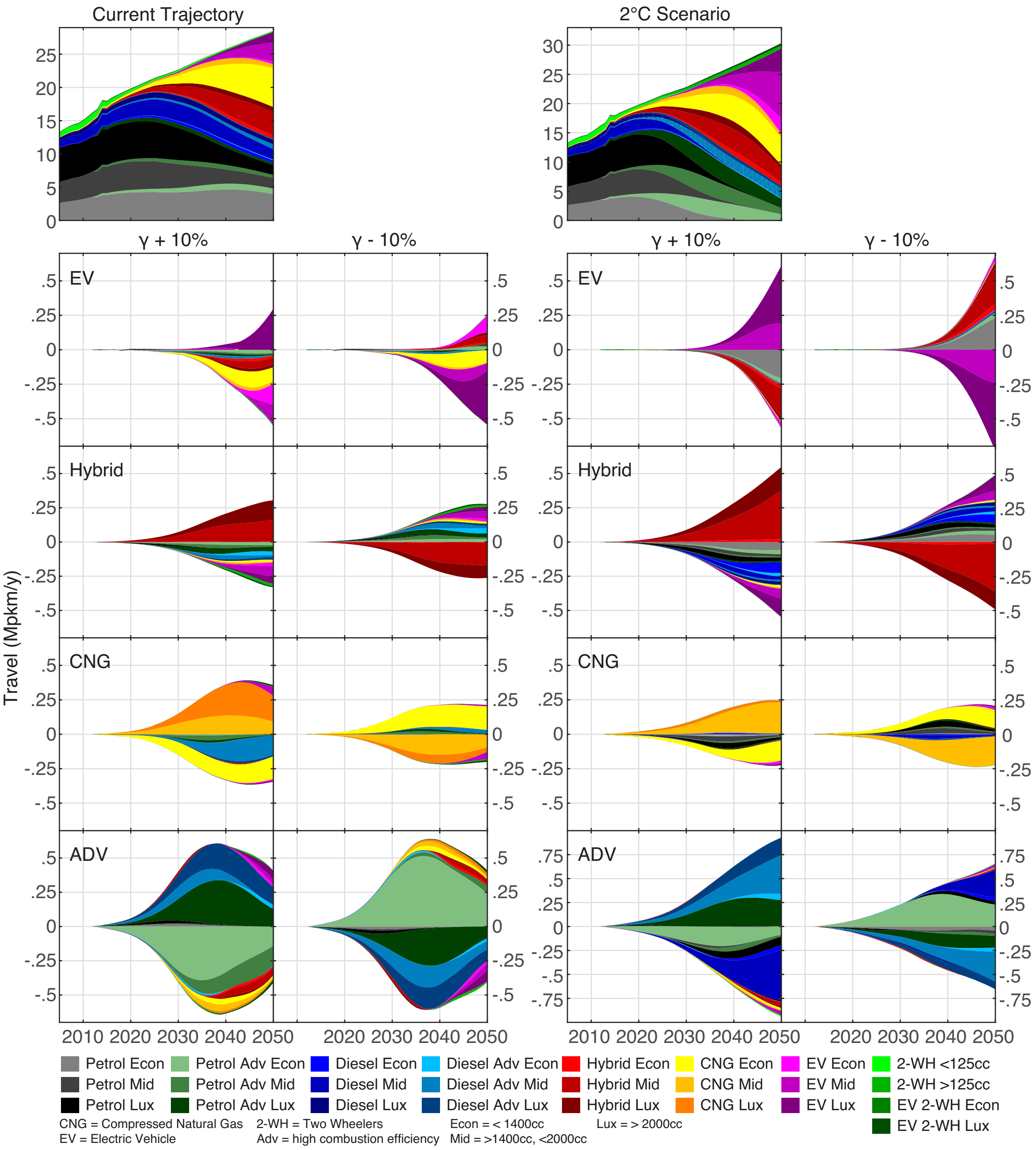

Figure 18: Sensitivity analyses of rows 13-20 in table 9, in which γ values are changed by ±10% individually, for electric (EV), hybrid, compressed natural gas (CNG) and new higher efficiency internal combustion engine (ADV) vehicles. Changes are expressed in terms of travel demand supplied by different vehicle types. To the left are variations applied to the 'current trajectory' scenario, while to the right are variations applied to a scenario with policies for achieving emissions consistent with a 2°C scenario. The actual scenarios without variations are shown in the top row, while the changes resulting from changing γ values are given below.

multiplying them by a factor does, since it creates relative changes.

Figure 18 demonstrates this in more detail, where changes are shown over modelling time-span. It can be observed that variations increase exponentially towards the end, starting from zero uncertainty at the starting year, by construction. This is a reflection of ‘diverging pathways’, which stems from the fact that FTT is a non-linear dynamical system (a complex system), akin to, for example, climate simulations (see Mercure et al., 2016b, for a discussion). Figure 19 shows differences observed between policy scenarios.

We conclude by observing the relative magnitude of combined errors, as given in table 9. If the variations introduced corresponded to normally distributed measured standard errors, then they should be combined using the root of the sum of their squares, given in the bottom rows. Comparing scenarios requires a further combinations (bottom row). Here, errors are not measured, and instead, we use estimated variations that we consider reasonable. Thus, the combined error is not to be interpreted as uncertainty; however it does provide a guide to assessing model behaviour. We observe combined variation of up to 30-40% in individual scenarios when all variations are taken into account simultaneously (when excluding changes to the turnover rate, for the reasons given above). This shows that even for extensive parameter changes, outcome scenarios are still fairly closely related to each other. This shows that the model is not dependent on any particular parameter. When comparing pairs of scenarios, up to 60% of variations are observed. This is an indication of the uncertainty as faced by, for example, policy-makers, knowing that the current trajectory as well as the policy objectives are subject to fundamental uncertainty.

6.2. *Comparison to other modelling approaches*

Behavioural aspects and heterogeneity significantly impact the effectiveness of market-based policies (Knobloch and Mercure, 2016). However, with the scale and time frames of IAMs, many IAMs have a simplified representation of consumer choice (Pettifor et al., 2017b). Global Integrated Assessment Models (IAMs) have been criticised for lacking behavioural realism (McCollum et al., 2016b). Consumer choices and behaviour are not sufficiently taken into account in most global IAMs, despite the fact that there is extensive evidence that consumer preference (e.g. non financial incentives) and heterogeneity (e.g. income and driving pattern) are the key drivers in the diffusion of vehicles (McCollum et al., 2016b). Since modelling consumer heterogeneity is crucial in determining the rate of diffusion for technologies, the general lack of focus of consumer heterogeneity limited the general usefulness of IAMs advising policies (Pettifor et al., 2017b).

In response to the general lack of consumer heterogeneity in the existing IAMs, several IAMs and studies have included different level of consumer diversity in their modelling approach (Karkatsoulis et al., 2016, McCollum et al., 2016b, Mittal et al., 2016, Pettifor et al., 2017b). (Pettifor et al., 2017b) used IMAGE and MESSAGE model to analyse the interactions between consumer preference and social influence in the transport sector. McCollum et al. (2016b) examines the impact of introducing consumer heterogeneity and choices into the MESSAGE-transport model. Karkatsoulis et al. (2016) includes values that represent preferences in vehicle types within a GCE framework (GEM-E3T). Mittal et al. (2016) uses AIM/transport model (with a special focus on Asia Pacific) to assess the impact of various factors such as travel time, energy efficiency improvement, load factor mode preference along with environmental awareness factors on transport demand, energy and emissions.

The existing studies improve the representation of consumer behaviour in global IAMs by considering some degree of heterogeneity and non-pecuniary factors that influence the consumer decisions. However, there is a main differences in modelling approach between FTT model and optimisation models used by the above studies. Firstly, the (McCollum et al., 2016b) introduced consumer heterogeneity into IAMs by separating adopters into groups, settlement pattern and vehicle usage intensity. This introduces consumer heterogeneity into the IAM MESSAGE with 27 different consumer groups. In the FTT model, within each technology, instead of having a mean representative agent (such as in MESSAGE), consumer diversity is represented by a distribution of vehicle prices (see for example figure 13), which we assumed to reflect the distributed willingness to pay for different car models by consumers. Hence, the heterogeneity is represented by the the distribution of car prices, not a representative agent. While both the (McCollum et al., 2016b) and our approach improve heterogeneity in representing consumers in the IAM, there are two main differences to results regarding available of technologies and the rate of technological diffusion. To illustrate, in the FTT model, with its historical market share database for each technology, reflects existing trends in different group of consumers and the availability and popularity of particular technologies in a particular country. With a different approach to heterogeneity, the rate of technological diffusion is expected to the different, and generate different results as to the rate of technology diffusion in each country.

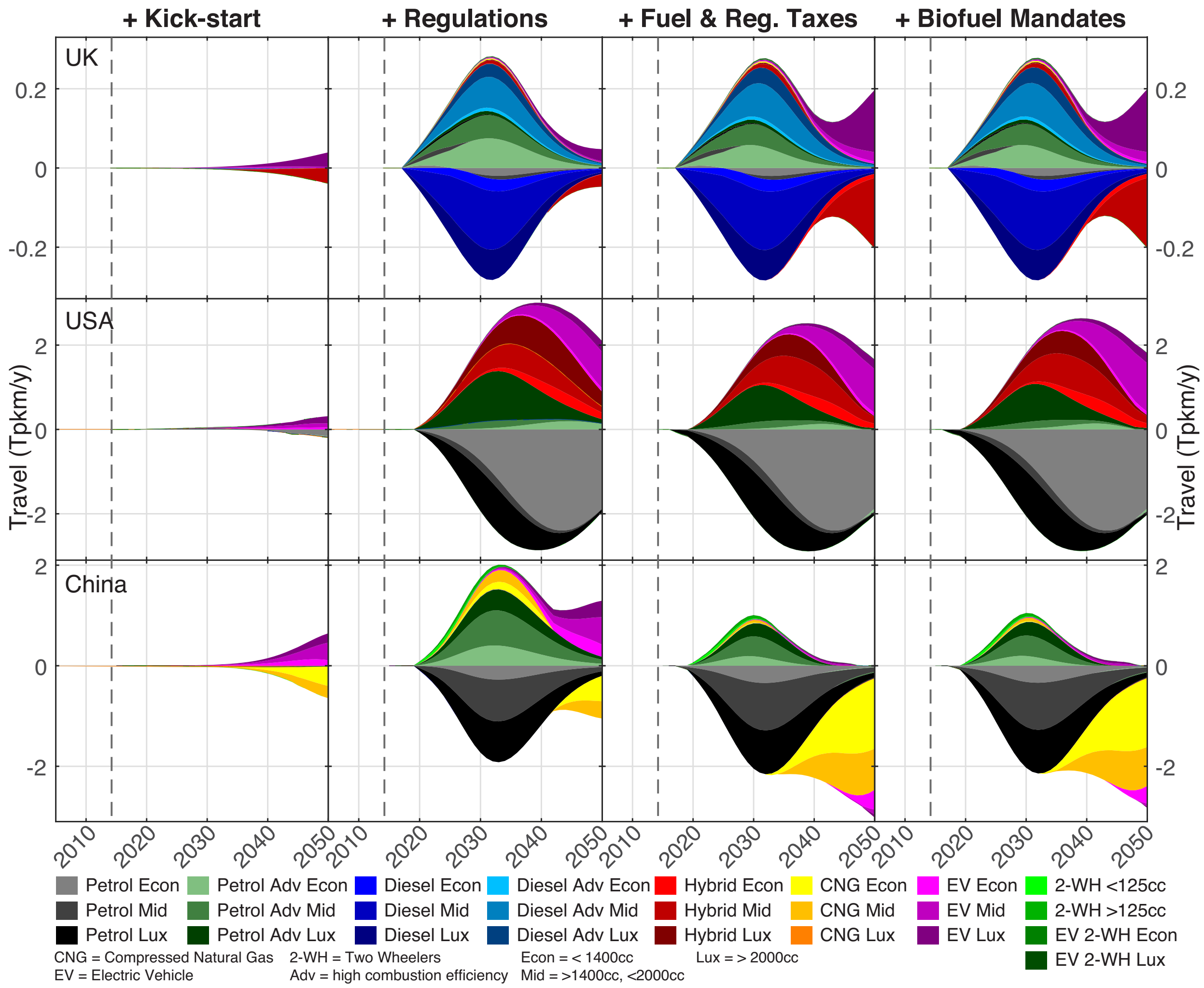

Figure 19: Figure showing the differences from the between the panels of figure 2 of the main text. Differences are taken from the baseline.

Indeed, what is observed is that technological change takes place faster in FTT than in the new version of MESSAGE (as seen in Pettifor et al., 2017b). In particular, EVs emerge in FTT with a sizeable market share in 2050, which happens in MESSAGE only nearer to 2100. We also observe that the diffusion of higher efficiency vehicles result in oil demand peaking before 2050, something not observed in MESSAGE. In FTT, the interpretation of this discrepancy is that the FTT diffusion trends are observed in historical data in recent years, and are projected, using the γ_i parameter described in section 5.5. This empirical observation helps ground the model into reality. We therefore argue that, while our method features inherent uncertainty that grows with simulation time span, improving optimisation models by adding ever more detailed cost data remains insufficient to explain current technology diffusion trends. Using a model grounded in empirical observation is likely to show results closer to what may unfold in the near future.

6.3. *Validity range of the model*

In the tradition of time series econometrics, the usual rule of thumb is that a reasonable validity range in time for an econometric model is to project for as many years forwards as one has years of historical data. This may be correct for linear econometrics models. But it is not for non-linear dynamical models such as FTT. We re-state and discuss the fundamental assumptions of the model:

1. The diffusion trend of technologies in the model should not be broken at the start of the simulation solely because it is the start of the simulation – real-world fleet numbers do not change so suddenly since vehicles remain used for 11-12 years on average, which generates substantial inertia. Therefore, we assume, the diffusion process (the number of vehicles and its first derivative) should be continuous across the transition between history and simulation, and this continuity can readily be observed in the data. This requires determining gamma values that ensure this is the case. This is the sole role of the gamma values.
2. The meaning of the gamma values can be interpreted in terms of the non-pecuniary value that agents ascribe to particular types of vehicles. In a pure cost-optimisation without non-pecuniary values, only the very lowest cost vehicles would diffuse, but this is of course not observed in reality (e.g. in our diffusion data). Gamma values are mostly needed to address price differences between our vehicle classes (e.g. Economic ~\$15k, Mid-range ~\$35k, Luxury ~\$50k), expressing the value that agents ascribe to them. For the model not to converge to lowest-cost vehicles only, one expects gamma values to be of a similar order of value as prices if all vehicle classes are seen to diffuse in the data consistently with recent history. This simply reflects the fact that the non-pecuniary value ascribed by agents to e.g. luxury cars is higher than that ascribed to e.g. economic cars. Similarly, if low-carbon vehicles are observed to diffuse in the historical period, we assume that agents ascribe to them a certain non-pecuniary value consistent with that diffusion. Being constants, gamma values also do not dominate or determine the calculation; it is changes in costs that determine outcomes. It is therefore not sensible to radically change gamma values (e.g. set them to zero) in the hope of carrying out a meaningful sensitivity test, as it is clear that the model would then generate nonsense, not being true to its own definition (i.e. pure pecuniary cost-optimisation, which is not observed in reality).
   The gamma values implicitly include the effect of current policies (e.g. taxes and rebates) applied to each vehicle type. It is possible that the policy may have changed during the historical period, in which case the gamma value determination will generate an average policy value over the historical period. However, we do not observe substantial sudden changes in vehicle numbers in our historical data (except for cases in which vehicle type definitions may have changed, or if radical policies were adopted shortly before the model start date). Therefore we do not consider correct to think that the determination of gamma values is made substantially fragile by changes in the policy regime that have taken place over our historical dataset. Furthermore, policies defined in the model are additional to existing policies implicitly included in the gamma value, not absolute.
   Policies may also have changed after the start of the simulation, in which case they would need explicit representation. We acknowledge that this could influence the outcomes of our model. However, as is the case for any model, addressing this requires to survey all policy changes in every world region of the model in detail, a substantially large task which is not the subject of this work. Including all existing policies in model baselines is a large task we believe very few modellers have carried out (with the notable exception of the IEA). We are making progress towards this for some important regions and hope to publish this separately.
3. The model by construction features substantial inertia, consistent with a substantial empirical literature, which means that radical changes cannot happen even for relatively large sudden changes in the data (e.g. costs or policies). Assuming that sensible gamma values are used that ensure that the diffusion trend is not broken due to the start of the simulation (as we cannot allow that the start year of the simulation should itself generate a discontinuity in sales in that year), inertia in the model ensures that the observed diffusion trajectory is consistently followed in early simulation years (i.e. the first decade), and departs from that in subsequent years due to the policy context.
   The sensitivity analysis provided in the first review round shows a relatively low influence of changes in gamma values on diffusion trajectories. This applies in particular for the first simulated decade, in which the model inertia rules out radical changes in the diffusion trajectory. This so-called 'strong autocorrelation in time' (an autocorrelation time span of order 10 years) in the model implies that the model can be used to project longer time spans than what is suggested by the standard rule of thumb for linear econometric models (~10 years), perhaps three times longer than the autocorrelation time, with uncertainty increasing over time (as in climate models). We do not, however, model beyond 2050, since clearly by then new innovations not foreseen by us nor specified in the model may start diffusing, or the gamma values will have changed, and thus the uncertainty is too large. Due to our model structure, changes in gamma values that would lead to substantially different projections have to be very large (changes > 20%, e.g. setting them to zero), and would also lead to highly broken diffusion trends at the start of the simulation, something that violates our assumption (1) and substantial

amounts of empirical work. In simpler words, the autocorrelation in time of around 10 years is due the fact that vehicles survive for around 10 years, which allows us to model longer time spans. This can be observed in Fig. 2 of the main text.

We finally note that the model equations provide a structure that needs to be calibrated (as any model normally does), but that is driven by internal dynamics (logistic diffusion) as much as by data and exogenous policy assumptions. The gamma value plays the role of a calibration parameter, similar to standard calibration procedures in other models, in order to match real world quantities. Meanwhile, policy assumptions are what steers model direction. We conclude by stating that the model validity range is of around 30 beyond the start date of the simulation, and therefore modelling to 2050 roughly matches the validity range, even if historical data used to calibrate the model is much shorter. The uncertainty range increases with the length of the projection; and the sensitivity analysis given above indicates what that range is. Meanwhile, uncertainty decreases when more recent historical data is added.

A. The addition of probabilistically distributed values

A.1. The addition is a convolution

In this project comes periodically the problem of adding values which have either probability distributions or that are really distributed in reality. The sum of such distributed quantities must also be distributed, and we know intuitively that the resulting distribution is a function of the original distributions, and that its *width* should be roughly the sum of the *widths* of the initial distributions. This is not far from the truth, but it can be derived formally.

Imagine that we have two quantities with the same units, A and B, distributed on the x axis following the probability distributions $p_A(x)$ and $p_B(x)$ (e.g. on a cost axis, A and B could be prices of particular items with a stochastic probability or a real distribution corresponding to real occurrences of these prices). If the values $\overline{A}$ and $\overline{B}$ are the *mean* values, and if the probability distributions are well defined, we expect the median of the sum to be $\overline{C} = \overline{A} + \overline{B}$, corresponding the the sum without distributions or uncertainty. But what about the distribution of the sum itself? And what about the width of the distribution of the sum? We can derive it here. Fig. A.20 shows this schematically.

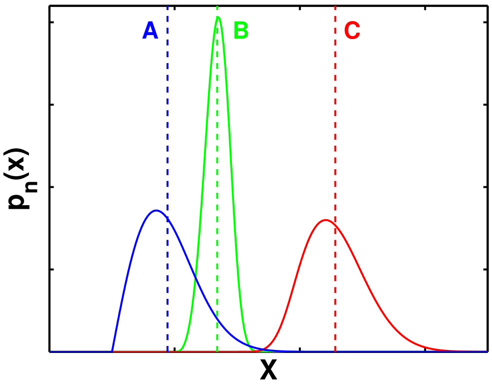

Figure A.20: The addition of probabilistically distributed quantities. The red distribution is the convolution of the blue and the green, and the mean value of the sum, the value C, is the sum of the mean values of the other two, A and B.

To derive this, we first imagine that quantity B is not distributed but has a single value $x = B$. Given that quantity $A(x)$ is distributed, the sum of $A(x)$ and B must be

$$p_C(x) = p_A(x - B), \tag{A.1}$$

which is the distribution of the quantity A centred at a new value equal to $\overline{A} + B$. We can now add the uncertainty or distribution of the value B: each value of $p_C(x)$ as stated above has a probability $p_B(x)$ of occurring, which must be summed:

$$p_C(x) = \int p_A(x - B) p_B(B) dB. \tag{A.2}$$

This is a convolution of $p_A(x)$ with $p_B(x)$, denoted $p_C(x) = p_A(x) \otimes p_B(x)$. Thus we see that the addition of distributed quantities is the convolution of the distributions. One can show that the mean of the result (C in the graph) is equal to the sum of the means(A and B in the graph). The width is the width of the resulting convolution, which cannot be expressed simply unless the distributions are specified.

A.2. Case for normal distributions: standard error analysis

We define $p_A(x)$ and $p_B(x)$ as normal distributions with standard deviations σ_A and σ_B and means $\overline{A}$ and $\overline{B}$:

$$p_A(x) = \frac{1}{\sqrt{2\pi}\sigma_A} e^{-\frac{(x-\overline{A})^2}{2\sigma_A^2}} \qquad p_B(x) = \frac{1}{\sqrt{2\pi}\sigma_B} e^{-\frac{(x-\overline{B})2}{2\sigma_B^2}} \tag{A.3}$$

Their convolution can be shown to be a normal distribution (it's a bit tedious)[15]:

$$p_C(x) = \frac{1}{\sqrt{2\pi(\sigma_A^2 + \sigma_B^2)}} e^{-\frac{(x - \overline{A} - \overline{B})^2}{2(\sigma_A^2 + \sigma_B^2)}}. \tag{A.4}$$

We see that the new distribution $p_C(x)$ has a mean and standard deviation as follows:

$$\overline{C} = \overline{A} + \overline{B}, \tag{A.5}$$

$$\sigma_C = \sqrt{\sigma_A^2 + \sigma_B^2}. \tag{A.6}$$

This demonstrates the standard root of the sum of the squares of the errors in uncertainty analysis, which requires the assumption of normal distributions.

A.3. Error propagation

Standard error analysis in the experimental sciences dictates how one should combine uncertainty values from different sources across a function, as we do in this work for the calculation of the LCOT and its deviation ΔLCOT. It also corresponds to the result of convolving a series of distributions to one another. If we have a function of several variables $f = f(x, y, z, ...)$ (as in our LCOT in this work, which depends on car prices, fuel costs, O&M costs, all of which are distributed), then the propagation of uncertainties on all variables, Δx, Δy, Δz, ... combine to an uncertainty on Δf following:

$$\Delta f = \sqrt{\left(\frac{\partial f}{\partial x}\right)^2 \Delta x^2 + \left(\frac{\partial f}{\partial y}\right)^2 \Delta y^2 + \left(\frac{\partial f}{\partial z}\right)^2 \Delta z^2 + ...} \tag{A.7}$$

[15] The gaussian is the only function that has the property that maintains its functional form through convolutions, except possibly the Dirac Delta function.

B. List of variables

Δt	Time interval
t	Current year
D	Transport services demand, v·km/y (vehicle-kilometres per year) or t·km/y (tons-kilometres per year)
N	Total transport capacity for one particular service type, in v or t (vehicles or tonnes)
G_i	Transport services generation by technology, p·km/y or t·km/y
U_i	Transport capacity by technology, p (persons or seats) or t (tons)
FF_i	Filling factor, fraction of seats occupied or weight capacity used, in persons per seats (i.e. no units)
d_i	average distance travelled per year by one vehicle of type *i*, km/y
S_i	Technology share of total vehicle capacity
F_{ij}	Investor choice probability matrix
A_{ij}	Technology changeover timescales matrix
G_{ij}	Constraints matrix
CF_i	Capacity factor, km/y
$\overline{CF}$	Share weighted capacity factor, km/y
E_i	Emissions of pollutants in a year, t(pollutant)/y (tons of pollutants per year)
J_i	Fuel use by technology, in GJ/y
α_i^k	Emission factor of technology *i* for pollutant *k*, t(pollutant)/GJ (tons of pollutant per energy content)
β_i	Vehicle fuel consumption, GJ/km
I_i	Vehicle cost, \$/seat
W_i	Cumulative vehicle sales, in seats
B_{ij}	Spillover learning matrix, linking technologies with similar components
C_i	Generalised cost of transportation, in \$/pkm
$f_i(C - C_i, \sigma_i)$dC	Distribution of vehicle purchases with average C_i
$F_(C - C_i)$	Cumulative distribution of vehicle purchases below price C
μ_i	Average in normal cost space
σ_i	Standard deviation in normal cost space
m_i	Average in lognormal space
ν_i	Standard deviation in lognormal space
$LCOT_i$	Levelised cost of transport, in \$/pkm
$\Delta LCOT_i$	STD Levelised cost of transport, in \$/pkm
γ_i	Empirical average value of the 'intangibles', in \$/pkm
FT_i	Fuel tax in \$/pkm
OM_i	Operation and Maintenance costs, in \$/pkm
RT_i	Road tax costs, in \$/pkm
r	discount rate
$n_i(a, t')\Delta a$	Age distribution of the vehicle fleet of technology type *i*
a	Vehicle age in years
t'	Vehicle year of production
ℓ_i	Survival function
p_i	Vehicle instantaneous force of death
τ_i	Vehicle life expectancy
ξ_i	Sales/registrations of new vehicles, in seats
N_i	Total number of vehicles in the fleet of technology type *i*

References

Adner, R., Levinthal, D., 2001. Demand heterogeneity and technology evolution: implications for product and process innovation. Management Science 47 (5), 611–628.

AEA, 2010. Review of cost assumptions and technology uptake scenarios in the ccc transport and macc model. final report. Tech. rep., AEA.

Alam, M. S., Hyde, B., Duffy, P., McNabola, A., 2017. Assessment of pathways to reduce co 2 emissions from passenger car fleets: Case study in ireland. Applied Energy 189, 283–300.

Anderson, S. T., Kellogg, R., Sallee, J. M., 2011. What do consumers believe about future gasoline prices? Tech. rep., National Bureau of Economic Research.

Armenia, S., Baldoni, F., Falsini, D., Taibi, E., 2010. System dynamics energy model for a sustainable transportation system. In: 28th International Conference of the System Dynamics Society. p. 3.

Arthur, W., Ermoliev, Y. M., Kaniovski, Y. M., 1987. Path-dependent processes and the emergence of macro-structure. European Journal of Operational Research 30 (3), 294–303.

Arthur, W. B., 1989. Competing technologies, increasing returns, and lock-in by historical events. The Economic Journal 99 (394), 116–131.

Arthur, W. B., 1994. Increasing returns and path dependence in the economy. University of Michigan Press.

Axsen, J., Kurani, K. S., 2012. Interpersonal influence within car buyers' social networks: applying five perspectives to plug-in hybrid vehicle drivers. Environment and Planning-Part A 44 (5), 1047.

Barker, T., Anger, A., Chewpreecha, U., Pollitt, H., 2012. A new economics approach to modelling policies to achieve global 2020 targets for climate stabilisation. International Review of Applied Economics 26 (2), 205–221.

Barker, T., Pan, H., Köhler, J., Warren, R., Winne, S., 2006. Decarbonizing the global economy with induced technological change: scenarios to 2100 using E3MG. The Energy Journal 27, 143–160.

Barker, T., Scrieciu, S. S., Foxon, T., 2008. Achieving the G8 50% target: Modelling induced and accelerated technological change using the macro-econometric model E3MG. Climate Policy 8 (1), S30–S45.

Barreto, L., Kemp, R., 2008. Inclusion of technology diffusion in energy-systems models: some gaps and needs. Journal of Cleaner Production 16 (1), S95–S101.

Basalla, G., 1988. The evolution of technology. Cambridge University Press.

Baxter, G., Sommerville, I., 2011. Socio-technical systems: From design methods to systems engineering. Interacting with computers 23 (1), 4–17.

Ben-Akiva, M. E., Lerman, S. R., 1985a. Discrete choice analysis: theory and application to travel demand. Vol. 9. MIT press.

Ben-Akiva, M. E., Lerman, S. R., 1985b. Discrete choice analysis: theory and application to travel demand. Vol. 9. MIT press.

Bertram, C., Luderer, G., Pietzcker, R. C., Schmid, E., Kriegler, E., Edenhofer, O., 2015. Complementing carbon prices with technology policies to keep climate targets within reach. Nature Climate Change 5 (3), 235–239.

Bhargava, S. C., 1989. Generalized Lotka-Volterra equations and the mechanism of technological substitution. Technological Forecasting and Social Change 35 (4), 319–326.

Böhringer, C., 1998. The synthesis of bottom-up and top-down in energy policy modeling. Energy Economics 20 (3), 233–248.

Bonabeau, E., 2002. Agent-based modeling: Methods and techniques for simulating human systems. Proceedings of the National Academy of Sciences of the United States of America 99 (Suppl 3), 7280–7287.

Bosetti, V., Carraro, C., Galeotti, M., Massetti, E., Tavoni, M., 2006. A world induced technical change hybrid model. Energy Journal 27.

Brand, C., Anable, J., Tran, M., 2013. Accelerating the transformation to a low carbon passenger transport system: The role of car purchase taxes, feebates, road taxes and scrappage incentives in the uk. Transportation Research Part A: Policy and Practice 49, 132–148.

Brownlee, J., et al., 2007. Complex adaptive systems. Complex Intelligent Systems Laboratory, Centre for Information Technology Research, Faculty of Information Communication Technology, Swinburne University of Technology: Melbourne, Australia.

Bunch, D. S., Ramea, K., Yeh, S., Yang, C., 2015. Incorporating behavioral effects from vehicle choice models into bottom-up energy sector models. Tech. rep.

Burniaux, J.-M., Martin, J. P., Nicoletti, G., Martins, J. O., 1992. Green a multi-sector, multi-region general equilibrium model for quantifying the costs of curbing CO_2 emissions: a technical manual. Tech. rep., OECD Publishing.

Calvin, K., Edmonds, J., Bakken, B., Wise, M., Kim, S., Luckow, P., Patel, P., Graabak, I., 2014. Eu 20-20-20 energy policy as a model for global climate mitigation. Climate policy 14 (5), 581–598.

Capros, P., Georgakopoulos, T., Van Regemorter, D., Proost, S., Schmidt, T., Conrad, K., 1997. European union: the GEM-E3 general equilibrium model. Economic & Financial Modelling 4 (2&3), 51–160.

Capros, P., Mantzos, L., Tasios, N., Kouvaritakis, N., 2009. EU energy trends to 2030. Tech. rep., European Commission.

Car Pages, 2012. 2012 car prices.
URL http://www.carpages.co.uk/car-prices/

Carrillo-Hermosilla, J., Unruh, G. C., 2006. Technology stability and change: an integrated evolutionary approach. Journal of Economic Issues, 707–742.

Criqui, P., Mima, S., Viguier, L., 1999. Marginal abatement costs of CO_2 emission reductions, geographical flexibility and concrete ceilings: an assessment using the POLES model. Energy Policy 27 (10), 585–601.

Daly, H. E., Ramea, K., Chiodi, A., Yeh, S., Gargiulo, M., Gallachóir, B. Ó., 2014. Incorporating travel behaviour and travel time into times energy system models. Applied Energy 135, 429–439.

David, P. A., 1985. Clio and the economics of qwerty. The American Economic Review, 332–337.

David, P. A., 2007. Path dependence: a foundational concept for historical social science. Cliometrica 1 (2), 91–114.

De Vries, B., van Vuuren, D., Den Elzen, M., Janssen, M., 2002. Targets image energy regional (timer) model, technical documentation. Swiss Society of Economics and Statistics.

Deetman, S., Hof, A. F., Girod, B., van Vuuren, D. P., 2015. Regional differences in mitigation strategies: an example for passenger transport. Regional environmental change 15 (6), 987–995.

Deetman, S., Hof, A. F., Pfluger, B., van Vuuren, D. P., Girod, B., van Ruijven, B. J., 2013. Deep greenhouse gas emission reductions in europe: Exploring different options. Energy Policy 55, 152–164.

Dixit, A. K., Stiglitz, J. E., 1977. Monopolistic competition and optimum product diversity. The American Economic Review, 297–308.

Domencich, T. A., McFadden, D., 1975. Urban travel demand - A behavioural analysis. North-Holland Publishing.

Durlauf, S. N., Ioannides, Y. M., 2010. Social interactions. Annu. Rev. Econ. 2 (1), 451–478.

DVLA, 2012a. New car registrations by model 2012, UK car registration agency DVLA. UK Inside Government.
URL https://www.gov.uk/government/statistical-data-sets/veh01-vehicles-registered-for-the-first-time

DVLA, 2012b. Roadside survey of vehicle observations. UK Inside Government.
URL http://data.gov.uk/dataset/roadside-survey-of-vehicles

Ebeling, W., KARMESHU, Scharnhorst, A., 2001. Dynamics of economic and technological search processes in complex adaptive landscapes. Advances in Complex Systems 4 (01), 71–88.

EC, 2008. Sector economic analysis-the times model. http://ipts.jrc.ec.europa.eu/activities/energy-and-transport/TIMES.cfm, accessed: 2013-12-05.

Edenhofer, O., Carraro, C., Kohler, J., Grubb, M., 2006. Endogenous Technological Change and the Economics of Atmospheric Stabilisation, Special Edition, Energy Journal. International Association for Energy Economics.

EIA, 2010. The national energy modeling system : An overview. http://www.eia.gov/oiaf/aeo/overview/, accessed: 2014-04-06.

Elzen, B., Geels, F. W., Green, K., 2004. System innovation and the transition to sustainability: theory, evidence and policy. Edward Elgar Publishing.

Eppstein, M. J., Grover, D. K., Marshall, J. S., Rizzo, D. M., 2011. An agent-based model to study market penetration of plug-in hybrid electric vehicles. Energy Policy 39 (6), 3789–3802.

Euromonitor International, 2012. Euromonitor international passport.
URL http://www.portal.euromonitor.com/Portal/Pages/Magazine/WelcomePage.aspx

Farsi, M., Filippini, M., Pachauri, S., 2007. Fuel choices in urban indian households. Environment and Development Economics 12 (6), 757.

FERMI, F., e Territorio, T. T., FIORELLO, D., 2010. Global Scale System Dynamic Simulation Model for Transport Emissions (GLADYSTE). Leuven.

Fernandez, T., Xavier, B., 2013. Essays on real business cycle modelling under adaptive learning. Ph.D. thesis, University of Glasgow.

Fiorello, D., Fermi, F., Bielanska, D., 2010. The astra model for strategic assessment of transport policies. System Dynamics Review 26 (3), 283–290.

Fisher, J. C., Pry, R. H., 1972. A simple substitution model of technological change. Technological Forecasting and Social Change 3, 75–88.

Forrester, J. W., 1961. Industrial Dynamics. CE Delft.

Freeman, C., Louçã, F., 2002. As time goes by: from the industrial revolutions to the information revolution. OUP Catalogue.

Garnsey, E., McGlade, J., 2006. Complexity and co-evolution: continuity and change in socio-economic systems. Edward Elgar Publishing.

Geels, F., Kemp, R., Dudley, G., Lyons, G., 2011. Automobility in transition: A socio-technical analysis of sustainable transport. Routledge.

Geels, F. W., 2002. Technological transitions as evolutionary reconfiguration processes: a multi-level perspective and a case-study. Research Policy 31 (8), 1257–1274.

Geels, F. W., 2005. The dynamics of transitions in socio-technical systems: A multi-level analysis of the transition pathway from horse-drawn carriages to automobiles (1860 - 1930). Technology Analysis & Strategic Management 17 (4), 445–476.

Geels, F. W., Schot, J., 2007. Typology of sociotechnical transition pathways. Research policy 36 (3), 399–417.

Geroski, P. A., 2000. Models of technology diffusion. Research Policy 29 (4), 603–625.

Gilbert, N., 2008. Agent-Based Models. SAGE.

Gillingham, K., Newell, R. G., Pizer, W. A., 2008. Modeling endogenous technological change for climate policy analysis. Energy Economics 30 (6), 2734–2753.

Goel, R., Mohan, D., Guttikunda, S., Tiwari, G., 2013. Methods to estimate vehicular characteristics in indian cities. Tech. rep., Oak Ridge National Lab., TN (USA).

Goulder, L. H., Schneider, S. H., 1999. Induced technological change and the attractiveness of CO_2 abatement policies. Resource and Energy Economics 21 (3), 211–253.

Grahn, M., Azar, C., Lindgren, K., Berndes, G., Gielen, D., 2007. Biomass for heat or as transportation fuel: A comparison between two model-based studies. Biomass and Bioenergy 31 (11), 747–758.

Grahn, M., Azar, C., Williander, M., Anderson, J. E., Mueller, S. A., Wallington, T. J., 2009. Fuel and vehicle technology choices for passenger vehicles in achieving stringent CO_2 targets: connections between transportation and other energy sectors. Environmental science & technology 43 (9), 3365–3371.

Greene, D. L., 2010. How consumers value fuel economy: A literature review. Tech. rep., Oak Ridge National Laboratory.

Griliches, Z., 1957. Hybrid corn: An exploration in the economics of technological change. Econometrica, Journal of the Econometric Society, 501–522.

Grubb, M., 2014. Planetary economics: energy, climate change and the three domains of sustainable development. Routledge.

Grubb, M., Köhler, J., Anderson, D., 2002. Induced technical change in energy and environmental modeling: Analytic approaches and policy implications. Annual Review of Energy and the Environment 27 (1), 271–308.

Grubler, A., 1990. The rise and fall of infrastructures: dynamics of evolution and technological change in transport. IIASA.

Grubler, A., Cleveland, C. J., 2008. Energy transitions. Encyclopedia of Earth.

Gül, T., Kypreos, S., Turton, H., Barreto, L., 2009. An energy-economic scenario analysis of alternative fuels for personal transport using the Global Multi-regional MARKAL model (GMM). Energy 34 (10), 1423–1437.

Hall, B. H., Khan, B., 2003. Adoption of new technology. Tech. rep., National Bureau of Economic Research.

Hao, H., Wang, H., Ouyang, M., Cheng, F., 2011. Vehicle survival patterns in china. Science China Technological Sciences 54 (3), 625–629.

Harrison, M., Commission, A. P., et al., 2010. Valuing the Future: the social discount rate in cost-benefit analysis. Productivity Commission Canberra.

Heaps, C., 2012. Long-Range Energy Alternatives Planning (Leap) system.[Software Version 2011.0043]. Somerville MA, USA: Stockholm Environment Institute.
Hedenus, F., Azar, C., Lindgren, K., 2005. Induced technological change in a limited foresight optimization model. Energy 34 (10), 1423–1437.
Herbst, A., Toro, F., Reitze, F., Jochem, E., 2012. Introduction to energy systems modelling. Swiss Society of Economics and Statistics.
Hofbauer, J., Sigmund, K., 1998. Evolutionary Games and Population Dynamics. Cambridge University Press, Cambridge, UK.
Holmgren, J., Davidsson, P., Persson, J. A., Ramstedt, L., 2012. Tapas: A multi-agent-based model for simulation of transport chains. Simulation Modelling Practice and Theory 23, 1–18.
Horrace, W. C., Huang, R., Perloff, J. M., 2009. Variety: Consumer choice and optimal diversity. Tech. rep., University of Connecticut.
Hourcade, J.-C., 1993. Modelling long-run scenarios: methodology lessons from a prospective study on a low CO_2 intensive country. Energy Policy 21 (3), 309–326.
Hourcade, J. C., Jaccard, M., Bataille, C., Ghersi, F., et al., 2006. Hybrid modeling: New answers to old challenges. The Energy Journal 2 (Special issue), 1–12.
Howey, D., North, R., MARTINEZ-BOTAS, R., 2010. Road transport technology and climate change mitigation. Grantham Institute for Climate Change Briefing Paper 10 (2).
Huo, H., Wang, M., 2012. Modeling future vehicle sales and stock in China. Energy Policy 43, 17–29.
IEA, 2007. World Energy Model. http://www.worldenergyoutlook.org/weomodel/, accessed: 2013-12-06.
IEA, 2012. ETSAP TECHDS Energy Technology Briefs. http://www.iea-etsap.org/web/Demand.asp, accessed: 2013-02-05.
IEA, 2013. Global EV Outlook. Understanding the EV Landscape to 2020. Tech. rep., IEA/OECD.
IIASA, 2003. IIASA Myopic Message. http://www.iiasa.ac.at/web/home/research/modelsData/MESSAGE/MYOPIC-MESSAGE.en.html, accessed: 2013-12-06.
IIASA, 2010. MESSAGE Model: A modeling framework for medium- to long-term energy system planning, energy policy analysis, and scenario development. http://www.iiasa.ac.at/web/home/research/modelsData/MESSAGE/MESSAGE.en.html, accessed: 2013-12-05.
Inderwildi, O., King, D., 2012. Energy, Transport & the Environment. Springer.
Jaccard, M., Nyboer, J., Bataille, C., Sadownik, B., 2003. Modeling the cost of climate policy: distinguishing between alternative cost definitions and long-run cost dynamics. Energy Journal 24 (1), 49–73.
Jacobsson, S., Bergek, A., 2004. Transforming the energy sector: the evolution of technological systems in renewable energy technology. Industrial and Corporate Change 13 (5), 815–849.
Jebaraj, S., Iniyan, S., 2006. A review of energy models. Renewable and Sustainable Energy Reviews 10 (4), 281–311.
Kamakate, F., Schipper, L., 2009. Trends in truck freight energy use and carbon emissions in selected oecd countries from 1973 to 2005. Energy Policy 37 (10), 3743–3751.
Karkatsoulis, P., Siskos, P., Paroussos, L., Capros, P., 2016. Simulating deep co 2 emission reduction in transport in a general equilibrium framework: the gem-e3t model. Transportation Research Part D: Transport and Environment.
Kemp, R., 1994. Technology and the transition to environmental sustainability: the problem of technological regime shifts. Futures 26 (10), 1023–1046.
Kemp, R., Rip, A., Schot, J., 2001. Constructing transition paths through the management of niches. Lawrence Erlbaum.
Kloess, M., Müller, A., 2011. Simulating the impact of policy, energy prices and technological progress on the passenger car fleet in austria: A model based analysis 2010–2050. Energy Policy 39 (9), 5045–5062.
Knobloch, F., Mercure, J.-F., 2016. The behavioural aspect of green technology investments: a general positive model in the context of heterogeneous agents. 4CMR working papers.
URL http://be.4cmr.group.cam.ac.uk/working-papers/pdf/4CMR_WP_11.pdf
Köhler, J., Grubb, M., Popp, D., Edenhofer, O., 2006. The transition to endogenous technical change in climate-economy models: a technical overview to the innovation modeling comparison project. Energy Journal 27, 17–55.
Köhler, J., Whitmarsh, L., Nykvist, B., Schilperoord, M., Bergman, N., Haxeltine, A., 2009. A transitions model for sustainable mobility. Ecological Economics 68 (12), 2985–2995.
Kypreos, S., 2005. Modeling experience curves in MERGE (model for evaluating regional and global effects). Energy 30 (14), 2721–2737.
Kypreos, S., Bahn, O., 2003. A MERGE model with endogenous technological progress. Environmental Modeling & Assessment 8 (3), 249–259.
Lee, S., Kim, M.-S., Park, Y., 2009. ICT Co-evolution and Korean ICT strategy: An analysis based on patent data. Telecommunications Policy 33 (5), 253–271.
Leimbach, M., Bauer, N., Baumstark, L., Edenhofer, O., 2010. Mitigation costs in a globalized world: climate policy analysis with REMIND-R. Environmental Modeling & Assessment 15 (3), 155–173.
Leonard, R. D., Jones, G. T., 1989. Quantifying diversity in archaeology. Cambridge University Press.
Levinthal, D., 1998. The slow pace of rapid technological change: gradualism and punctuation in technological change. Industrial and Corporate Change 7 (2), 217–247.
Li, F. G., 2017. Actors behaving badly: Exploring the modelling of non-optimal behaviour in energy transitions. Energy Strategy Reviews 15, 57–71.
Liimatainen, H., Arvidsson, N., Hovi, I. B., Jensen, T. C., Nykänen, L., 2014. Road freight energy efficiency and co 2 emissions in the nordic countries. Research in Transportation Business & Management 12, 11–19.
Liu, X., 2012. Survival Analysis. Wiley.
Löschel, A., 2002. Technological change in economic models of environmental policy: a survey. Ecological Economics 43 (2), 105–126.
Maconachie, R., Tanko, A., Zakariya, M., 2009. Descending the energy ladder: Oil price shocks and domestic fuel choices in kano, nigeria. Land use policy 26 (4), 1090–1099.
Mansfield, E., 1961. Technical change and the rate of imitation. Econometrica 29 (4), pp. 741–766.
Marchetti, C., Nakicenovic, N., Volkswagenwerk, S., 1980. The dynamics of energy systems and the logistic substitution model. International Institute for Applied Systems Analysis.
Martinsen, D., Krey, V., 2008. Compromises in energy policy: Using fuzzy optimization in an energy systems model. Energy Policy 36 (8),

2983–2994.
Mau, P., Eyzaguirre, J., Jaccard, M., Collins-Dodd, C., Tiedemann, K., 2008. The 'neighbor effect': Simulating dynamics in consumer preferences for new vehicle technologies. Ecological Economics 68 (1), 504–516.
McCollum, D., Krey, V., Kolp, P., Nagai, Y., Riahi, K., 2013. Transport electrification: A key element for energy system transformation and climate stabilization. Climatic Change, 1–14.
McCollum, D., Yang, C., 2009. Achieving deep reductions in us transport greenhouse gas emissions: Scenario analysis and policy implications. Energy Policy 37 (12), 5580–5596.
McCollum, D. L., Wilson, C., Pettifor, H., Ramea, K., Krey, V., Riahi, K., Bertram, C., Lin, Z., Edelenbosch, O. Y., Fujisawa, S., 2016a. Improving the behavioral realism of global integrated assessment models: An application to consumers' vehicle choices. Transportation Research Part D: Transport and Environment.
McCollum, D. L., Wilson, C., Pettifor, H., Ramea, K., Krey, V., Riahi, K., Bertram, C., Lin, Z., Edelenbosch, O. Y., Fujisawa, S., 2016b. Improving the behavioral realism of global integrated assessment models: An application to consumers' vehicle choices. Transportation Research Part D: Transport and Environment.
McDowall, W., 2012. Endogenous technology learning fro hydrogen and fuel cell technology in UKSHEC II: Literature reveiw, research questions and data ukshec. Tech. rep., Working Paper No. 8 UCL Energy Institute, University College London.
McShane, B. B., Bradlow, E. T., Berger, J., 2012. Visual influence and social groups. Journal of Marketing Research 49 (6), 854–871.
Mercure, J.-F., 2012. Ftt:power : A global model of the power sector with induced technological change and natural resource depletion. Energy Policy 48 (0), 799 – 811.
URL http://dx.doi.org/10.1016/j.enpol.2012.06.025
Mercure, J.-F., 2015. An age structured demographic theory of technological change. J. Evol. Econ. 25, 787–820.
URL http://arxiv.org/abs/1304.3602
Mercure, J.-F., 2016a. Fashion, fads and the popularity of choices: micro-foundations for non-equilibrium consumer theory. arXiv preprint arXiv:1209.0424.
Mercure, J.-F., 2016b. Fashion, fads and the popularity of choices: micro-foundations of non-equilibrium consumer theory. arXiv:1607.04155.
URL https://arxiv.org/abs/1607.04155
Mercure, J.-F., Lam, A., 2015a. The effectiveness of policy on consumer choices for private road passenger transport emissions reductions in six major economies. Environmental Research Letters 10 (6), 064008.
Mercure, J.-F., Lam, A., 2015b. The effectiveness of policy on consumer choices for private road passenger transport emissions reductions in six major economies. Environ. Res. Lett. 10 (064008).
Mercure, J.-F., Pollitt, H., Bassi, A. M., Viñuales, J. E., Edwards, N. R., 2016a. Modelling complex systems of heterogeneous agents to better design sustainability transitions policy. Global Environmental Change 37, 102–115.
Mercure, J.-F., Pollitt, H., Bassi, A. M., Vinuales, J. E., Edwards, N. R., 2016b. Modelling complex systems of heterogenous agents to better design sustainability transitions policy. Global Environmental Change 37, 102 – 115.
Mercure, J.-F., Pollitt, H., Edwards, N., Holden, P., Chewpreecha, U., Salas, P., Lam, A., Knobloch, F., Vinuales, J., 2017. Environmental impact assessment for climate change policy with the simulation-based integrated assessment model E3ME-FTT-GENIE. ArXiv e-prints.
URL https://arxiv.org/abs/1707.04870
Mercure, J.-F., Salas, P., 2013. On the global economic potentials and marginal costs of non-renewable resources and the price of energy commodities. Energy Policy (63), 469 – 483.
URL http://dx.doi.org/10.1016/j.enpol.2013.08.040
Mercure, J.-F., Salas, P., Foley, A., Chewpreecha, U., Pollitt, H., Holden, P. B., Edwards, N. R., 2014. The dynamics of technology diffusion and the impacts of climate policy instruments in the decarbonisation of the global electricity sector. To appear in Energy Policy.
URL http://arxiv.org/abs/1309.7626
Messner, A., 1995. User's Guide for MESSAGE III. Tech. rep., IIASA.
Metcalfe, J. S., 2004. Ed mansfield and the diffusion of innovation: An evolutionary connection. The Journal of Technology Transfer 30, 171–181.
Metcalfe, J. S., Miles, I., 1994. Standards, selection and variety: an evolutionary approach. Information Economics and Policy 6 (3), 243–268.
Mittal, S., Dai, H., Fujimori, S., Hanaoka, T., Zhang, R., 2016. Key factors influencing the global passenger transport dynamics using the aim/transport model. Transportation Research Part D: Transport and Environment.
Moksnes, N., Welsch, M., Gardumi, F., Shivakumar, A., Broad, O., Howells, M., Taliotis, C., Sridharan, V., 2015. 2015 osemosys user manual. Tech. rep., KTH.
URL http://www.osemosys.org/uploads/1/8/5/0/18504136/new-website_osemosys_manual_-_working_with_text_files_-_2015-11-05.pdf
Morris, S. A., Pratt, D., 2003. Analysis of the Lotka–Volterra competition equations as a technological substitution model. Technological Forecasting and Social Change 70 (2), 103–133.
Motorcycle News, 2012. 2012 Motorcycle News.
URL http://www.Motorcyclenews.com/mcn/bikereviews
Mueller, M. G., de Haan, P., 2009. How much do incentives affect car purchase? Agent-based microsimulation of consumer choice of new cars: Part I: Model structure, simulation of bounded rationality, and model validation. Energy Policy 37 (3), 1072–1082.
Nakata, T., Silva, D., Rodionov, M., 2011. Application of energy system models for designing a low-carbon society. Progress in Energy and Combustion Science 37 (4), 462–502.
Nakicenovic, N., 1986. The automobile road to technological change: diffusion of the automobile as a process of technological substitution. Technological Forecasting and Social Change 29 (4), 309–340.
Navius, 2010. CIMS Community: Energy and Emissions Forecasting For Communities in British Columbia. http://www.naviusresearch.com/data/pages/CIMScommunity.php, accessed: 2013-12-06.
Nemet, G. F., 2009. Demand-pull, technology-push, and government-led incentives for non-incremental technical change. Research Policy 38 (5), 700–709.

Nguene, G., Fragnière, E., Kanala, R., Lavigne, D., Moresino, F., 2011a. Socio-markal: Integrating energy consumption behavioral changes in the technological optimization framework. Energy for Sustainable Development 15 (1), 73–83.

Nguene, G., Fragnière, E., Kanala, R., Lavigne, D., Moresino, F., 2011b. Socio-markal: Integrating energy consumption behavioral changes in the technological optimization framework. Energy for Sustainable Development 15 (1), 73–83.

Nill, J., 2008. Diffusion as time-dependent result of technological evolution, competition, and policies: the case of cleaner iron and steel technologies. Journal of Cleaner Production 16 (1), S58–S66.

Nill, J., Kemp, R., 2009. Evolutionary approaches for sustainable innovation policies: From niche to paradigm. Research Policy 38 (4), 668–680.

Nyqvist, B., 2005. Limited foresight in a linear cost minimisation model of the global energy system. Gotherburg: Master Thesis in Complex Adaptive Systems, Physical Resource Theory, Chalmers University of Technology.

Pachauri, S., van Ruijven, B. J., Nagai, Y., Riahi, K., van Vuuren, D. P., Brew-Hammond, A., Nakicenovic, N., 2013. Pathways to achieve universal household access to modern energy by 2030. Environmental Research Letters 8 (2), 024015.

Pettifor, H., Wilson, C., Axsen, J., Abrahamse, W., Anable, J., 2017a. Social influence in the global diffusion of alternative fuel vehicles–a meta-analysis. Journal of Transport Geography 62, 247–261.

Pettifor, H., Wilson, C., McCollum, D., Edelenbosch, O., 2017b. Modelling social influence and cultural variation in global low-carbon vehicle transitions. Global Environmental Change 47, 76–87.

Pielou, E. C., 1977. Mathematical Ecology. [2d ed.]. John and Sons.

Rogers, E. M., 2010. Diffusion of innovations. Simon and Schuster.

Rogers, E. M., Kim, J.-I., 1985. Diffusion of innovations in public organizations. Innovation in the Public Sector, 85–108.

Safarzynska, K., van den Bergh, J. C. J. M., 2010. Evolutionary models in economics: a survey of methods and building blocks. Journal of Evolutionary Economics 20 (3), 329–373.

Sarkar, J., 1998. Technological diffusion: alternative theories and historical evidence. Journal of Economic Surveys 12 (2), 131–176.

Saviotti, P., Metcalfe, J. S., 1991. Evolutionary theories of economic and technological change: present status and future prospects. Harwood Academic Publishers.

Saviotti, P. P., Mani, G., 1995. Competition, variety and technological evolution: a replicator dynamics model. Journal of Evolutionary Economics 5 (4), 369–392.

Schaefer, A., Jacoby, H. D., 2005. Technology detail in a multisector CGE model: transport under climate policy. Energy Economics 27 (1), 1–24.

Schumpeter, J. A., 1942. Socialism, capitalism and democracy. Harper and Brothers.

Simon, H. A., 1972. Theories of bounded rationality. Decision and Oganization 1, 161–176.

Siskos, P., Capros, P., De Vita, A., 2015. Co 2 and energy efficiency car standards in the eu in the context of a decarbonisation strategy: a model-based policy assessment. Energy Policy 84, 22–34.

Slater, S. F., Mohr, J. J., 2006. Successful development and commercialization of technological innovation: insights based on strategy type. Journal of Product Innovation Management 23 (1), 26–33.

Smith, W. R., 1956. Product differentiation and market segmentation as alternative marketing strategies. The Journal of Marketing, 3–8.

Stirling, A., 1994. Diversity and ignorance in electricity supply investment: addressing the solution rather than the problem. Energy Policy 22 (3), 195–216.

Stirling, A., 2007. A general framework for analysing diversity in science, technology and society. Journal of the Royal Society Interface 4 (15), 707–719.

Takeshita, T., Yamaji, K., 2008. Important roles of Fischer–Tropsch synfuels in the global energy future. Energy Policy 36 (8), 2773–2784.

Tanaka, M., Ida, T., Murakami, K., Friedman, L., 2014. Consumers' willingness to pay for alternative fuel vehicles: A comparative discrete choice analysis between the us and japan. Transportation Research Part A: Policy and Practice 70, 194–209.

Turner, J., 1992. Stochastic processes in populations: the horse behind the cart. Genes in Ecology. Blackwell Scientific, Oxford, UK, 29–53.

Urry, J., 2004. The system of automobility. Theory, Culture & Society 21 (4-5), 25–39.

van der Zwaan, B. C., Gerlagh, R., Schrattenholzer, L., et al., 2002. Endogenous technological change in climate change modelling. Energy economics 24 (1), 1–19.

Van Herbruggen, B., Logghe, S., 2005. TREMOVE version 2.3 simulation model for European environmental transport policy analysis. In: Proc. 14 th Int. Symp. Transport and Air Pollution, Graz. pp. 1–3.

Wegener, M., 2004. Overview of land-use transport models. Handbook of Transport Geography and Spatial Systems 5, 127–146.

Weiss, M., Patel, M., Junginger, M., Perujo, A., Bonnel, P., van Grootveld, G., 2012. On the electrification of road transport-learning rates and price forecasts for hybrid-electric and battery-electric vehicles. Energy Policy 48, 374–393.

Wells, P., Nieuwenhuis, P., 2012. Transition failure: Understanding continuity in the automotive industry. Technological Forecasting and Social Change.

Wells, P., Nieuwenhuis, P., Orsato, R., 2010. The nature and causes of inertia in the automotive industry: regime stability and non-change. Automobility in Transition.

Weyant, J. P., Olavson, T., 1999. Issues in modeling induced technological change in energy, environmental, and climate policy. Environmental Modeling and Assessment 4 (1), 67.

Wilson, C., Grubler, A., Bauer, N., Krey, V., Riahi, K., 2012. Future capacity growth of energy technologies: are scenarios consistent with historical evidence? Climatic Change, 1–15.

Wilson, C., Grubler, A., Bauer, N., Krey, V., Riahi, K., 2013. Future capacity growth of energy technologies: are scenarios consistent with historical evidence? Climatic Change 118 (2), 381–395.

Wilson, C., Pettifor, H., McCollum, D., 2015. Improving the behavioural realism of integrated assessment models of global climate change mitigation: a research agenda. advance project deliverable no. 3.2.

Winter, S., Nelson, R., 1982. An evolutionary theory of economic change. University of Illinois at Urbana-Champaign's Academy for Entrepreneurial Leadership Historical Research Reference in Entrepreneurship.

Yeh, S., McCollum, D., 2011. Optimizing the transportation climate mitigation wedge. Sustainable Transportation Energy Pathways, Institute for Transportation Studies, UC Davis, California.

Yin, X., Chen, W., Eom, J., Clarke, L. E., Kim, S. H., Patel, P. L., Yu, S., Kyle, G. P., 2015. China's transportation energy consumption and co 2 emissions from a global perspective. Energy Policy 82, 233–248.

Zachariadis, T., 2005. Assessing policies towards sustainable transport in europe: an integrated model. Energy Policy 33 (12), 1509–1525.

Zachariadis, T., Samaras, Z., Zierock, K.-H., 1995. Dynamic modeling of vehicle populations: an engineering approach for emissions calculations. Technological Forecasting and Social Change 50 (2), 135–149.

Zhuang, J., Liang, Z., Lin, T., De Guzman, F., 2007. Theory and practice in the choice of social discount rate for cost-benefit analysis: a survey. Research Policy 38 (6), 1066–1076.
